# Supplementary material for: LOAd703-induced tumor microenvironment gene engineering in combination with atezolizumab in metastatic malignant melanoma: a phase I/II trial
Source: Nat Commun. 2026 Feb 16;17:1760. doi: 10.1038/s41467-026-69629-0 (PMC12913765; doi:10.1038/s41467-026-69629-0)
Supplement: Supplementary file 1 — Supplementary Information [file 41467_2026_69629_MOESM1_ESM.pdf]

SUPPLEMENTARY INFORMATION

LOAd703-Induced Tumor Microenvironment Gene Engineering in Combination with Atezolizumab in Metastatic Malignant Melanoma – A Phase I/II Trial

Omid Hamid[1*], Viktoria Ekström-Rydén[2,3*], Inderjit Mehmi[1], Daniel Wang[4#], Meera Patel[4], Salem Alsaqal[5], Sandra Irenaeus[2,3], Linda C Sandin[6], Clara Nordström[6], Hanna Grauers Wiktorin[2], Tanja Lövgren[2], Emma Eriksson[2,6], Justyna Leja-Jarblad[6], Angelica Loskog[2,6¤§] and Gustav J. Ullenhag[2,3¤]
*These authors contributed equally
[1]The Angeles Clinic and Rese[¤] These authors jointly supervised this work
[§]Corresponding author

[1]The Angeles Clinic and Research Institute, A Cedars Sinai Affiliate, Medical Oncology, Los Angeles, CA, USA.
[2]Department of Immunology, Genetics and Pathology, Science for Life Laboratory, Uppsala University, Uppsala, Sweden.
[3]Department of Oncology, Uppsala University Hospital, Uppsala, Sweden.
[4]Baylor College of Medicine, McNair Campus, Houston, TX, USA.
[5]Department of Surgical Sciences, Radiology & Molecular Imaging, Uppsala University, Uppsala, Sweden.
[6]Lokon Pharma AB, Uppsala, Sweden.
[#]Current position Department of Internal Medicine at UT Southwestern Medical Center and the Division of Hematology and Oncology.

Table of Contents

Supplementary Figure 1: Study Overview.......3

Supplementary Figure 2: Imaging.......4

Supplementary Table 1: Adverse events (n=24)[a] 5

Supplementary Table 2: Serious adverse events (n=24)[a] 7

Supplementary Table 3: Significant alterations in gene expression between < median OS and > median OS in patients at baseline 8

Supplementary Table 4: Significant alterations in gene expression between < median OS and > median OS in patients post-treatment (week 9) 10

Supplementary Table 5: Significant alterations in protein expression between pre- and post-treatment (week 9) serum samples, Immune oncology panel.......11

Supplementary Table 6: Significant alterations in protein expression between pre- and post-treatment (week 9) serum samples, Oncology panel.......12

Supplementary Table 7: Best overall metabolic response.......13

Supplementary Table 8. Cox Regression Analysis 14

Supplementary Note: Study Protocol

Supplementary Figure 1: Study Overview

A

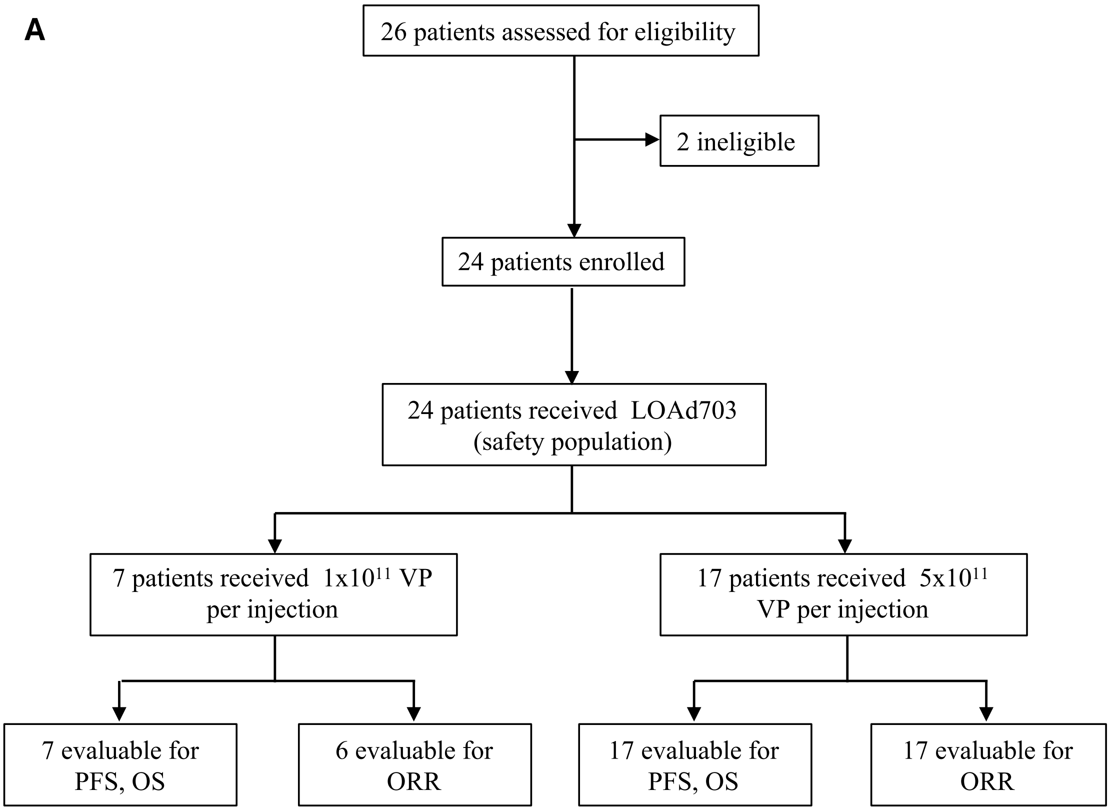

B

Weeks	0	3	6	9	12	15	18	21	24	27	30	33	36	39	42	45	48	51	54	57
LOAd703	●	●	●	●	●	●	●	●	●	●	●	●								
Atezolizumab	●	●	●	●	●	●	●	●	●	●	●	●	●	●	●	●	●	●	●	
Imaging: ORR	●			●			●			●			●			●				●
Biopsy: Biomarkers	●			●																
Blood: Biomarkers	●			●																
Blood: ADA	● ○	●	●	● ○			○	●		○			○			○				● ○

Supplementary Figure 1: Study overview

A: Consort diagram illustrating the recruitment process. B: Study outline of treatments, response evaluations, sampling for tumor and blood biomarker analysis and anti-drug antibodies (ADAs) as presented herein. ADA: open circles represent sampling times for anti-adenovirus antibodies and closed circles for anti-atezolizumab antibodies.

Supplementary Figure 2: Imaging

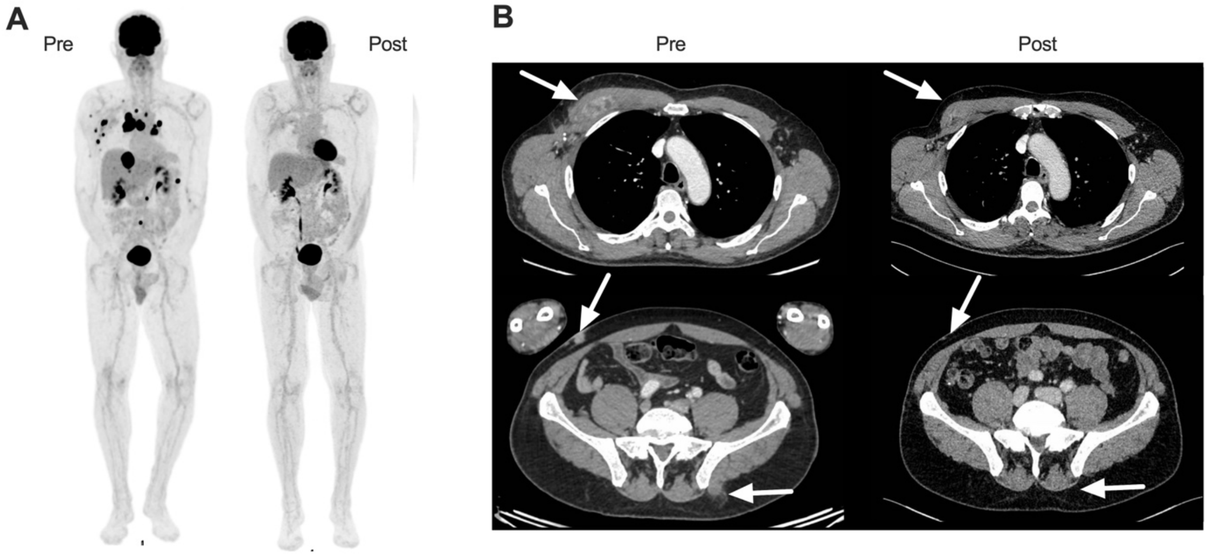

Supplementary Figure 2: Imaging

A: PET images of a male with melanoma metastases in the liver, lung hila, axilla and subcutis before treatment initiation (left image) and after treatment (right image). B: CT images of a male with melanoma metastases in the pectoral muscle (top left) and subcutis (bottom left) before treatment (top right) and after (bottom right).

Supplementary Table 1: Adverse events (n=24)[a]

Preferred Term	Total, N (%) of Subjects with AE					Total Number of AEs
	Grade 1-2	Grade 3	Grade 4	Grade 5	Any grade	
Pyrexia	15 (63)	1 (4)	0 (0)	0 (0)	15 (63)	24
Nausea	8 (33)	0 (0)	0 (0)	0 (0)	8 (33)	11
Vomiting	7 (29)	0 (0)	0 (0)	0 (0)	7 (29)	7
Fatigue	4 (17)	2 (8)	0 (0)	0 (0)	6 (25)	6
Chills	5 (21)	0 (0)	0 (0)	0 (0)	5 (21)	7
Headache	5 (21)	0 (0)	0 (0)	0 (0)	5 (21)	9
Infusion related reaction	5 (21)	0 (0)	0 (0)	0 (0)	5 (21)	8
Anaemia	2 (8)	2 (8)	1 (4)	0 (0)	4 (17)	7
Cytokine release syndrome	4 (17)	0 (0)	0 (0)	0 (0)	4 (17)	4
Diarrhoea	4 (17)	0 (0)	0 (0)	0 (0)	4 (17)	4
Myalgia	3 (13)	0 (0)	0 (0)	0 (0)	3 (13)	3
Nasopharyngitis	3 (13)	0 (0)	0 (0)	0 (0)	3 (13)	3
Pain in extremity	3 (13)	0 (0)	0 (0)	0 (0)	3 (13)	3
Rash	3 (13)	0 (0)	0 (0)	0 (0)	3 (13)	3
Tumour pain	2 (8)	1 (4)	0 (0)	0 (0)	3 (13)	3
Decreased appetite	2 (8)	0 (0)	0 (0)	0 (0)	2 (8)	2
Hiccups	2 (8)	0 (0)	0 (0)	0 (0)	2 (8)	2
Hyperglycaemia	2 (8)	0 (0)	0 (0)	0 (0)	2 (8)	3
Hyponatraemia	2 (8)	0 (0)	0 (0)	0 (0)	2 (8)	2
Injection site pain	2 (8)	0 (0)	0 (0)	0 (0)	2 (8)	2
Lymphocyte count decreased	2 (8)	0 (0)	0 (0)	0 (0)	2 (8)	2
Pruritus	2 (8)	0 (0)	0 (0)	0 (0)	2 (8)	2
Tachycardia	2 (8)	0 (0)	0 (0)	0 (0)	2 (8)	3
Abdominal abscess	0 (0)	1 (4)	0 (0)	0 (0)	1 (4)	1
Abdominal pain	1 (4)	0 (0)	0 (0)	0 (0)	1 (4)	1
Activated partial thromboplastin time prolonged	1 (4)	0 (0)	0 (0)	0 (0)	1 (4)	1
Acute kidney injury	0 (0)	1 (4)	0 (0)	0 (0)	1 (4)	1
Adrenal insufficiency	1 (4)	0 (0)	0 (0)	0 (0)	1 (4)	1
Alanine aminotransferase increased	1 (4)	0 (0)	0 (0)	0 (0)	1 (4)	1
Angina pectoris	1 (4)	0 (0)	0 (0)	0 (0)	1 (4)	1
Aspartate aminotransferase increased	1 (4)	0 (0)	0 (0)	0 (0)	1 (4)	1
Back pain	1 (4)	0 (0)	0 (0)	0 (0)	1 (4)	1
Blood albumin decreased	1 (4)	0 (0)	0 (0)	0 (0)	1 (4)	1
Blood creatinine increased	1 (4)	0 (0)	0 (0)	0 (0)	1 (4)	1
Borrelia test positive	1 (4)	0 (0)	0 (0)	0 (0)	1 (4)	1
Cancer pain	1 (4)	0 (0)	0 (0)	0 (0)	1 (4)	1
Cardiac failure	0 (0)	1 (4)	0 (0)	0 (0)	1 (4)	1
Contusion	1 (4)	0 (0)	0 (0)	0 (0)	1 (4)	1
Cough	1 (4)	0 (0)	0 (0)	0 (0)	1 (4)	1
Deep vein thrombosis	1 (4)	1 (4)	0 (0)	0 (0)	1 (4)	5
Dizziness	1 (4)	0 (0)	0 (0)	0 (0)	1 (4)	1
Dysgeusia	1 (4)	0 (0)	0 (0)	0 (0)	1 (4)	1
Dysphonia	1 (4)	0 (0)	0 (0)	0 (0)	1 (4)	1
Dyspnoea	1 (4)	0 (0)	0 (0)	0 (0)	1 (4)	1
Embolism	1 (4)	0 (0)	0 (0)	0 (0)	1 (4)	1
Erythema	1 (4)	0 (0)	0 (0)	0 (0)	1 (4)	1
General physical health deterioration	0 (0)	0 (0)	0 (0)	1 (4)	1 (4)	1
Groin pain	1 (4)	0 (0)	0 (0)	0 (0)	1 (4)	1
Haematuria	1 (4)	0 (0)	0 (0)	0 (0)	1 (4)	1

Haemoptysis	1 (4)	0 (0)	0 (0)	0 (0)	1 (4)	1
Hypercalcaemia	1 (4)	0 (0)	0 (0)	0 (0)	1 (4)	1
Hypertension	1 (4)	0 (0)	0 (0)	0 (0)	1 (4)	1
Hypocalcaemia	1 (4)	0 (0)	0 (0)	0 (0)	1 (4)	1
Hypoglycaemia	1 (4)	0 (0)	0 (0)	0 (0)	1 (4)	1
Hypotension	1 (4)	0 (0)	0 (0)	0 (0)	1 (4)	1
Hypoxia	1 (4)	0 (0)	0 (0)	0 (0)	1 (4)	1
Infected neoplasm	0 (0)	1 (4)	0 (0)	0 (0)	1 (4)	1
Inflammation	1 (4)	0 (0)	0 (0)	0 (0)	1 (4)	1
Injection site reaction	0 (0)	1 (4)	0 (0)	0 (0)	1 (4)	1
Leukocytosis	0 (0)	1 (4)	0 (0)	0 (0)	1 (4)	1
Lymphoedema	0 (0)	1 (4)	0 (0)	0 (0)	1 (4)	1
Medication error	0 (0)	1 (4)	0 (0)	0 (0)	1 (4)	1
Muscle spasms	1 (4)	0 (0)	0 (0)	0 (0)	1 (4)	1
Muscular weakness	1 (4)	0 (0)	0 (0)	0 (0)	1 (4)	1
Nasal congestion	1 (4)	0 (0)	0 (0)	0 (0)	1 (4)	1
Neuropathy peripheral	1 (4)	0 (0)	0 (0)	0 (0)	1 (4)	1
Neutrophil count decreased	1 (4)	0 (0)	0 (0)	0 (0)	1 (4)	1
Non-cardiac chest pain	0 (0)	1 (4)	0 (0)	0 (0)	1 (4)	1
Oedema peripheral	1 (4)	0 (0)	0 (0)	0 (0)	1 (4)	1
Pain	1 (4)	0 (0)	0 (0)	0 (0)	1 (4)	1
Paraesthesia	1 (4)	0 (0)	0 (0)	0 (0)	1 (4)	1
Pneumonia	1 (4)	0 (0)	0 (0)	0 (0)	1 (4)	1
Proteinuria	1 (4)	0 (0)	0 (0)	0 (0)	1 (4)	1
Rib fracture	1 (4)	0 (0)	0 (0)	0 (0)	1 (4)	1
Salmonellosis	0 (0)	1 (4)	0 (0)	0 (0)	1 (4)	1
Sepsis	0 (0)	0 (0)	1 (4)	0 (0)	1 (4)	1
Sinus tachycardia	1 (4)	0 (0)	0 (0)	0 (0)	1 (4)	1
Skin disorder	1 (4)	0 (0)	0 (0)	0 (0)	1 (4)	1
Thrombosis	1 (4)	0 (0)	0 (0)	0 (0)	1 (4)	1
Tooth abscess	1 (4)	0 (0)	0 (0)	0 (0)	1 (4)	1
Troponin I increased	1 (4)	0 (0)	0 (0)	0 (0)	1 (4)	1
Vaginal haemorrhage	1 (4)	0 (0)	0 (0)	0 (0)	1 (4)	1
White blood cell count decreased	1 (4)	0 (0)	0 (0)	0 (0)	1 (4)	2

Data are n (%) unless otherwise specified. Worst grade is included in the table.
Abbreviations: AE, Adverse event.
[a]Safety-evaluable patients receiving at least one dose of LOAd703. Database lock December 14, 2023.

Supplementary Table 2: Serious adverse events (n=24)[a]

Preferred Term	Total, N (%) of Subjects with SAE					Total Number of SAEs
	Grade 1-2	Grade 3	Grade 4	Grade 5	Any grade	
Abdominal abscess	0 (0)	1 (4)	0 (0)	0 (0)	1 (4)	1
Acute kidney injury	0 (0)	1 (4)	0 (0)	0 (0)	1 (4)	1
Anaemia	0 (0)	0 (0)	1 (4)	0 (0)	1 (4)	1
Cardiac failure	0 (0)	1 (4)	0 (0)	0 (0)	1 (4)	1
Cytokine release syndrome	1 (4)[b]	0 (0)	0 (0)	0 (0)	1 (4)	1
Deep vein thrombosis	0 (0)	1 (4)	0 (0)	0 (0)	1 (4)	3
General physical health deterioration	0 (0)	0 (0)	0 (0)	1 (4)	1 (4)	1
Infected neoplasm	0 (0)	1 (4)	0 (0)	0 (0)	1 (4)	1
Lymphoedema	0 (0)	1 (4)	0 (0)	0 (0)	1 (4)	1
Medication error	0 (0)	1 (4)	0 (0)	0 (0)	1 (4)	1
Non-cardiac chest pain	0 (0)	1 (4)	0 (0)	0 (0)	1 (4)	1
Pyrexia	1 (4)[b]	0 (0)	0 (0)	0 (0)	1 (4)	1
Salmonellosis	0 (0)	1 (4)	0 (0)	0 (0)	1 (4)	1
Sepsis	0 (0)	0 (0)	1 (4)	0 (0)	1 (4)	1
Tumour pain	0 (0)	1 (4)	0 (0)	0 (0)	1 (4)	1

Data are n (%) unless otherwise specified. Worst grade is included in the table.
Abbreviations: SAE, serious adverse event.
[a]Safety-evaluable patients receiving at least one dose of LOAd703. Database lock December 14, 2023. [b]Total number of reported SAEs in the study was 17 of which 2 (12%; cytokine release syndrome n=1; pyrexia n=1) were attributed to LOAd703.

Supplementary Table 3: Significant alterations in gene expression between < median OS and > median OS in patients at baseline

Gene	Log_2 (fold change > median OS - < median OS)	p-value[a]
CD55	1,445	0,004824
PPIA	0,4828	0,007852
MAGEC1	2,963	0,008925
ITGB4	2,246	0,012418
TLR6	1,173	0,012753
EOMES	1,105	0,013565
CD34	0,6977	0,018951
HLA-DRB4	3,315	0,020194
MAGEC2	3,129	0,020331
ELANE	0,8015	0,022088
PASD1	1,89	0,023328
KIR_Activating_Subgroup_2	0,7693	0,023508
ZNF346	0,7057	0,025893
IFNA7	0,812	0,027319
ITGA2	1,158	0,031263
SYCP1	0,9962	0,032979
EPCAM	0,6793	0,03455
PMCH	0,8632	0,03942
IRF4	2,135	0,041004
IL24	2,593	0,046292
LILRA1	0,5684	0,047114
CLEC6A	1,189	0,049892
ITCH	-1,093	0,000353
NT5E	-2,815	0,001564
TRAF3	-0,9525	0,002429
IFNGR1	-0,8996	0,002779
CD164	-1,012	0,003454
CEBPB	-1,056	0,0062
POLR2A	-0,8325	0,007818
SH2B2	-0,9507	0,009688
REL	-0,6946	0,009695
JAK1	-0,5782	0,011424
TNFRSF10B	-1,208	0,011779
TANK	-0,6088	0,012937
ATG7	-0,8655	0,013354
ENG	-0,8032	0,01471
ITGAX	-1,371	0,016418
ANP32B	-0,9476	0,017338
CD99	-0,973	0,019659

FCF1	-0,6204	0,021391
FN1	-2,31	0,023204
ATG16L1	-0,4329	0,026547
NRP1	-1,387	0,030825
TFRC	-1,221	0,031994
HPRT1	-0,8732	0,033184
ATG5	-0,5308	0,03357
CYFIP2	-1,15	0,033931
CD47	-0,6263	0,033999
NFATC4	-1,155	0,03736
IL1R1	-1,508	0,037533
MAP3K7	-0,4841	0,039129
IKBKG	-0,3609	0,043734
SAP130	-0,5142	0,044176
BCL2L1	-0,5482	0,044514
ATF2	-0,5063	0,049692

[a]Unpaired, two-sided *t*-test. No correction for multiple comparison. OS: overall survival.

Supplementary Table 4: Significant alterations in gene expression between < median OS and > median OS in patients post-treatment (week 9)

Gene	Log_2 (fold change > median OS - < median OS)	p-value[a]
TMEFF2	1,033	0,009959
NCAM1	2,196	0,015045
ATF1	0,5941	0,016495
C1QBP	0,8322	0,02206
PSMB8	0,8914	0,024259
MAP2K4	0,6585	0,031566
TRIM39	0,9283	0,032048
GPI	0,6709	0,035432
CD1A	1,045	0,038212
ITGAX	-2,391	0,000522
NFATC4	-1,059	0,004079
NFKB2	-1,021	0,006618
ZNF346	-0,6314	0,009641
VEGFC	-1,056	0,019502
RUNX3	-1,019	0,022803
CT45A1	-1,564	0,029375
RUNX1	-1,03	0,031357
NCF4	-0,9928	0,03263
PLAUR	-1,448	0,032932
CXCL5	-1,581	0,033454
STAT6	-0,8147	0,044219
NEFL	-0,9997	0,046003
CSF3R	-1,392	0,046678
TNFRSF1B	-0,8172	0,048603

[a]Unpaired, two-sided *t*-test. No correction for multiple comparison.
OS: overall survival.

Supplementary Table 5: Significant alterations in protein expression between pre- and post-treatment (week 9) serum samples, Immune oncology panel

Protein	Log_2 (fold change week 9 – week 0)	p-value[a]
PD-L1	4,13	1,84E-13
Gal-9	0,249	0,002767
CXCL9	0,8352	0,003509
ADGRG1	0,6402	0,00425
IL18	0,3124	0,004882
MUC-16	0,4177	0,005095
ARG1	0,567	0,005866
TNF	0,3059	0,008685
CXCL11	0,504	0,009881
MMP12	0,5969	0,01025
CXCL10	0,6649	0,012896
CD8A	0,5706	0,014213
PDCD1	0,5469	0,014736
CD5	0,2644	0,016808
GZMA	0,487	0,017399
TNFRSF9	0,346	0,01855
IL12RB1	0,2087	0,019251
MIC-A/B	0,2873	0,020693
KLRD1	0,4694	0,021588
CRTAM	0,4925	0,023262
NCR1	0,2128	0,028827
IL8	0,4502	0,02889
FASLG	0,2507	0,030005
ADA	0,3128	0,031498
NOS3	0,2006	0,032604
CCL3	0,2798	0,033134
GZMH	0,9973	0,035957
CD27	0,2364	0,038086
IL10	0,3484	0,045484
CD83	0,1832	0,045845

[a]Paired, two-sided *t*-test. No correction for multiple comparison.

Supplementary Table 6: Significant alterations in protein expression between pre- and post-treatment (week 9) serum samples, Oncology panel

Protein	Log_2 (fold change week 9 – week 0)	p-value[a]
TNFRSF6B	0,3422	0,002956
ADAM 8	0,3262	0,003799
CD48	0,2816	0,004079
CD27	0,3057	0,004389
MIC-A/B	0,3159	0,005116
LY9	0,3642	0,005159
CXCL13	0,447	0,011495
TNFRSF4	0,3435	0,019458
MUC-16	0,2715	0,025268
FASLG	0,3038	0,027177
GZMH	1,095	0,029913
CD160	0,269	0,030383
EPHA2	0,3023	0,030954
TFPI-2	0,5667	0,034403
VIM	0,693	0,037757
VEGFR-3	0,1287	0,038049
MK	0,5104	0,039965
TXLNA	0,399	0,046828

[a]Paired, two-sided *t*-test. No correction for multiple comparison.

Supplementary Table 7: Best overall metabolic response

**Best Overall Metabolic Response**	LOAd703 $1x10^{11}$ VP + Atezolizumab *n=4*	LOAd703 $5x10^{11}$ VP + Atezolizumab *n=5*	LOAd703 (all) + Atezolizumab *n=9*
Complete Metabolic Response (CMR)	0 (0·0%)	0 (0·0%)	0 (0·0%)
Partial Metabolic Response (PMR)*	3 (75·0%)	3 (60·0%)	6 (66·7%)
Stable Metabolic Disease (SMD)	0 (0·0%)	0 (0·0%)	0 (0·0%)
Progressive Metabolic Disease (PMD)**	1 (25·0%)	2 (40·0%)	3 (33·3%)
Best Overall Metabolic Response (BOMR: CMR or PMR)	3 (75·0%)	3 (60·0%)	6 (66·7%)

Patients who underwent at least one post-dose FDG-PET/CT response assessment are included in the Table. Metabolic response was evaluated according to the European Organization for Research and Treatment of Cancer (EORTC) response evaluation criteria. *PMR was defined as a ≥15% decrease in maximum standardized uptake value (SUVmax). **MPD was defined as a ≥25% increase in SUVmax.

Supplementary Table 8. Cox Regression Analysis

Characteristics		Median (range)	Number of patients (%)	Survival (months), mean (range)	HR (95% CI)	*p*-value
Age (years)		61.5 (39-76)		18.9 (2.2-39.2)	1.00 (0.95-1.05)	0.97
Gender	Female		11 (45.8)	19.2 (3.9-38.6)		
	Male		13 (54.2)	18.6 (2.2-39.2)	0.89 (0.31-2.55)	0.83
Dose	Low		7 (29.2)	21.6 (2.2-39.2)		
	High		17 (70.8)	17.8 (2.8-36.7)	1.08 (0.33-3.47)	0.90
ECOG 0 at baseline	No		15 (62.5)	23.5 (2.3-39.2)		
	Yes		9 (37.5)	11.3 (2.2-36.7)	2.65 (0.91-7.74)	0.075
LDH	No		13 (54.2)	23.3 (3.9-39.2)		
(increased at baseline)	Yes		11 (45.8)	13.7 (2.2-36.7)	1.84 (0.64-5.31)	0.26
Prior radiation	No		13 (54.2)	19.1 (2.3-39.2)		
	Yes		11 (45.8)	18.7 (2.2-38.6)	0.93 (0.32-2.68)	0.89
BRAF inhibitor treatment	No		19 (79.2)	19.5 (2.3-38.6)		
	Yes		5 (20.8)	16.8 (2.2-39.2)	1.15 (0.32-4.15)	0.83
Prior systemic therapy	1		8 (33.3)	23.8 (2.8-38.6)		
	2		4 (16.7)	13.8 (4.0-33.0)	1.95 (0.43-8.74)	0.38
	3		8 (33.3)	23.7 (2.2-39.2)	0.43 (0.082-2.26)	0.32
	4		2 (8.3)	5.0 (2.3-7.7)	5.53 (0.90-33.96)	0.065
	5		2 (8.3)	4.5 (4.1-4.9)	5.27 (0.80-34.54)	0.083
M1 status	M1a		12 (50)	17.3 (2.8-38.6)		
(B and C are compared to A)	M1b		2 (8.3)	34.8 (33.0-36.7)	0.0-Inf	0.999
	M1c		10 (41.7)	17.6 (2.2-35.0)	0.42-2.05	0.74

ECOG: Eastern Cooperative Oncology Group, LDH: lactate dehydrogenase, HR: hazard ratio.
A two-sided Cox regression analysis was performed.

LOKON

A Phase I/II Trial Investigating LOAd703 in Combination with Atezolizumab in Malignant Melanoma

EudraCT: 2019-003300-12
NCT04123470
IND:016877

Sponsor Study ID: LOKON003

Version Nr: 6.1 2023-06-09

Sponsor: Lokon Pharma AB

Angelica Loskog CEO, PhD

Changes to the protocol

Version 6.1<br>Dated 2023-06-09	Substantial changes<br>• Definition for the End of study has been modified under section Definition and Terms and in the section 3.5 End of Study and section 13.4 Study Report<br>• The expected duration of the study has been updated to reflect change of the End of Study definition (synopsis, 3.4 Duration of Study, 5.1 Treatment overview)<br>• Contact details for the new Site Principal Investigator at Baylor College of Medicine has been updated.<br>Administrative changes<br>• In sections 2.5 and 9.4.5, an update of adverse events associated with atezolizumab was done to align with the latest IB atezolizumab<br>• In section 5.7.5 Administration of Atezolizumab, a reference to section 7.2.6 Vital Signs has been added.<br>• In section 7.5.1 Tumor size; PET assessment has been added.<br>• Contact details in section 1 General information have been updated.<br>• Minor editorial changes were made throughout the document to correct typographical errors and to improve consistency and clarity.
Version 6.0<br>Dated 2022-04-25	Substantial changes<br>• Number of patients needed to be enrolled to achieve at least 25 evaluable patients at MTD has been changed to up to 50 throughout the document (synopsis, section 3.3 Summary of Trial Design)<br>• The expected duration of the study has been updated (synopsis, section 3.5 End of Study, 5.0 Treatment of Patients).<br>• An additional site has been added (section 1.0 General Information)<br>• New exclusion criteria no. 32: Adenovirus-based vaccines (e.g Vaxzevria, known as COVID-19 vaccine Astra Zeneca, J&J Covid-19 vaccine) are prohibited 3 months prior to initiation of study treatment, during treatment and 6 months after the final dose of LOAd703 (synopsis, section 4.2, concomitant medication section 5.8).<br>• In section 5.8 Approved and Non-approved concomitant treatment it has been added that palliative surgery and local radiotherapy will be allowed.<br>• The time points for vital signs measurements have been updated in section 5.1 Treatment Overview and 7.2.6 Vital signs.<br>• Addition for US only, as already approved for Sweden. In section 5.1 Treatment Overview and 6.2 Screening, 7.2.10 Blood Chemistry and 7.2.11 Hematology it has been clarified

that “If samples are taken for routine analysis <7 days prior to screening, the results can be used for eligibility evaluation at the discretion of the Investigator, without need of subject the patients for new sampling”.

- Addition for Sweden only. In section 7.3.1 Anti-Adenoviral Antibodies addition of “**Valid for Swedish patients:** patients enrolled at Uppsala site, Sweden will be asked to provide additional blood samples for research purposes to identify and isolate B-cells producing anti-adenoviral antibodies. Blood samples will be collected at 2-3 occasions (up to 42 ml in total) during the LOAd703 treatment period (starting from week 6 until week 33)” have been made.

<u>Administrative changes</u>

- Unit for serum albumin levels was corrected from ≥ 2.5 mg/dL to ≥ 2.5 g/dL.
- Contact details for Biobank in Sweden and Research Analysis Laboratory has been updated.
- Information regarding previous studies in section 2.4 has been updated.
- In exclusion criteria no. 18, the reference to other exclusion criteria has been corrected.
- In section 4.4, criteria for off-treatment, survival follow-up and off-study patients has been clarified and corrected throughout the document.
- Clarification has been made in section 5.3.1, that Investigator may also perform intra-tumoral injections of LOAd703
- Clarifications have been made in section 5.3.2 GMO Regulation at Trial Site.
- Clarifications have been made in section 6.4, 7.2.7, 7.2.10, 7.2.11 and Appendix I, regarding Modified Follow-Up. In addition a schedule has been added as Appendix III.
- Minor corrections in Appendix I. Schedule of events: vital signs at week 33 were marked, updated caption 5, adding caption 9 and updated numbering
- Update to comply with the patient information and informed consent to allow request for biopsy from subsequent resections and to allow for samples to be analysed outside Sweden (section 7.1.1 and 7.5.2)
- In section 9.2 Evaluating and Documenting Adverse Events (AE) the procedure of AE reporting has been clarified if patients complete the study or are prematurely discontinued from the study.
- Section 9.4.3 Cytokine Release Syndrome (CRS) has been updated with clinical manifestations of CRS.
- Information regarding late reactions to atezolizumab in section 2.5 and 9.4.5 has been updated.

	• Virus units: 10e10 and 10e11 has been consequently changed to 10^{10} and 10^{11} respectively throughout the document.<br>• The week number for final follow-up visit has been added consequently throughout the document.<br>• Reference to atezolizumab where not relevant, has been removed throughout the document.<br>• Minor editorial changes were made throughout the document to correct typographical errors and to improve consistency and clarity.
**Changes made for Sweden only**<br>Version 5.1_SWE:<br>Dated 2021-02-15	Substantial change, section 7.2.10: Blood Chemistry and 7.2.11: Hematology:<br>• If samples are taken for routine analysis <7 days prior to screening, the results can be used for eligibility evaluation at the discretion of the investigator, without need of subject the patients for new sampling.
**Changes made for Sweden only**<br>Version 5.0_SWE:<br>Dated 2020-11-11	Substantial changes:<br><br>Synopsis and section 4.1 Inclusion criteria:<br>• **Valid for Swedish patients:** Patients not eligible for complete resection with locally advanced melanoma or metastatic melanoma can be included.<br>**Valid for US patients:** Patients with locally advanced melanoma or metastatic melanoma can be included, regardless of patient's eligibility for complete tumor resection.<br>• **Valid for Swedish patients:** Patients with B-Raf mutations must have received appropriate therapy with tyrosine kinase inhibitors(s) or MEK inhibitor (no changes from version 4.0)<br>**Valid for US patients:** Prior treatment with tyrosine kinase inhibitor(s) is optional; patients that have not yet received treatment with tyrosine kinase inhibitor(s) can be included in the study
Version 5.0:<br>Dated 2020-09-21	Substantial changes:<br><br>Synopsis and section 4.1 Inclusion criteria:<br>• A new criterion was added: A life expectancy of at least 3 months as per the investigator: this is a common inclusion criterion for this type of patients<br>• Patients with locally advanced melanoma or metastatic melanoma can be included, regardless of patient's eligibility for complete tumor resection.<br>• Prior treatment with tyrosine kinase inhibitor(s) is optional; patients that have not yet received treatment with tyrosine kinase inhibitor(s) can be included in the study<br>• Cut-point for serum albumin levels was changed to ≥ 2.5 mg/dL as well as requirement for AST and ALT was changed to ≤ 5 times the ULN if liver metastases are present<br>• Lactate dehydrogenase parameter was removed

	Synopsis and section 4.2 Exclusion criteria:<br>• Exclusion criteria no 1 was modified so that patients with mucosal melanoma will no longer be excluded.<br>• Exclusion of patients with progressive disease within 8 weeks after checkpoint inhibitor therapy and patients who have had more than 3 lines of treatment; were deemed to be to narrow and was replaced by a criterion excluding patients with rapid progression rate as assessed by the investigator<br>• Exclusion criterion describing number and site of metastases was updated: patients with central nervous system involvement (cerebral metastases) will be excluded, but not patients with bone metastases<br>• Washout period between cytotoxic and radiation therapy and protocol therapy (LOAd703/atezolizumab) was shortened to 14 days<br>• Washout period between immunostimulatory therapy and protocol therapy (LOAd703/atezolizumab) was shortened to 21 days<br>• Patients on warfarin continue to be excluded, but clarification was made that low molecular heparin is permitted<br><br>Administrative changes:<br>• Minor clarifications were done in the exclusion criteria no 6, 7, 8, 12, 14 and 17 (version 4.0).<br>• Synopsis: Extension of study duration: study duration was updated as patient recruitment to LOKON003 study was delayed due to COVID19 pandemic.<br>• Abbreviations: Correction of an abbreviation (DSUR)<br>• Section 1.0: Change in address (e-mail address and name of the mail system at Research Laboratory)<br>• Section 3.2: Correction of sample description in the secondary endpoints: update was made to comply with the rest of the protocol<br>• Section 3.6: Section update with the study timelines: update was made to comply with the synopsis<br>• Section 4.4: Clarification on the description of the Patient Withdrawal: update with relevant hyperlinks to protocol sections and clarification that patient continues with study schedule if withdrawal relates only to one study treatment.<br>• Section 4.4.1: Clarification on the Off-treatment patient section: update that description refers to both study treatment<br>• Section 4.5: Clarification on the description of the Patient Replacement: description refers to safety evaluation for phase I and DLT period, replacement of patients to meet efficacy evaluation is not required<br>• Section 5.1 and 5.2: Repaired hyperlinks referring to Appendix I<br>• Section 5.6.5: Clarification on start time point: LOAd703 is ready to use for 5 hours post preparation not thawing: update was made to comply with the rest of the protocol<br>• Section 7.2.10: Clarification on different types of blood clotting test performed at screening as part of the blood chemistry<br>• Section 7.4.1: Clarification on sampling schedule: sampling refers to patients staying overnight<br>• Section 9.2 and 9.2.1: Updated hyperlinks that referred to the NCI CTCAE Version 5.0.

	• Section 12.0: Update in numbering: section Protocol Modifications was moved under section Ethics therefore, numbering of this section was updated<br>• Section 13.3: Clarification on timelines for Record Keeping<br>• Section 13.4: Update on timelines for Study Report submission: to comply with the EU regulations<br>• Section 14.1 Appendix I: Schedule of Events: correction of the footnote numbers in the Table
Version 4.0:<br>Dated 2020-04-20	Substantial changes:<br>• Exclusion criteria 1 was modified so that patients with acral melanoma will not longer be excluded.<br>Administrative change:<br>• A clarification was made in section 2.6.1 so that it is clear that NSAID **or** steroid treatment can be used.
Version 3.0:<br>Dated 2020-03-26<br><br>*IND and EU*	Substantial changes:<br>• Exclusion criteria 15 was clarified to relate to monotherapy with a single PD-1/PD-L1 antibody.<br>Administrative changes:<br>• Section 1.4: The dept of Oncology in Uppsala has a new address.<br>• Section 5.6.3: Temporarily storage of LOAd 703 in -20°C for up to 3 months, has been removed.<br>• Section 5.6.5: the preparation instructions for LOAd703 has been clarified, to clearly state that the maximum dose, injected in 1 lesion, will not be diluted.<br>• Section 5.6.6 was clarified in regards to selection of lesion and that subcutaneous lesions, visible to the eye, may be photographed.<br>• Table I: Overview of ongoing clinical trials was updated.<br>• Section 9.4 Immunological AEs and Handling Plan has been updated to refer to the atezolizumab IB.<br>• Table 3 was removed, which affect the numbering of the 2 subsequent tables.
Version 2.0:<br>dated 2020-01-09 (IND)<br><br>dated 2019-12-02 (EU)<br>*Note: EU version 2.1, dated 2020-01-09, was issued to include administrative changes added to the submitted IND version 2.0 (commented in the list).*<br><br>*The content in IND version 2.0 is thus identical to the content in EU version 2.1.*	Substantial changes:<br>Secondary objectives and endpoints (sections synopsis, 3.1, 3.2)<br>• The languish is changed to better define what is being evaluated using the objective and endpoints stated.<br>Exclusion criteria (sections synopsis, 4.2:<br>• Criteria 6-12: it is clarified that registration is regarded when the first dose of LOAd703 and atezolizumab is given.<br>• Criteria 18: it is clarified that the contraceptive method must be regarded highly effective, and that abstinence from heterosexual intercourse is a choice of contraceptive method as well depending on the lifestyle of the subject.<br>• Criteria 19: it is clarified that men that has a partner of childbearing potential who refuse highly effective contraceptives are excluded.<br>• Criteria 24: it is added that patients with tested reduced functional respiratory capacity are excluded.<br>Dose limiting toxicity<br>• We added information about the DLT evaluation during dose escalation.

	• 5.6.1, 5.6.5: we removed the use of Ringer's acetate infusion solution so that all LOAd703 dilutions will be diluted in formulation buffer or physiological saline.<br>• 5.6.3, 5.6.5: we changed that an unopened vial should be used within 24 hours from thawing.<br><br><u>Administrative change:</u><br>• Treatment description (synopsis) is clarified with number of study weeks on atezolizumab.<br>• Study duration (synopsis): Total study months are clarified and maximum treatments weeks for each patients considering also the survival follow-up.<br>• Definition of terms are updated for end of study and withdrawal.<br>• General information (section 1.2): the Sponsor has changed address.<br>• Section 1.4: the site in North Carolina was replaced by a site in Los Angeles. *(in IND version 2.0 and in EU version 2.1)*<br>• Section 2.2.2, 2.3.2, 2.4, 2.5: when referred to an IB, the specific section is stated.<br>• Section 2.6.1: we have expanded the risk assessment with information of risks based on administration and patient selection as well as carcinogenicity. We also clarified risk of spreading and recombination.<br>• Section 2.6.3: we have expanded the risk assessment for the combined use of LOAd703 and atezolizumab in regard to age, gender, health status and carcinogenicity.<br>• Section 2.6.4, 2.7: we have expanded the potential benefits and the rationale of combining the two agents used in the study and the rationale for the doses.<br>• Section 3.4, 3.5: we have clarified the total study duration and maximum weeks of participation for each patient considering also the survival follow-up.<br>• Section 4.3: it has been clarified how the investigators and CRO communicates to recruit patients and when slots are available for patient registration in the trial.<br>• Section 4.4.1, 4.4.2, 4.4.3, 9.3.1: off-treatment and off-study patients have been clarified and off-visit patient removed to avoid confusion.<br>• Section 5.1: the survival follow-up has been clarified, and the information that CRO is distributing treatment slots to sites is added.<br>• Section 5.2: study week participation to reflect survival follow-up has been clarified.<br>• Section 5.6.10, 5.7.8: we clarified that study drugs are not available for patients post study.<br>• Section 7.2.12: information of contraceptive methods were added<br>• Section 7.5.3: survival follow-up has been clarified.<br>• Section 9.0, 9.2: it was clarified that both LOAd703 and atezolizumab are considered experimental drugs and that we will record AEs for both.<br>• 9.6: it was clarified that a female partner of child bearing potential to a male trial subject needs to be informed about the study and to inform the investigator if a pregnancy should occur.

	• Table 4 was expanded to include more patients (e.g. columns 21, 24 and 25) and a foot note was added to clarify that if the DLT level reach the elimination limit, this dose level is abandoned from the study independently of DLT events at lower dose levels (Section 10.2.1). *(in IND version 2.0 and in EU version 2.1)*<br>• In section 10.2.1 the DLT period has been adjusted to *3 weeks* after the second dose of LOAd703 and/or atezolizumab, to comply with the rest of the protocol. *(in IND version 2.0 and in EU version 2.1)*<br>• Section 11.3: it was clarified that the Sponsor is responsible that the trial is adequately monitored.<br>• Section 14.1: health status was removed from the table since its part of the medical history.

TABLE OF CONTENTS

INVESTIGATOR'S STATEMENT **13**

SYNOPSIS **14**

ABBREVIATIONS **18**

DEFINITION OF TERMS **21**

1.0 GENERAL INFORMATION **22**
- 1.1 PROTOCOL NUMBER AND TITLE OF THE STUDY 22
- 1.2 SPONSOR 22
- 1.3 CRO 22
- 1.4 INVESTIGATORS AND INSTITUTIONS 22
- 1.5 MANUFACTURERS 23
- 1.6 BIOBANK 23
- 1.7 LABORATORIES 24

2.0 BACKGROUND **25**
- 2.1 TUMOR IMMUNOLOGY AND CANCER IMMUNOTHERAPY 25
- 2.2 ONCOLYTIC VIRUS (OV) THERAPY 25
  - *2.2.1 Oncolytic Viruses* *25*
  - *2.2.2 The Investigational Product LOAd703* *25*
- 2.3 IMMUNE CHECKPOINT BLOCKADE THERAPY 27
  - *2.3.1 Checkpoint Blockade* *27*
  - *2.3.2 Atezolizumab* *27*
- 2.4 LOAD703 PREVIOUS CLINICAL STUDIES 27
- 2.5 ATEZOLIZUMAB – PREVIOUS CLINICAL STUDIES 29
- 2.6 POTENTIAL RISKS AND BENEFITS 29
  - *2.6.1 LOAd703 - Potential Risks and Action Plan* *29*
  - *2.6.2 Atezolizumab - Potential Risks and Action Plan* *36*
  - *2.6.3 Combination of LOAd703 and Atezolizumab - Potential Risks and Action Plan* *38*
  - *2.6.4 Potential Benefits – Rationale for Combining LOAd703 and Atezolizumab* *39*
- 2.7 RATIONALE FOR THE PHASE I/II DOSES 40
- 2.8 PATIENT POPULATION 40

3.0 TRIAL DESIGN **41**
- 3.1 OBJECTIVES 41
- 3.2 ENDPOINTS 41
- 3.3 SUMMARY OF TRIAL DESIGN 41
- 3.4 DURATION OF STUDY 42
- 3.5 END OF STUDY 42

4.0 SELECTION AND WITHDRAWAL OF PATIENTS **42**
- 4.1 INCLUSION CRITERIA 42
- 4.2 EXCLUSION CRITERIA 43
- 4.3 SCREENING, ENROLLMENT AND REGISTRATION LOG, IDENTIFICATION LIST AND NUMBERING OF SUBJECTS 45
- 4.4 WITHDRAWAL OF PATIENTS 46
  - *4.4.1 Off-treatment patient* *46*
  - *4.4.2 Survival follow-up patient* *47*
  - *4.4.3 Off-study patient* *47*
- 4.5 REPLACEMENT OF PATIENTS 47

4.6 NONCOMPLIANCE ... 47

5.0 TREATMENT OF PATIENTS ... 47

5.1 TREATMENT OVERVIEW ... 47
5.2 TREATMENT SCHEDULE FOR STUDY VISITS ... 49
5.3 STUDY SITES ... 50
5.3.1 General Description ... 50
5.3.2 GMO Regulation at Trial Site ... 50
5.4 REFERRALS FROM OTHER HOSPITALS ... 50
5.5 DOSE LIMITING TOXICITY (DLT) ... 50
5.6 LOAD703 DRUG PRODUCT ... 51
5.6.1 LOAd703 Brief Description ... 51
5.6.2 LOAd703 Packaging and Labeling ... 52
5.6.3 LOAd703 Storage and Handling ... 52
5.6.4 LOAd703 Accidents ... 52
5.6.5 Preparation of LOAd703 Prior to Treatment ... 53
5.6.6 Administration of LOAd703 ... 53
5.6.7 LOAd703 Unused Clinical Trial Supplies ... 54
5.6.8 LOAd703 Dose Modifications ... 55
5.6.9 LOAd703 Maximum Tolerated Dose (MTD) ... 55
5.6.10 LOAd703 Continuation of Treatment Post Study ... 55
5.7 CHECKPOINT BLOCKADE THERAPY WITH TECENTRIQ®/ATEZOLIZUMAB ... 55
5.7.1 Atezolizumab Brief Description ... 55
5.7.2 Atezolizumab Packaging and Labeling ... 56
5.7.3 Atezolizumab Storage and Handling ... 56
5.7.4 Preparation of Atezolizumab Prior to Treatment ... 56
5.7.5 Administration of Atezolizumab ... 57
5.7.6 Atezolizumab Unused Clinical Trial Supplies ... 57
5.7.7 Atezolizumab Dose Modifications ... 58
5.7.8 Continuation of Atezolizumab Treatment Post Study ... 58
5.8 APPROVED AND NON-APPROVED CONCOMITANT TREATMENT ... 58
5.9 MONITORING SUBJECT COMPLIANCE ... 58

6.0 STUDY EVALUATIONS ... 58

6.1 SCHEDULE OF EVENTS ... 59
6.2 SCREENING ... 59
6.3 TREATMENT STUDY VISITS ... 59
6.4 EVALUATION, MODIFIED FOLLOW-UP AND FINAL FOLLOW-UP VISIT ... 59

7.0 STUDY ASSESSMENTS ... 60

7.1 COLLECTION OF BLOOD AND TISSUE SAMPLES AND STORAGE ... 60
7.1.1 Blood Samples, Biopsy and Shedding ... 60
7.1.2 Storing Samples in Biobank ... 61
7.2 SAFETY PARAMETERS ... 61
7.2.1 Demographics ... 61
7.2.2 Body Measurements ... 61
7.2.3 Medical History/Patient History ... 61
7.2.4 Physical Exam ... 61
7.2.5 Continuation Criteria ... 62
7.2.6 Vital Signs ... 62
7.2.7 ECOG Performance Status ... 62
7.2.8 Pregnancy Test ... 63
7.2.9 12-Lead ECG ... 63

7.2.10 Blood Chemistry 63
7.2.11 Hematology 63
7.2.12 Concomitant Medications 63
7.2.13 Adverse Events (AE) Monitoring 64
7.3 IMMUNOLOGICAL ASSESSMENTS 64
7.3.1 Anti-Adenoviral Antibodies 64
7.3.2 Anti-Drug Antibodies (ADA) 65
7.3.3 Immune Cell Phenotyping 65
7.3.4 Cytokines and Other Protein Responses 65
7.3.5 Antigen-Specific T Cells 65
7.4 PHARMACOKINETICS AND VIRUS SHEDDING 65
7.4.1 Pharmacokinetics in Blood 65
7.4.2 Presence of LOAd703 in Tumor Lesions 66
7.4.3 Analysis of LOAd703 Shedding in Oral and Rectal Swabs, and Urine 66
7.5 EFFICACY ASSESSMENTS 66
7.5.1 Tumor Size 66
7.5.2 Tumor Markers 67
7.5.3 Survival Follow-Up 67

8.0 RESPONSE CRITERIA 67
8.1 RECIST 67
8.2 OS, TTP AND PFS 68

9.0 ADVERSE EVENTS (AE) 68
9.1 DEFINITIONS 68
9.1.1 Adverse Event (AE) 68
9.1.2 Serious Adverse Event (SAE) 68
9.1.3 Suspected Unexpected Serious Adverse Reactions (SUSAR) 69
9.1.4 Serious Adverse Reactions (SAR) 69
9.1.5 Non-Serious Adverse Event 69
9.2 EVALUATING AND DOCUMENTING ADVERSE EVENTS (AE) 69
9.2.1 Severity Grading 70
9.2.2 Attribution Definitions 70
9.2.3 Duration of Event 70
9.2.4 Action(s) Taken Regarding the Study Drugs 71
9.3 REPORTING SERIOUS ADVERSE EVENTS (SAE), DEATHS, UNEXPECTED AES AND DLTS 71
9.3.1 Reporting to Sponsor 71
9.3.2 Safety Report – Reporting by Sponsor 71
9.3.3 Reporting to IEC/IRB 72
9.3.4 Procedures in Case of Medical Emergency 72
9.4 IMMUNOLOGICAL AES AND HANDLING PLAN 72
9.4.1 Immediate Reaction to the LOAd703 Virus Particle 72
9.4.2 Late Reactions Due to LOAd703 Transgene Expression 73
9.4.3 Cytokine Release Syndrome (CRS) 73
9.4.4 Immediate - Related Reactions to Atezolizumab 73
9.4.5 Late Reactions to Atezolizumab 74
9.5 HANDLING OF PREGNANCY 74

10.0 STATISTICS 74
10.1 STATISTICAL ANALYSIS PLAN 74
10.2 SAMPLE SIZE 75
10.2.1 Safety 75
10.2.2 Effect 75

10.3 Safety Reporting 76
10.4 Efficacy Reporting 76
10.5 Immunological Parameters 76
10.6 Pharmacokinetics and Shedding 77
10.6.1 Pharmacokinetics 77
10.6.2 LOAd703 Shedding 77

11.0 QUALITY CONTROL AND QUALITY ASSURANCE 77
11.1 Direct Access to Source Data/Documents 77
11.2 Source Data 77
11.3 Monitoring 78

12.0 ETHICS 78
12.1 Protocol Modifications 78
12.2 Independent Ethics Committee (IEC)/Institutional Review Board (IRB) 79
12.3 Ethical Conduct of the Study 79
12.4 Patient Information and Informed Consent 79

13.0 DATA MANAGEMENT 80
13.1 Data Management 80
13.1.1 Data Entry and Data Validation 80
13.1.2 Database Closure 80
13.2 Electronic Case Report Forms (eCRFs) 80
13.3 Record Keeping 80
13.4 Study Report 81
13.5 Publication Policy 81
13.6 Insurance 81

14.0 APPENDICES 81
14.1 Appendix I: Schedule of Events 82
14.2 Appendix II: ECOG Performance Status 84
14.3 Appendix III: Modified Follow-Up Schedule 85

15.0 REFERENCES 86

INVESTIGATOR'S STATEMENT

1. I have carefully read this protocol entitled "A Phase I/II Trial Investigating LOAd703 in Combination with Atezolizumab in Malignant Melanoma" and agree that it contains all the necessary information required to conduct the study. I agree to conduct this study as outlined in the protocol.

2. I understand that this study will not be initiated without approval of the appropriate Institutional Review Committee/Independent Ethics Committee (IRB/IEC), and that all administrative requirements of the governing body of the Institution will be complied with fully.

3. Informed written consent will be obtained from all participating patients in accordance with institutional guidelines, FDA requirements as specified in Title 21 CFR, Part 50, the European Union Directive 2001/20/EC and its associated Detailed Guidance's, European Union GAP Directive 2005/28/EC, the ICH Guideline for Good Clinical Practice, Section 4.8, and the terms of the Declaration of Helsinki (2013) depending on the country of patient enrollment.

4. I will enroll patients who meet the protocol criteria for entry.

5. I understand that my signature on each completed Case Report Form indicates that I have carefully reviewed each page and accept full responsibility for the contents thereof.

6. I understand that the information presented in this study protocol is confidential, and I hereby assure that no information based on the conduct of the study will be released without prior consent from the Sponsor unless this requirement is superseded by the Food and Drug Administration, a Competent Authority of the European Union or another Regulatory Authority.

Investigator:

Name: GUSTAV ULLENHAG Telephone: [redacted]

Institution: Dpt of oncology, U:a university hospital

Signature: Gustav Ullenhag Date: 15/6 2023

SYNOPSIS

Title: A Phase I/II Trial Investigating LOAd703 in Combination with Atezolizumab in Malignant Melanoma

Investigational Product: LOAd703 (delolimogene mupadenorepvec) is an oncolytic adenovirus serotype 5/35 expressing human trimerized CD40L and full-length 4-1BBL under a CMV promoter. Atezolizumab is a humanized IgG1 monoclonal antibody that binds to PD-L1.

Study Design: Single arm, open-label, multicenter trial.

Patients will receive up to 12 LOAd703 intratumoral treatments in combination with intravenous infusions of atezolizumab. LOAd703 will be tested at two dose levels to determine the maximum tolerated dose (MTD) of LOAd703 evaluated in the study using a BOIN design. The LOAd703 dose can be divided for intratumoral injection into as many as 3 tumor lesions. Atezolizumab will be tested at a fixed dose.

At least 25 response evaluable patients will be enrolled at the MTD for evaluation of their response using binominal testing. The total number of patients to be enrolled in the study to achieve at least 25 evaluable patients at MTD, should not exceed 50.

Objectives: The primary objective is to determine the tolerability of LOAd703 administered by intratumoral injection(s) in combination with intravenous atezolizumab.

The secondary objectives are to determine the antitumor activity as well as the pharmacokinetics and biological mechanisms-of-action of LOAd703 in combination with atezolizumab.

Endpoints:

Primary Endpoints

1. The primary endpoint is safety, determined by the National Cancer Institute Common Toxicity Criteria for Adverse Events (NCI CTCAE v5.0).

Secondary Endpoints:

1. Overall response rate evaluated by the Response Evaluation Criteria in Solid Tumors (RECIST), v1.1.
2. Shedding determined as levels of LOAd703 in shedding samples at different time points post-treatment.
3. LOAd703 leakage to blood determined as levels of LOAd703 in blood at different time points post-treatment.
4. Anti-adenovirus immunity determined as anti-viral antibodies and T cells after combination therapy compared to baseline.
5. Pharmacokinetics of atezolizumab determined as the level of atezolizumab in blood at different time points post-treatment.

6. Immunity to atezolizumab determined as the level of anti-drug antibodies (ADA) targeting atezolizumab at different time points post-treatment compared to baseline.
7. Immune profile as determined by changes in immune cell populations and their activation markers in tumor tissue and blood after combination therapy compared to baseline.
8. Protein profile as determined by changes in protein profile in blood after combination therapy compared to baseline.

Eligibility:

Inclusion Criteria

1. Pathological confirmation of melanoma.
2. A life expectancy of at least 3 months as per the investigator
3. **Valid for Swedish patients:** Patients has locally advanced melanoma or metastatic melanoma, but not eligible for complete resection of melanoma
   Valid for US patients: Patients has locally advanced melanoma or metastatic melanoma.
4. The patient has measurable disease (e.g., measurable tumor lesions must be present that can accurately be measured in at least one dimension with a minimum size of 10 mm by CT scan and MRI, 10 mm caliper measurement by clinical exam (when superficial), and/or 20 mm by chest X-ray).
5. Patient has at least one injectable tumor lesion that has not been irradiated or has been irradiated but disease progression documented at the site subsequent to radiation therapy.
6. The patient has received appropriate treatment with an anti-PD-1 or anti-PD-L1 antibody with or without an anti-CTLA4.
7. **Valid for Swedish patients:** Patients whose advanced melanoma has a B-Raf mutation must have received appropriate therapy with tyrosine kinase inhibitor(s) and/or MEK inhibitor
   Valid for US patients: Patients whose advanced melanoma has a B-Raf mutation may have received appropriate therapy with tyrosine kinase inhibitor(s) and/or MEK inhibitor as assessed by the investigator.
8. Age ≥ 18 years.
9. Eastern Cooperative Oncology Group (ECOG) performance status of 0 to 1.
10. Serum albumin ≥ 2.5 g/dL.
11. Absolute neutrophil count (ANC) ≥ 1.0 x 10e9/L.
12. Platelet count ≥ 100 x 10e9/L.
13. Prothrombin (INR) ≤ 1.5 or prothrombin time (PT) ≤ 1.5 times ULN; and either partial thromboplastin time or activated partial thromboplastin time (PTT or aPTT) ≤ 1.5 times the ULN.
14. Bilirubin < 1.5 times the institutional upper limit of normal (ULN).
15. Aspartate aminotransferase (AST) and alanine aminotransferase (ALT) ≤ 2.5 (≤ 5 if liver metastases are present) times the institutional ULN.
16. The patient must have signed informed consent.

Exclusion Criteria

1. Malignant melanoma that is uveal.
2. Subjects considered by the investigator to have rapid clinical progression due to melanoma
3. Subjects must not have greater than 3 cerebral melanoma metastases, and/or clinically active cerebral melanoma metastases, and/or a requirement for corticosteroid therapy, and/or carcinomatous meningitis regardless of clinical stability.
4. Any concurrent treatment that would interfere with the effect mechanisms of atezolizumab and LOAd703, including, but not limited to, continuous high-dose corticosteroids (>10 mg per day), lymphodepleting antibodies, or cytotoxic agents.
5. Treatment with inhibitors of immune function, such as lymphotoxic monoclonal antibodies (e.g., alemtuzumab), or rapamycin/rapamycin analogs, or cytotoxic agents within 21 days of the first dose of LOAd703/atezolizumab.
6. Therapeutic treatment with systemic antibiotics within 14 days of the first dose of LOAd703/atezolizumab.
7. Treatment with biologic therapy within 21 days of the first dose of LOAd703/atezolizumab.
8. Treatment with cytotoxic anticancer therapy within 14 days of the first dose of LOAd703/atezolizumab.
9. Treatment with wide-field radiation within 14 days of the first dose of LOAd703/atezolizumab.
10. Prior treatment with an adenovirus-based gene therapy.
11. Use of any investigational agents within 21 days of the first dose of LOAd703/atezolizumab.
12. The use of systemic immunostimulatory agents (including, but not limited to, interferons and IL2) are prohibited within 21 days or 5 half-lives (whichever is longer) of the first dose of LOAd703/atezolizumab.
13. Failed resolution/improvement of AEs including those related to anti-PD-1/anti-PD-L1 to grade 0-1 and requirement for treatment with ≥10 mg/day prednisone (or equivalent) for at least two weeks prior to registration.
14. History of CTCAE grade 4 immune-related AEs from monotherapy using an anti-PD-1/anti-PD-L1 antibody.
15. History of CTCAE grade 4 AE that require steroid treatment (>10 mg/day prednisone or equivalent) for >12 weeks.
16. Patients requiring warfarin are not eligible (low molecular weight heparin is permitted).
17. Women who are pregnant (as confirmed by pregnancy test during screening in applicable patients), breastfeeding, or planning to become pregnant during the study period, or women of childbearing potential who are not using acceptable highly effective contraceptive methods. A woman is considered of childbearing potential if she is not surgically sterile or is less than 1 year since her last menstrual period. The following are acceptable as highly effective contraceptive methods: combined (estrogen- and progesterone-containing) hormonal contraception associated with inhibition of ovulation (oral, intravaginal, transdermal), progesterone-only hormonal contraception associated with inhibition of ovulation (oral, injectable, implantable), intrauterine device, intrauterine hormone-releasing system, bilateral tubal occlusion and vasectomized partner or abstinence of heterosexual intercourse during the entire study period (depending on the preferred and usual life style of the subject).
18. Men who do not consent to the use of condoms during intercourse during study participation or has a partner of childbearing potential, who will not use any of the highly effective contraceptive methods exemplified in exclusion criteria no 17.

19. Known active hepatitis B or C infection, or HIV infection.
20. Patients with active, severe autoimmune disease or immune deficiency or previous Guillain-Barré syndrome. Patients with eczema, psoriasis, lichen simplex chronicus or vitiligo with dermatologic manifestations only (e.g., patients with psoriatic arthritis are excluded) are eligible for the study provided all of following conditions are met:
    a. Rash must cover <10% of body surface area.
    b. Disease is well-controlled at baseline and requires only low-potency topical corticosteroids.
    c. Occurrence of acute exacerbations of the underlying condition requiring psoralen plus ultraviolet A radiation, methotrexate, retinoids, biologic agents, oral calcineurin inhibitors, or high-potency or oral corticosteroids within the previous 12 months.
21. History of leptomeningeal disease.
22. Uncontrolled pleural effusion, pericardial effusion, or ascites requiring recurrent drainage procedures (once monthly or more frequently).
23. History of idiopathic pulmonary fibrosis, organizing pneumonia (e.g., bronchiolitis obliterans), drug-induced pneumonitis or idiopathic pneumonitis, or evidence of active pneumonitis on screening chest computed tomography (CT) scan or tested reduced functional respiration capacity. However, history of radiation pneumonitis in the radiation field (fibrosis) is permitted.
24. Unstable angina, uncontrolled cardiac arrhythmia, recent (within 3 months) history of myocardial infarction or stroke, or New York Class III/IV congestive heart failure.
25. Major surgical procedure other than for the malignant melanoma diagnosis, within 4 weeks prior to initiation of the study treatment, or anticipation of the need for a major surgical procedure during the study.
26. Prior allogeneic stem cell or solid organ transplantation.
27. History of severe allergic anaphylactic reactions to chimeric human or humanized antibodies, or fusion proteins.
28. Known hypersensitivity to CHO cell products or any component of the atezolizumab formulation.
29. Uncontrolled intercurrent illness including, but not limited to, psychiatric illness/social situations that in the opinion of the Investigator would compromise compliance to study requirements or put the patient at unacceptable risk.
30. Other malignancy within the past 2 years (not including basal cell or squamous cell carcinoma of the skin, prostate cancer without the need of other treatment than hormones or *in situ* cervix, breast or melanoma).
31. Live, attenuated vaccines (e.g., FluMist®) are prohibited within 4 weeks prior to initiation of study treatment, during treatment, and for 5 months after the final dose of atezolizumab and/or LOAd703.
32. Adenovirus-based vaccines (e.g., Vaxzevria, known as COVID-19 vaccine Astra Zeneca, J&J Covid-19 vaccine) are prohibited 3 months prior to initiation of study treatment, during treatment and 6 months after the final dose of LOAd703.

Treatment Description: Two dose levels of LOAd703 (total viral load: $1x10^{11}$ and $5x10^{11}$ VP) will be tested in combination with a fixed dose of atezolizumab (1200 mg). Treatments of LOAd703 (up to 12) will be delivered by image-guided intratumoral injection concurrent with intravenous atezolizumab treatment (1200 mg), where both treatments will be given

	every 3 weeks. The total LOAd703 dose can be divided among 1-3 tumor lesions. Patients will continue atezolizumab treatment up to study week 54 unless tumor progression or unacceptable toxicity is observed. Radiological imaging as well as blood and biopsy sampling will be performed to monitor safety, mechanisms-of-action and disease status. Oral and rectal swabs, and urine samples will be collected from all patients to analyze virus shedding.
**Accrual Objective:**	Up to 50 patients
**Study Duration:**	The trial encompasses a treatment phase ending with last patient last visit (LPLV) (FPI: Q3 2020; LPLV:Q1 2024; study end: Q1 2024). For each patient, active participation with study visits is maximum 60 weeks where after they are followed for survival as long as the trial remains open. Hence, maximum trial duration for a patient is 48 months.

ABBREVIATIONS

aCTLA4	Anti-cytotoxic T lymphocyte antigen 4
ACS	American Cancer Society
Ad	Adenovirus
ADA	Anti-drug antibodies
AE	Adverse event
AICD	Activation-induced cell death
ALT	Alanine aminotransferase
ANC	Absolute neutrophil count
aPTT	Activated partial thromboplastin time
ASCO	American Society of Clinical Oncology
AST	Aspartate aminotransferase
ATMP	Advanced therapy medicinal product
AUC	Area under curve
BOIN	Bayesian Optimal Interval
CAGT	Center for Cell and Gene Therapy
CBR	Clinical benefit rate
CD	Cluster of differentiation
CDC	Center for Disease Control
CD40L	CD40 ligand, CD154
CEA	Carcinoembryonic antigen
CHO	Chinese hamster ovary cells
CI	Confidence interval
CFR	Code of Federal Regulation
CMV	Cytomegalovirus
CR	Complete response
CRO	Contract research organization
CRP	C reactive protein
CRS	Cytokine release syndrome

CT	Computer tomography
CTCAE	Common Terminology Criteria for Adverse Events
CTL	Cytotoxic T lymphocyte
CV	Curriculum vitae
DC	Dendritic cell
DCF	Data clarification form
DLT	Dose limiting toxicity
DMP	Data management plan
DSUR	Development safety update report
ECG	Electrocardiogram
ECOG	Eastern cooperative oncology group
eCRF/CRF	Electronic case report form
EDC	Electronic data capture
EGFR	Epidermal growth factor receptor
ELISA	Enzyme-linked immunosorbent assay
EMA	European Medicines Agency
EU	European Union
FASS	Swedish online reference of approved drug products
FDA	Food and Drug Administration
FPI	First patient in
FU	Follow-up
GCP	Good clinical practice
GDPR	General data protection regulation
GM-CSF	Granulocyte macrophage-colony stimulating factor
GMO	Genetically modified organism
HUVEC	Human umbilical vein endothelial cell
IA	Immunology assessment
IB	Investigator’s Brochure
IEC	International ethics committee
IFNg	Interferon gamma
IL	Interleukin
IND	Investigational new drug
INR	International normalized ratio
IRB	Institutional review board
IRR	Immune-related reaction
i.t.	Intratumoral
IU	Infectious units
IV	Intravenous
LFF/LÖF	Swedish patient insurance
LOAd	Lokon oncolytic adenovirus
LPLV	Last patient last visit
LOAd703	LOAd virus containing transgenes CD40L and 4-1BBL
MedDRA	Medical Dictionary for Drug Regulatory Activities
MDSC	Myeloid-derived suppressor cell

MHC	Major histocompatibility complex
mLOAd703	LOAd703 virus containing murine transgenes (CD40L and 4-1BBL)
MPA	Medical products agency
MR	Mixed response
MRI	Magnetic resonance imaging
MSI	Microsatellite instability
MTD	Maximum tolerated dose
MTS	Tetrazolium compound used in cell metabolic assay
NCI CTCAE	National Cancer Institute Common Toxicity Criteria for Adverse Events
NK	Natural killer
NSAID	Nonsteroidal anti-inflammatory drug
ORR	Overall response rate
OS	Overall survival
OV	Oncolytic virus
PCR	Polymerase chain reaction
PD	Progressive disease
PDAC	Pancreatic ductal adenocarcinoma
PD-1	Programmed death receptor 1
PD-L1	Programmed death receptor ligand 1
PET	Positron emission tomography
PFS	Progression-free survival
PR	Partial response
PT	Prothrombin time
PTT	Partial thromboplastin time
q3w or q4w	Every third week or every fourth week
Rb	Retinoblastoma
RCA	Replication-competent adenovirus
RECIST	Response evaluation criteria in solid tumors
RSI	Reference safety information
SAE	Serious adverse event
SAP	Statistical analysis plan
SAR	Suspected adverse reaction
SD	Stable disease
SmPC	Summary of Product Characteristics
SoC	Standard of care
SOP	Standard operating procedure
SUSAR	Suspected unexpected serious adverse reactions
TCR	T cell receptor
TGFb	Transforming growth factor beta
Th	T helper
TLR	Toll-like receptor
TMZ-CD40L	Trimerized membrane-bound CD40L
TNFa	Tumor necrosis factor alpha
Treg	T regulatory cell

TTP	Time-to-tumor progression
UCR	Uppsala Clinical Research Center
ULN	Upper limit of normal
VEGF	Vascular endothelial growth factor
VP	Virus particles
WBC	White blood cells
4-1BBL	4-1BB ligand, CD137 ligand

DEFINITION OF TERMS

Baseline	Assessment/value before the 1st dose of LOAd703/atezolizumab.
DLT period	DLT evaluation for each patient is done when at least two doses of LOAd703/atezolizumab has been administered plus 3 weeks evaluation time after the second dose.
End of treatment	Last Patient's Last Visit (LPLV)
End of study	Last Patient's Last Visit (LPLV)
Enrolled patient	A patient who has signed informed consent and the screening visit can be initiated.
Evaluable patient	A patient that has received one dose of LOAd703/atezolizumab is evaluable for toxicity.<br>A patient that has received at least three doses of LOAd703/ atezolizumab and has available tumor assessment data is evaluable for efficacy.
Registered patient	An enrolled patient who has fulfilled the eligibility criteria after the screening visit has been performed and given a treatment slot in the study.
Study month	One study month = 4 weeks
Screen failure	Patient withdrawn before first LOAd703 dose.
Off-treatment	A patient that is discontinued from treatment, but will return for the clinical follow-up visits (modified schedule) and survival follow-up.
Survival follow up	A patient that is followed for survival.
Off-study	A patient that withdraws consent, is lost to follow up or study is terminated. No more information is collected for the patient i.e. the date for last data capture for the patient.

1.0 GENERAL INFORMATION

1.1 Protocol Number and Title of the Study

Sponsor Protocol Number: LOKON003

Title: A Phase I/II Trial Investigating LOAd703 in Combination with Atezolizumab in Malignant Melanoma

1.2 Sponsor

Lokon Pharma AB
Bredgränd 14
753 20 Uppsala, Sweden

Chief Executive Officer
Contact: Angelica Loskog
Email: angelica.loskog@lokonpharma.com
Phone: [redacted]

Sponsor Medical Advisor
Contact: Eric Rowinsky
Email: [redacted]
Phone: [redacted]

Clinical Trial Manager
[redacted]

1.3 CRO

Precision for Medicine, Oncology and Rare Diseases
200 Raritan Commons, North, Suite 102
Flemington, NJ 08822
United States
[redacted]

1.4 Investigators and Institutions

The dose escalation part of the study is planned to be conducted at 2 sites in US and 1 site in Sweden. For the dose expansion part of the study, additional clinics may be added in the US and Sweden. An updated contact list will always be available in the Investigator Study File.

Baylor College of Medicine
Baylor St. Luke's Medical Center
6720 Bertner
Houston, Texas 77030
United States

Site Principal Investigator: Meera Patel
Email [redacted]

Cedars-Sinai Medical Center,
The Angeles Clinic and Research Institute
A Cedars Sinai Affiliate
11800 Wilshire Blvd, Suite 300
Los Angeles, CA 90025
United States

Site Principal Investigator: Omid Hamid
Email: [redacted]

Uppsala University Hospital
Department of Oncology
Uppsala University Hospital, Entrance 100/101
751 85 Uppsala, Sweden

Site Principal Investigator: Gustav Ullenhag
Email: [redacted]

[redacted]

[redacted]

1.5 Manufacturers

LOAd703 Manufacturer
Center for Cell and Gene Therapy (CAGT)
Baylor College of Medicine
1102 Bates Ave, Ste. 1630
Houston, TX, USA 77030

Atezolizumab Manufacturer
F. Hoffmann-La Roche Ltd
Basel, Switzerland

1.6 Biobank

Uppsala Biobank (nr 827)
Dag Hammarskjöldsväg 38, 5tr UCR,
Science Park, Hubben
751 85 Uppsala, Sweden
info@uppsalabiobank.uu.se

1.7 Laboratories

Clinical Analyses
All study sites will use their local laboratory for clinical chemistry and hematology analyses.

Research Analysis Laboratory
Uppsala University
Department of Immunology, Genetics and Pathology
Rudbeck Laboratory C11, 2nd floor
Dag Hammarskjöldsväg 20
751 85 Uppsala, Sweden

Contact: Mariela Meija Cordova
[redacted]

2.0 BACKGROUND

2.1 Tumor Immunology and Cancer Immunotherapy

The immune system can recognize and kill tumor cells using the same mechanisms as it recognizes and kills virally infected cells to save the host against lethal infections. Like virally infected cells, tumor cells are self-cells, and viral- or tumor-associated epitopes are presented to CD8+ cytotoxic T lymphocytes (CTLs) via major histocompatibility complex I (MHC-I) on the cells. Both virally infected cells and tumor cells may prevent CTL recognition by downregulating MHC molecules, making the cells targets for natural killer (NK) cells. While viruses initially activate an anti-viral immune response by alerting antigen-presenting cells such as dendritic cells (DCs) to activate lymphocytes, tumor cells and its stroma tend to produce substances that inhibit immune activation and tumor immunity. In order to evade the immune system, tumors inhibit DC maturation and promote the differentiation and attraction of immunosuppressive (type 2) cells to the tumor milieu such as myeloid-derived suppressor cells (MDSCs) and T regulatory cells (Tregs). These immune regulating cells produce suppressive cytokines and growth factors that suppress activated CTLs in the tumor mileu and lead to T cell anergy (unresponsiveness) or even death.[1,2]

The principal goal of cancer immunotherapy is to break tumor tolerance (e.g., break anergy) and revert the ongoing type 2 immune responses to type 1. Type 1 is characterized by activation of T helper 1 (Th1) lymphocytes, CTLs, NK cells and M1 macrophages as well as by a cytokine pool that includes IFNγ, IL12, IL21 and TNFa.[3]

The implementation of checkpoint blockade antibodies targeting CTLA4 and PD-1/PD-L1 for various solid malignancies, as well as chimeric antigen receptor (CAR) T cells for B cell malignancies, has made immunotherapy a cornerstone in cancer management.[4,5] Nevertheless, most patients with solid malignancies do not respond to checkpoint blockade therapy or become resistant. Novel concepts to treat cancer by stimulating the immune system are currently being investigated that may be used alone, or in combination with checkpoint antibodies, such as immunostimulatory gene therapy utilizing oncolytic viruses as gene delivery vehicles.[6]

2.2 Oncolytic Virus (OV) Therapy

2.2.1 Oncolytic Viruses

The ability of certain viruses to infect cells, propagate and kill them by lysis during the release of new virions means that they can be utilized as cancer therapeutics. To limit oncolysis to tumor cells, the expression of viral replication genes is restricted by adding promoters that are preferentially active in the tumor.[6,7,8] For maximum potency, the OVs should infect all tumor cells. However, this is challenging if the tumor has metastasized, since systemic spreading of the virus to distal tumor may be limited by the immune system. Nevertheless, by arming the OVs with immunostimulatory genes (transgenes), it is possible to inject one or a few lesions to induce a systemic anti-tumor response whereby activated tumor-specific T cells can circulate and reach all tumor lesions. There are many different OVs being evaluated both preclinically and clinically. Most of the clinically advanced OVs encode GM-CSF such as the FDA- and EMA-approved OV Imylgic®.[9] Other options such as LOAd703 described herein encodes one or more immune stimulators. Imylgic® and other OVs are currently being combined with checkpoint blockade antibodies in clinical trials.

2.2.2 The Investigational Product LOAd703

LOAd703 is a novel immunotherapy for cancer. It is an oncolytic adenovirus serotype 5 with a fiber (shaft and knob) from serotype 35 (Ad5/35) to increase cell binding and infectivity of cells expressing human CD46. Virus replication and oncolysis is restricted to cells with a dysfunctional retinoblastoma (Rb) pathway due to an E1AΔ24 and multiple E2F-binding domains upstream of

E1A.[8] Rb in normal cells is bound to the transcription factor E2F which blocks its intrinsic capacity to induce transcription of genes that promote transition from G1 into S phase of the cell cycle. When Rb is phosphorylated, E2F is released and stimulates cell proliferation.[10] Human tumors have a wide spectrum of mutations that alter the Rb protein and/or factors that lead to hyper-phosphorylation of Rb. Hence, in cancer cells E2F is free to drive transcription of the virus. The virus infects and kills tumor cells via oncolysis due to excessive virus replication while healthy non-malignant cells can be infected but new virus particles are normally not produced. Therefore, LOAd703 does not kill, and is therefore not toxic to healthy cells. LOAd703 has a transgene cassette with two immunostimulatory genes (TMZ-CD40L and 4-1BBL) driven by a CMV promoter. The CMV promoter is not tissue-restricted and the immunostimulatory genes can be expressed in all cells that are infected by LOAd703, independent of virus replication. Thus, LOAd703 targets both the tumor and its stroma to induce transgene expression but virus-mediated oncolysis will only occur in tumor cells. Since the virus is administered by intratumoral (i.t.) injection, the expression of the transgenes is localized to the tumor area.

CD40L is a potent stimulator of myeloid cells and an inducer of Th1 type immune responses.[11] The TMZ-CD40L transgene is a specially designed human CD40 ligand (CD40L; CD154) that lacks the intracellular signaling domain and instead fuses the extracellular and transmembrane domains to an isoleucine zipper domain.[12] This creates a membrane-bound trimerized CD40L molecule that lacks intracellular signaling in the TMZ-CD40L-expressing cell but still binds and transmits signals to other cells that express its receptor, CD40. TMZ-CD40L can mature DCs to become potent stimulators of T- and NK cells and can enhance the expression of adhesion receptors on endothelial cells which promotes lymphocyte attachment and migration into inflamed areas.[12,13] On the other hand, CD40L can also play a role in tumor cell death due to a dysregulated signaling cascade whereby CD40 ligation in CD40+ cancer cells can lead to tumor growth inhibition and apoptosis.[11]

The 4-1BB ligand (4-1BBL; CD137L) transgene is the full-length human 4-1BBL gene. It binds to its receptor 4-1BB (CD137) expressed on activated T cells and NK cells. 4-1BBL stimulation of T cells and NK cells protects the cells from activation-induced cell death (AICD) via upregulation of apoptosis inhibitors such as BCL-xL. 4-1BBL stimulation also promotes efficient lymphocyte proliferation and memory T cell formation.[14,15]

The LOAd703 virus is not functional in animal models since it requires human CD46 for infection and cannot replicate even when animal cells are modified to express human CD46. Further, human TMZ-CD40L and 4-1BBL do not cross react with their murine receptor counterparts. Hence, toxicity studies cannot be performed in *in vivo* models. Oncolytic effect can be demonstrated in immuno-deficient xenograft mice implanted with human tumors and treated with LOAd703. This leads to tumor growth control and tumor regression, but in this model, the effect of the transgenes cannot be evaluated since the mice lack adaptive immunity.[13] Effect of the immunostimulatory transgenes can be evaluated in immunocompetent mice implanted with a murine tumor expressing human CD46 and treated with a LOAd703 virus containing murine TMZ-CD40L and 4-1BBL. This leads to the robust stimulation of DCs, T cells and NK cells. Further, LOAd703 can potentiate the effect of anti-PD-L1 antibodies in an animal cancer model.

The major effector mechanisms of LOAd703 are: 1) induction of cell death via either oncolysis or CD40-mediated apoptosis, and 2) activation of the immune system via CD40L, 4-1BBL and the adenoviral backbone. The major effector arm is likely immune activation, but the induction of cell death by oncolysis further strengthens anti-tumor responses due to the release of tumor antigens resulting in tumor antigen-specific immune stimulation.

Detailed preclinical information can be found in the LOAd703 Investigator's Brochure (IB) Section 6.0 NONCLINICAL EVALUATION OF LOAd703 which is an integral part of this protocol.

2.3 Immune Checkpoint Blockade Therapy

2.3.1 Checkpoint Blockade

Immune checkpoints are inhibitory signals that diminish T cell activation and lead to T cell exhaustion. To overcome these inhibitory signals and sustain T cell activation during the anti-tumor response, checkpoint blockade antibodies have been developed to block inhibitory immune checkpoints such as CTLA4 or PD-L1/PD-1.

Recently, checkpoint blockade therapy has shown impressive results in cancer patients, in particular in malignant melanoma. However, most patients are refractory to treatment or become resistant after an initial response. Resistance is likely due to the patient's immune status, e.g., having an immunosuppressive tumor microenvironment with very few activated anti-tumor reactive T cells, which may not depend on PD-L1/PD-1 interactions. Studies combining oncolytic virus with checkpoint blockade inhibition have demonstrated enhanced effect in preliminary reports. The use of Imylgic® with pembrolizumab increased response rates from approximately 30% to 60%.[16]

2.3.2 Atezolizumab

Atezolizumab is a humanized IgG1 monoclonal antibody that targets PD-L1, a ligand expressed on a variety of cell types as well as malignant cells. Overexpression of PD-L1 on tumor cells may be a mechanism of tumor evasion and has been demonstrated to inhibit anti-tumor responses.[17] Atezolizumab inhibits the interaction between PD-L1 and its receptors PD-1 and B7.1 (CD80), which would otherwise inhibit T cell proliferation, cytokine production and cytolytic activity.[18,19] In addition, atezolizumab demonstrates minimal binding to Fc receptors, eliminating detectable Fc-effector function and associated antibody-mediated clearance of activated effector T cells.

Therapeutic blockade of PD-L1 binding by atezolizumab has been shown to improve anti-tumor activity by enhancing tumor-specific T cell responses.[20,21] Atezolizumab shows anti-tumor activity in both nonclinical models and cancer patients and is being investigated as a potential therapy in a wide variety of malignancies. Atezolizumab is approved for the treatment of urothelial carcinoma and non–small cell lung cancer and is currently being studied as a single agent in advanced cancer and adjuvant therapy settings, as well as in combination with chemotherapy, targeted therapy, and cancer immunotherapy.

Detailed preclinical information can be found in the atezolizumab Investigator's Brochure Section 4 Nonclinical Studies which is an integral part of this protocol.

2.4 LOAd703 Previous Clinical Studies

Currently, LOAd703 is being evaluated in combination with standard-of-care (SoC) chemotherapy treatment in two ongoing phase I/II clinical trials. In one of the trials, atezolizumab is added to the combination treatment in a second study arm. Briefly, the trials are open label studies to evaluate dose, safety and efficacy. In both trials, three dose levels are evaluated followed by expansion of the maximum tolerated tested dose. An overview of the trials is shown in Table 1.

2.6.4 Potential Benefits – Rationale for Combining LOAd703 and Atezolizumab

All patients enrolled in this trial have melanoma that cannot be completely resected, and they do not respond to checkpoint antibody therapy targeting the PD-L1/PD-1 pathway (i.e. progress demonstrated using such treatment). Resistance to checkpoint therapy commonly depends on lack of tumor infiltrating T cells, which is a prerequisite for checkpoint therapy. Hence, monotherapy using atezolizumab that targets this pathway is not considered for patients progressing on other antibodies blocking the same pathway.

LOAd703 may offer clinical benefit to these patients by reducing both the tumor and tumor stroma since this oncolytic immunostimulatory adenovirus infects and eradicates tumor cells by oncolysis as well as by inducing anti-tumor and anti-viral immunity. Tumor cells that are not rapidly killed by oncolysis may be cleared by immune responses against the adenovirus since LOAd703 is very immunogenic. In addition, both infected stroma and tumor cells can express the LOAd703 immunostimulatory transgenes TMZ-CD40L and 4-1BBL. Oncolysis will not occur in stroma cells, but infected stroma may boost anti-tumor immune responses before eventually being eradicated by anti-adenovirus immune responses. Thus, LOAd703 has the potential of both reducing tumor size and providing immune activation. Further, the inflammatory capacity of LOAd703 may induce influx of tumor-reactive immune cells into the tumor which may re-sensitize the patients to checkpoint antibodies.

In preclinical models, LOAd703 is a potent maturation agent for DCs.[13] During the maturation, the DCs also upregulates PD-L1 which may be detrimental for long lasting immunity induced by LOAd703. Hence, targeting PD-L1 is an interesting option to enhance LOAd703-mediated immunity. In an experimental melanoma *in vivo* model (B16), the combination of LOAd703 with anti-PD-L1 antibodies showed increased infiltration of T cells, NK cells and DCs in the tumor bed followed by reduced tumor growth. Further, serum cytokines such as IFNg, TNFa and IL27 were increased (see LOAd703 IB Section 6.2.3). In preclinical settings, the anti-PD-L1/LOAd703 combination gave a more robust response than anti-PD-1/LOAd703 combination. Atezolizumab targets anti-PD-L1 and is, in contrast to other anti-PD-L1 antibodies, engineered to reduce antibody-dependent cell-mediated cytotoxicity (ADCC) which may otherwise kill the PD-L1 expressing DCs.

Due to the characteristics of each drug, it is theoretically appealing to induce anti-tumor immunity using LOAd703 in combination with atezolizumab (i.e an activation phase) and thereafter sustain the gained immunity with prolonged atezolizumab conditioning. Hence, this protocol will allow up to 12 LOAd703 injections in combination with atezolizumab, where after gained immune control will be sustained with additional atezolizumab cycles.

There are multiple trials ongoing using different types of oncolytic viruses in combination with checkpoint blockade antibodies (www.clinicaltrials.gov). Interim reports demonstrate benefit[16] but so far there are no final data using adenoviruses in combination with checkpoint blockade antibodies. Likely, the type of transgenes may be equally important as the type of virus used. LOAd703 is the only oncolytic virus in clinical development expressing TMZ-CD40L and 4-1BBL.

Hence, the possible benefits of combining LOAd703 with atezolizumab in these patients may induce anti-tumor immunity resulting in tumor size reduction and/or slower rate of tumor growth. Moreover, due to the systemic character of T cell-mediated anti-tumor immunity, it is possible that this combination treatment could even eradicate escaped tumor cells. This potential benefit could control metastases and prevent or prolong the time to the development of new metastases. Overall, the generated immune control may result in survival benefits for the patients.

2.7 Rationale for the Phase I/II Doses

This study will begin at the LOAd703 dose level $1x10^{11}$ VP per treatment (cohort 1), and if safe, the dose will be escalated to $5x10^{11}$ VP per treatment every 3 weeks (q3w) (cohort 2). These dose levels have been demonstrated to be safe in the ongoing LOKON001 (NCT02705196) and LOKON002 (EudraCT 2017-002565-22) trials where patients were treated i.t. every other week (q2w) with 6-12 and 8 LOAd703 treatments, respectively. No intra-patient dose escalation is allowed. Dose reductions may be applicable due to toxicity (see 5.5 *Dose Limiting Toxicity (DLT*), and 5.6.8 *LOAd703 Dose Modifications*).

Atezolizumab will be given as a fixed dose (1200 mg, q3w) which is the approved dose level and schedule for the treatment of urothelial carcinoma and non–small cell lung cancer. Atezolizumab may be subject to dose adjustments due to toxicity (see 5.7.7 *Atezolizumab Dose Modifications*).

LOAd703 and atezolizumab will be administered at the same day beginning with LOAd703. The $T_{1/2}$ for atezolizumab is 27 days which is the basis of the approved atezolizumab cycle (q3w). Hence, the antibody will be present when transgenes are upregulated on infected cells by LOAd703 which cause DC activation over time. The antibody will also be present to prevent PD-L1/PD-1 interactions by activated T cells and tumor cells, MDSCs and M2 macrophages that may also upregulate PD-L1 and are continuously present to hamper immune activation. In theory, there is no scientific difference if they are administered the same day or within the same week. In the rapidly growing B16 model, it is not possible to elaborate on the dosing schedule. For the patients, it is beneficial to receive the treatments at the same day and in similar cycles to reduce hospital visits which in turn may increase quality of life.

2.8 Patient Population

Melanoma is a cancer that develops from melanocytes in the epidermal layer of the skin. Melanoma is the fifth most common cancer among men and women.[32] It is often diagnosed in older patients but is also one of the most common cancers in young adults, especially young women.[33] Risk factors include sun/UV exposure, skin type (white/fair skin, moles, etc.), genetic disposition, and sex; the risk is higher for women before the age of 50, and higher for men after 50 years of age.[34] More than 90% of melanoma cancers are due to skin cell damage from ultraviolet radiation exposure.[35]

The incidence of melanoma has doubled in the last three decades in the US from an incidence of 11.2 to 22.7 per 100,000.[36] In 2019, it is projected that 96,480 adults in the US (57,220 men and 39,260 women) will be diagnosed with melanoma.[32] Although it accounts for only 1% of all skin cancers, melanoma is the deadliest type of skin cancer, accounting for >90% of skin cancer deaths.[37] In 2019, it is estimated that over 7000 people in the US will die from melanoma.[32]

While localized melanoma is typically curable with surgery, metastasized melanoma in regional and distant areas have a 5-year survival rate of 63% and 20%, respectively.[37] Metastatic melanoma is known to be refractory to traditional therapies like chemotherapy and radiation, and until 2010, there was no evidence-based therapy available that could demonstrate significantly improved overall survival (OS) for patients with unresectable advanced melanoma. In patients with stage IV metastatic melanoma, less than half survived >1 year and only 20% were alive after 3 years. Since 2010, new systemic therapies such as checkpoint inhibitors have improved treatment options for patients with unresectable advanced melanoma.[38] In addition to immunotherapy, targeted therapies (BRAF- and MEK-inhibitors) can be utilized in patients with BRAF mutations, which may account for over half of all melanomas.[34] In 2015, the first oncolytic virotherapy, T-VEC (Imlygic®; talimogen lahparepvec), was approved for the treatment of melanoma (FDA and EMA). T-VEC as well as several other oncolytic viruses, such as CAVATAK, are being actively developed, as monotherapies or as combination therapies together with checkpoint inhibitors.

In this study, patients with melanoma that have received at least one prior line of checkpoint blockade antibody therapy (mono or combination) can be enrolled in the study. Both men and women of all races and ethnic groups are eligible to participate in this trial. However, children are not eligible for participation since safety and tolerability have not yet been determined in adults.

All patients enrolled will receive LOAd703 combined with the checkpoint inhibitor atezolizumab. LOAd703 treatment stimulates DCs, NK and T cells while atezolizumab blocks the inhibitory signaling via PD-L1/PD-1 which may prolong T cell activation and the anti-tumor response.

3.0 TRIAL DESIGN

3.1 Objectives

The primary objective is to determine the tolerability of LOAd703 given by i.t. injection(s) in combination with atezolizumab administered by IV.

The secondary objectives are to determine the antitumor activity as well as the pharmacokinetics and biological mechanisms-of-action of LOAd703 in combination with atezolizumab.

3.2 Endpoints

Primary Endpoints

1) The primary endpoint is safety, determined by the National Cancer Institute Common Toxicity Criteria for Adverse Events (NCI CTCAE) v5.0.

Secondary Endpoints

- Overall response rate evaluated by RECIST v1.1.
- Shedding determined as level of LOAd703 in shedding samples at different time points post-treatment.
- LOAd703 leakage to blood determined as level of LOAd703 in blood at different time points post-treatment.
- Anti-adenovirus immunity determined as anti-viral antibodies and T cells after combination therapy compared to baseline.
- Pharmacokinetics of atezolizumab as determined as the level of atezolizumab in blood at different time points post-treatment.
- Immunity to atezolizumab determined as the level of anti-drug antibodies (ADA) targeting atezolizumab at different time points post-treatment compared to baseline.
- Immune profile as determined by changes in immune cell populations and their activation markers in tumor tissue and blood after combination therapy compared to baseline.
- Protein profile as determined by changes in protein profile in blood after combination therapy compared to baseline.

3.3 Summary of Trial Design

This trial is a multicenter, open label, single arm, dose-escalation Phase I/II trial. The study will have a Bayesian Optimal Interval (BOIN) design (target DLT rate=0.3) to determine the MTD of LOAd703 against a fixed dose of atezolizumab. This design has algorithmic escalation/de-escalation rules like a traditional 3+3 design, but it also allows for specification of the target DLT rate and expanded accrual beyond N=6 with continued and consistent toxicity monitoring. This means that the assessment of DLT rate continues throughout the study, giving a well-defined safety evaluation of the entire study. Refer also to sections *10.0 STATISTICS, 5.5 Dose Limiting Toxicity (DLT) and 5.6.9 LOAd703 Maximum Tolerated Dose (MTD)*.

Efficacy will be evaluated using a one-sided binominal test. Assuming a true response rate of at least 30%, then 25 subjects need to be evaluated at MTD to have a probability of 0.9 of rejecting the null hypothesis (i.e., that the response rate is lower or equal to 5%) using a one-sided test with a significance level of 0.025. The total number of patients to be enrolled in the study to achieve at least 25 evaluable patients at MTD, should not exceed 50.

3.4 Duration of Study

.

Timeline:	First patient in	Q3 2020
	Last patient's last visit	Q1 2024
	Study end	Q12024

After a screening period of up to 3 weeks, each patient will receive a maximum of 12 LOAd703 injections (q3w) in combination with atezolizumab (q3w) after which supportive therapy with atezolizumab alone is continued until the final clinical visits (i.e. maximum 7 additional infusions). The patients will be scheduled for study visits until final clinical follow-up in study week 57.

If both treatments are prematurely discontinued the final clinical follow-up visit could be scheduled earlier, but not until 6 months post final virus administration or 3 weeks post last atezolizumab infusion have passed, whatever comes last.

In total, patients will visit the clinic regularly for a maximum 60-weeks (week -3 to week 57). Thereafter only OS will be recorded every 4th months whereby information will be collected via e.g. telephone calls (US) or medical records (SWE, US) until death or study end.

3.5 End of Study

The End of the treatment stage is defined as the date of the LPLV and the End of study is defined as LPLV.

4.0 SELECTION AND WITHDRAWAL OF PATIENTS

Study patients will be recruited among patients taken care of at the study center but may also include patients referred from other hospitals. Once a patient is enrolled in the trial, the Site Investigator is responsible for the care of the patient.

Patients must meet all the following inclusion criteria and none of the exclusion criteria to be registered in the study:

4.1 Inclusion Criteria

1. Pathological confirmation of melanoma.
2. A life expectancy of at least 3 months as per the investigator.
3. **Valid for Swedish patients:** Patients has locally advanced melanoma or metastatic melanoma, but not eligible for complete resection of melanoma.
   Valid for US patients: Patients has locally advanced melanoma or metastatic melanoma.
4. The patient has measurable disease (e.g., measurable tumor lesions must be present that can accurately be measured in at least one dimension with a minimum size of 10 mm by CT scan and MRI, 10 mm caliper measurement by clinical exam (when superficial), and/or 20 mm by chest X-ray).

5. Patient has at least one injectable tumor lesion that has not been irradiated or has been irradiated but disease progression documented at the site subsequent to radiation therapy.
6. The patient has received appropriate treatment with an anti-PD-1 or anti-PD-L1 antibody with or without an anti-CTLA4.
7. **Valid for Swedish patients:** Patients whose advanced melanoma has a B-Raf mutation must have received appropriate therapy with tyrosine kinase inhibitor(s) and/or MEK inhibitor **Valid for US patients:** Patients whose advanced melanoma has a B-Raf mutation may have received appropriate therapy with tyrosine kinase inhibitor(s) and/or MEK inhibitor as assessed by the investigator.
8. Age $\geq$ 18 years.
9. Eastern Cooperative Oncology Group (ECOG) performance status of 0 to 1.
10. Serum albumin ≥ 2.5 g/dL.
11. Absolute neutrophil count (ANC) $\geq$ 1.0 x 10e9/L.
12. Platelet count ≥ 100 x 10e9/L.
13. Prothrombin (INR) $\leq$ 1.5 or prothrombin time (PT) $\leq$ 1.5 times ULN; and either partial thromboplastin time or activated partial thromboplastin time (PTT or aPTT) $\leq$ 1.5 times the ULN.
14. Bilirubin < 1.5 times the institutional upper limit of normal (ULN).
15. Aspartate aminotransferase (AST) and alanine aminotransferase (ALT) $\leq$ 2.5 ($\leq$ 5 if liver metastases are present) times the institutional ULN.
16. The patient must have signed informed consent.

4.2 Exclusion Criteria

1. Malignant melanoma that is uveal.
2. Subjects considered by the investigator to have rapid clinical progression due to melanoma.
3. Subjects must not have greater than 3 cerebral melanoma metastases, and/or clinically active cerebral melanoma metastases, and/or a requirement for corticosteroid therapy, and/or carcinomatous meningitis regardless of clinical stability.
4. Any concurrent treatment that would interfere with the effect mechanisms of atezolizumab and LOAd703, including, but not limited to, continuous high-dose corticosteroids (>10 mg per day), lymphodepleting antibodies, or cytotoxic agents.
5. Treatment with inhibitors of immune function, such as lymphotoxic monoclonal antibodies (e.g., alemtuzumab), or rapamycin/rapamycin analogs, or cytotoxic agents within 21 days of the first dose of LOAd703/atezolizumab.
6. Therapeutic treatment with systemic antibiotics within 14 days of the first dose of LOAd703/atezolizumab.
7. Treatment with biologic therapy within 21 days of the first dose of LOAd703/atezolizumab.
8. Treatment with cytotoxic anticancer therapy within 14 days of the first dose of LOAd703/atezolizumab.
9. Treatment with wide-field radiation within 14 days of the first dose of LOAd703/atezolizumab.
10. Prior treatment with an adenovirus-based gene therapy.
11. Use of any investigational agents within 21 days of the first dose of LOAd703/atezolizumab.
12. The use of systemic immunostimulatory agents (including, but not limited to, interferons and IL2) are prohibited within 21 days or 5 half-lives (whichever is longer) of the first dose of LOAd703/atezolizumab.

13. Failed resolution/improvement of AEs including those related to anti-PD-1/anti-PD-L1 to grade 0-1 and requirement for treatment with ≥10 mg/day prednisone (or equivalent) for at least two weeks prior to registration.
14. History of CTCAE grade 4 immune-related AEs from monotherapy using an anti-PD-1/anti-PD-L1 antibody.
15. History of CTCAE grade 4 AE that require steroid treatment (>10 mg/day prednisone or equivalent) for >12 weeks.
16. Patients requiring warfarin are not eligible (low molecular weight heparin is permitted).
17. Women who are pregnant (as confirmed by pregnancy test during screening in applicable patients), breastfeeding, or planning to become pregnant during the study period, or women of childbearing potential who are not using acceptable highly effective contraceptive methods. A woman is considered of childbearing potential if she is not surgically sterile or is less than 1 year since her last menstrual period. The following are acceptable as highly effective contraceptive methods: combined (estrogen- and progesterone-containing) hormonal contraception associated with inhibition of ovulation (oral, intravaginal, transdermal), progesterone-only hormonal contraception associated with inhibition of ovulation (oral, injectable, implantable), intrauterine device, intrauterine hormone-releasing system, bilateral tubal occlusion and vasectomized partner or abstinence of heterosexual intercourse during the entire study period (depending on the preferred and usual life style of the subject).
18. Men who do not consent to the use of condoms during intercourse during study participation or has a partner of childbearing potential, who will not use any of the highly effective contraceptive methods exemplified in exclusion criteria no 17.
19. Known active hepatitis B or C infection, or HIV infection.
20. Patients with active, severe autoimmune disease or immune deficiency or previous Guillain-Barré syndrome. Patients with eczema, psoriasis, lichen simplex chronicus or vitiligo with dermatologic manifestations only (e.g., patients with psoriatic arthritis are excluded) are eligible for the study provided all of following conditions are met:
    a. Rash must cover <10% of body surface area.
    b. Disease is well-controlled at baseline and requires only low-potency topical corticosteroids.
    c. Occurrence of acute exacerbations of the underlying condition requiring psoralen plus ultraviolet A radiation, methotrexate, retinoids, biologic agents, oral calcineurin inhibitors, or high-potency or oral corticosteroids within the previous 12 months.
21. History of leptomeningeal disease.
22. Uncontrolled pleural effusion, pericardial effusion, or ascites requiring recurrent drainage procedures (once monthly or more frequently).
23. History of idiopathic pulmonary fibrosis, organizing pneumonia (e.g., bronchiolitis obliterans), drug-induced pneumonitis or idiopathic pneumonitis, or evidence of active pneumonitis on screening chest computed tomography (CT) scan or tested reduced functional respiration capacity. However, history of radiation pneumonitis in the radiation field (fibrosis) is permitted.
24. Unstable angina, uncontrolled cardiac arrhythmia, recent (within 3 months) history of myocardial infarction or stroke, or New York Class III/IV congestive heart failure.
25. Major surgical procedure other than for the malignant melanoma diagnosis, within 4 weeks prior to initiation of the study treatment, or anticipation of the need for a major surgical procedure during the study.
26. Prior allogeneic stem cell or solid organ transplantation.

27. History of severe allergic anaphylactic reactions to chimeric human or humanized antibodies, or fusion proteins.
28. Known hypersensitivity to CHO cell products or any component of the atezolizumab formulation.
29. Uncontrolled intercurrent illness including, but not limited to, psychiatric illness/social situations that in the opinion of the Investigator would compromise compliance to study requirements or put the patient at unacceptable risk.
30. Other malignancy within the past 2 years (not including basal cell or squamous cell carcinoma of the skin, prostate cancer without the need of other treatment than hormones or *in situ* cervix, breast or melanoma).
31. Live, attenuated vaccines (e.g., FluMist®) are prohibited within 4 weeks prior to initiation of study treatment, during treatment, and for 5 months after the final dose of atezolizumab and/or LOAd703.
32. Adenovirus-based vaccines (e.g., Vaxzevria, known as COVID-19 vaccine Astra Zeneca, J&J Covid-19 vaccine) are prohibited 3 months prior to initiation of study treatment, during treatment and 6 months after the final dose of LOAd703.

4.3 Screening, Enrollment and Registration Log, Identification List and Numbering of Subjects

It is the responsibility of the Investigator that all patients considered candidates for the study be listed in the *"Screening, Enrollment and Registration log"*. The reason for rejecting a patient before informed consent is obtained should be specified in the comments field of the log. Patients will receive a consecutive patient number when signing the informed consent and are thereafter considered enrolled as study patients so they can initiate the screening visit. The patient number will have the following format: 003(Lokon study number)-XX(country number)-XX(site number)-XX(consecutive patient number for each site). The trial site will keep an "*Identification list*" of all enrolled patients that connects the subject's identity to their study number.

Study patients who have been screened and excluded before the week 0 visit are considered screening failures and will be noted as such in the *"Screening, Enrollment and Registration log"*. The reason for excluding the patient from the study should be specified in the comments field of the *"Screening, Enrollment and Registration log"*.

When the inclusion criteria and none of the exclusion criteria have been fulfilled, the site can request a study slot by the CRO. When the study slot is confirmed by the CRO, the patient is considered registered in the study and the registration date is documented in the *"Screening, Enrollment and Registration log*. The study investigators participate in regular phone conferences with the CRO and Sponsor to follow the recruitment process and study development.

NOTE: During dose escalation, the slots are limited to 3 per cohort, of which the first patient must receive 2 LOAd703 injections and pass the safety/continuation criteria assessment (at least 3 weeks post the second administration) before patient 2 and 3 can start. During dose escalation it is therefore important to have close communication with the CRO before screening, in order to confirm the timing and avoid having eligible patients ready for treatment when there is no slot available. During dose escalation, the first dose will not be given to two or more patients on the same day (within 24 hours of dosing).

Instructions on communication and the registration procedures are found in the Investigator Site File.

4.4 Withdrawal of Patients

A patient has the right to withdraw his or her consent for participating in the study, at any time, and without giving any specific reason.

The *study treatment* can be discontinued (i.e. from either LOAd703 or atezolizumab or from both treatments) at any time if it is medically necessary, as judged by the Investigator or the Sponsor, based on the development of toxicity as specified in sections *5.5 Dose Limiting Toxicity (DLT), 5.6.8 LOAd703 Dose Modifications, 5.6.9 LOAd703 Maximum Tolerated Dose (MTD)* and *5.7.7 Atezolizumab Dose Modifications.*

If only one of the study treatments is discontinued, the patient still continues with the other study treatment and follows the study schedule according to the protocol.

4.4.1 Off-treatment patient

The patient is discontinued from both LOAd703 and atezolizumab (w0-w33) or from Atezolizumab (w34-w57) if the following criteria are met:

1. The development of toxicity, which precludes further treatment with both study treatments, in the Investigator's judgment, and as specified in sections *5.5 Dose Limiting Toxicity (DLT), 5.6.8 LOAd703 Dose Modifications, 5.6.9 LOAd703 Maximum Tolerated Dose (MTD)* and *5.7.7 Atezolizumab Dose Modifications.*
2. A response that is sufficient to downstage the patient to resectable or borderline resectable disease, in which case the Investigator may decide to pursue chemoradiation and/or surgical resection.
3. Patient has confirmed progressive disease
4. Patient requests to discontinue treatment.
5. Female patient becomes pregnant.

<u>Off-treatment patients:</u> should continue to be followed according to a modified clinical follow-up schedule (see section 6.4 *Evaluation, Modified Follow-Up and Final Follow-Up Visit* and *14.3 Appendix III: Modified Follow-Up Schedule*). This means that patients will continue his/her participation in the study and return to visits for AE assessment, sampling, and radiology (according to protocol) but not receive treatment.

The visits should continue at least until 6 months post final LOAd703 dose, if possible, or 3 weeks after last atezolizumab infusion, whatever comes last, meaning that the final clinical follow-up visit (week 57) may be scheduled earlier. In this way the virus persistence, immunological reactions, late toxicity and anti-tumor effects can still be followed up as described in this protocol.

4.4.2 Survival follow-up patient

If another treatment is initiated due to progressive disease or other reasons, the patient will be followed for survival until death or End of Study (see section *7.5.3 Survival Follow-Up*). The final clinical follow-up visit (week 57) should then be scheduled to collect endpoint data (same assessments as week 57) as close to the start of new treatment as possible.

4.4.3 Off-study patient

The patient will be withdrawn from participation in this trial at any time if any of the following criteria are met:

1. The patient requests to be removed from the trial and withdraws consent.
2. Lost to follow-up/serious noncompliance to protocol.
3. The trial is terminated.

No further data, except for survival, will be collected for these patients (see section *7.5.3 Survival Follow-Up*).

4.5 Replacement of Patients

If a patient does not complete at least two doses of LOAd703 and atezolizumab (for a reason other than LOAd703- or atezolizumab-attributed toxicity), this patient will be replaced by another subject in order to meet the minimum criteria for safety (see section 10.2 *Sample Size*).

4.6 Noncompliance

All instances of noncompliance and all protocol deviations will be recorded.

5.0 TREATMENT OF PATIENTS

5.1 Treatment Overview

The patients will be informed about the study and sign informed consent whereby they are enrolled in the screening phase of the protocol to determine their eligibility, including assessment of health status and radiological evaluation. Imaging obtained within 3 weeks prior to registration may be used for baseline evaluation at the discretion of the Investigator.

Also, if samples have been taken for routine analysis <7 days prior to screening, the results can be used for eligibility evaluation at the discretion of the Investigator.

Within 7 days after registration, the patients will initiate treatment. The patients will be given up to 12 percutaneous LOAd703 i.t. injections combined with atezolizumab, both administered every 3 weeks. Two cohorts consisting of at least 3 patients per cohort will be evaluated. The LOAd703 dose will be escalated, one dose per cohort, to evaluate the following dose levels: 1×10^{11} VP/injection (cohort 1) and 5×10^{11} VP/injection (cohort 2). The treatment will be administered by percutaneous i.t. injection which may be image-guided depending on the tumor location. Atezolizumab will be administrated IV using a fixed dose (1200 mg/infusion).

On the day of the treatment, LOAd703 will be administered first and then atezolizumab, preferably within 1 hour from injection. If, for any reason, the Investigator judges that atezolizumab should not be administered the day of LOAd703 injection, atezolizumab can be given on another day during the treatment week if needed. LOAd703 dose, dose modifications and omissions will be

carried out as per instructions in sections 5.6.8 *LOAd703 Dose Modifications* and 7.2.5 *Continuation Criteria*. Atezolizumab dose, dose modifications, and omissions will be instituted per standard guidelines (US: TECENTRIQ® Full Prescribing Information, Reference ID:4279345, Sweden: SmPC/FASS for atezolizumab/TECENTRIQ®) and 7.2.5 *Continuation Criteria*.

Missed LOAd703 or atezolizumab doses will not be made up in order to maintain adherence to the protocol schedule. For details regarding LOAd703 injection and atezolizumab infusion, see sections 5.6.5 *Preparation of LOAd703 Prior to Treatment* 5.6.6 *Administration of LOAd703, 5.7.4 Preparation of Atezolizumab Prior to Treatment,* and 5.7.5 *Administration of Atezolizumab*, respectively.

For the first 2 LOAd703 treatments of any trial patient, the patients are monitored overnight. Vital signs will be noted before LOAd703 injection and then every 30 minutes for 2 hours, at 3, 4, 5, 6, 8, and 10 hours post dose, and then every 4 hours at the discretion of the Investigator until a final measurement of vital signs at discharge. If no immediate LOAd703-related toxicities occurred after the first 2 treatments that required the patient to be monitored overnight, then the patients will be observed for a minimum of 8 hours post LOAd703 injection at the 3rd and 4th LOAd703 injection, and for a minimum of 6 hours post LOAd703 injection for subsequent treatments before being released from the hospital. Note that the patient should always be planned for an overnight stay but may be released early according to the requirements above at the discretion of the Investigator.

Prior to each LOAd703 and/or atezolizumab treatment (within -2 days), the Investigator must meet the patient to perform a complete AE evaluation (including laboratory values) and assess the toxicity screening to assure that the patient is able to receive next treatment (see sections 7.2.5 *Continuation Criteria* and 4.4 *Withdrawal of Patients*).

Blood samples, urine, oral and rectal swabs, and tumor biopsies will be taken at different time points to evaluate toxicity, pharmacokinetics, immune reactions and possible shedding of the virus particles (refer to *Figure 1: Study time line* and *14.1 Appendix I: Schedule of Events)*. Radiological assessment will be performed at enrollment and thereafter every two months. Six months after the final LOAd703 administration or 3 weeks after the last atezolizumab infusion, whatever comes last, the patients will undergo the final follow-up visit and thereafter enter the survival follow-up. The informed consent form includes a specific question about whether the patient consents to the study team following up their condition every 4 months after the final follow-up visit at the clinic.

OS will be recorded for the patients until death or until End of Study. The survival data may be collected via medical records (SWE, US) or by telephone/email from the Investigator or research nurse/coordinator (US) (see *7.5.3 Survival Follow-Up*). OS will be reported as a descriptive addendum to the study report.

When the first patient in a cohort has received at least 2 doses of LOAd703 and atezolizumab without DLT (as evaluated 3 weeks post the second dose), the next two patients in the cohort can begin treatment but must start treatment at least one day apart (24 hours). Note that enrollment and screening is allowed if slots are available in a cohort at any time. Slots are distributed upon request by the CRO (see section 4.3 for more information).

If a patient does not complete at least 2 doses of LOAd703 (for a reason other than LOAd703 attributed DLT), this patient will be replaced by another subject and not regarded as a DLT evaluable. The BOIN design to evaluate safety allows for continuous DLT assessments and cohort sizes larger than 3 and will thus continue throughout the study.

The decision to escalate dose is taken by the Sponsor after a safety meeting including the Sponsor, CRO, Clinical Project Manager (for taking notes only), Medical Advisor and at least 2 of the site Principal Investigators as a minimum. The maximum tolerated dose (MTD) level will be expanded for continued safety and efficacy evaluation and at least 25 efficacy evaluable patients will be

enrolled at MTD. By using the BOIN design, the actual MTD is therefore determined at the end of the study (see section *5.6.9 LOAd703 Maximum Tolerated Dose (MTD)*). The justification of cohort sizes and the sample size calculation are described in section *10.0 STATISTICS*.

5.2 Treatment Schedule for Study Visits

The LOAd703 treatment schedule and study visits over time are described in *Figure 1: Study timeline* and *14.1 Appendix I: Schedule of Events.* LOAd703 treatment is given up to 12 times. Atezolizumab will be administered continuously during study participation if judged beneficial and safe for the patient by the Investigator. In total, the patient can participate in study visits for 60 weeks (screening week -3 to week 57). If patients are not on-treatment, the blue-labeled study visits for sampling are canceled. On-treatment patients refers to patients continuing LOAd703 and/or atezolizumab treatment. Patients discontinuing treatment (i.e., off-treatment) can have the final follow-up visit scheduled earlier. The final follow-up visit should be at least 24 weeks (6 months) after the last treatment with LOAd703 (if possible) so that the final endpoint data are collected. After the final follow-up visit at the clinic, patient health status and the overall survival will be recorded until death or End of Study, (see *7.5.3 Survival Follow-Up*).

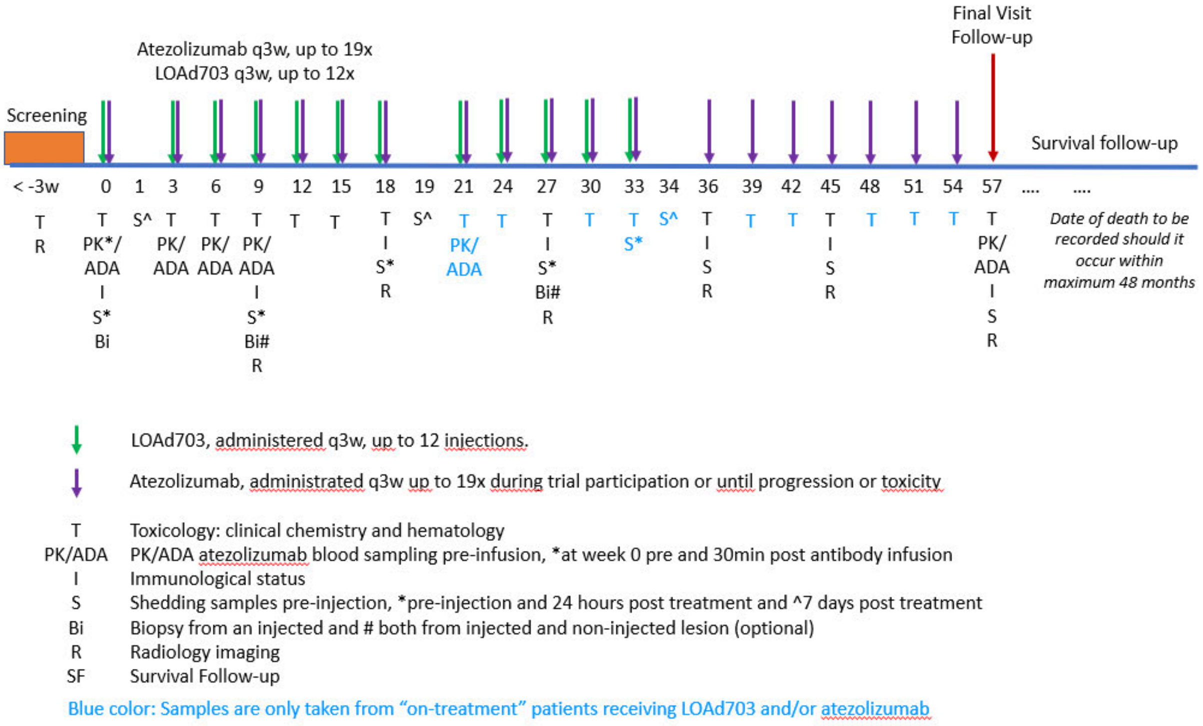

Figure 1. Study timeline. Schedule for study treatments, sampling, and follow-up visits. The planned study participation with visits to the clinic for an individual patient spans a maximum of 60 weeks (screening week -3 to week 57) provided that either LOAd703 or atezolizumab is continued. Note that shedding samples will be collected *24 hours (±4 hours) after treatment for overnight patients, and ^7 days (± 1 day) after treatment at certain time points. Further, atezolizumab PK sampling at week 0 will be performed both prior to infusion and 30 minutes (± 5 min) after infusion. Following the final follow-up clinical visit, patient health status and overall survival will be recorded until death or End of Study (see *7.5.3 Survival Follow-Up*).

5.3 Study Sites

5.3.1 General Description

The patients recruited in this trial will participate in the trial activities at the study sites under the guidance of the site Principal Investigator. As soon as a patient is enrolled in the trial, the Investigator is responsible for the patient.

LOAd703 is administered by i.t. injection. The selection of injectable lesions is decided by the Investigator after consultation with the interventional radiologist. The injections are performed by the radiologist or Investigator following the same procedures as when a biopsy is taken, see "*Instructions for tumor lesion selection for LOAd703 injections and effect evaluation*" in the Investigator Site File.

The hospital personnel involved in this trial have experience and/or training using immunostimulatory adenovirus-based treatments. Study patients are treated in a hospital with a fully equipped emergency care unit. The study nurse/coordinator brings the patient and the LOAd703 virus to the injection suite and stays during the injection, whereupon the patient is transferred to the appropriate hospital unit for monitoring and atezolizumab administration. Trial visits such as sampling and meetings with the study team will also take place at the hospital unit.

5.3.2 GMO Regulation at Trial Site

The LOAd703 virus will be handled only by genetically modified microorganism (GMO)-trained staff. The Sponsor is responsible for GMO training. Trained staff are receiving a GMO certificate, and a copy is archived in the Investigator Site File as proof that they have received appropriate training. Beside information in section *5.6 LOAd703 Drug Product, a* GMO booklet with training material, instructions for GMO handling and accidents (see instruction: *LOKON003 Handling, preparation and treatment with LOAd703*), and a list of certified staff will be available at each trial site.

5.4 Referrals from Other Hospitals

If a patient is referred to a study site by another hospital to participate in this trial, all study activities will be carried out only at the study site including treatments, sampling and assessments. The site Principal Investigator is responsible for all trial patients during their participation in the trial.

5.5 Dose Limiting Toxicity (DLT)

As LOAd703 will be dose escalated, the patients will be monitored for dose limiting toxicity (DLT). DLT is defined as any grade 3 or higher toxicity (CTCAE Version 5.0) that is attributed (definitely, possibly or probably) to LOAd703. A DLT attributed to LOAd703 will lead to dose reduction (see *5.6.8 LOAd703 Dose Modifications*).

Phase I: The first patient on each dose level must have been evaluated for DLT three weeks after receiving the second dose of LOAd703, before patient 2 and 3 in the same cohort can start treatment. Patient 2 and 3 should start treatment at least 24 hours apart.

All 3 patients in the cohort must have been evaluated for DLT before decision on dose escalation is taken for the next cohort.

Phase II: Patient enrollment continues and evaluation for DLTs three weeks after receiving the second dose is done continuously according to BOIN design (see *10.2 Sample Size*).

Toxicity attributed to atezolizumab will not be assessed as DLTs in the study but can still lead to dose modifications (see *5.7.7 Atezolizumab Dose Modifications*).

If a patient experiences an AE grade 3 or higher that can be attributed to the LOAd703 injection procedure (i.e., a bacterial infection, injection site pain, etc.), to disease, or another unrelated cause, it is not a DLT. SAEs due to injection procedure can lead to change of injection procedure (such as change of lesion) instead of treatment discontinuation, depending on the type of AE and the risk of recurrence of the AE.

Patients who experience a toxicity that may be related to both LOAd703 and atezolizumab may only continue treatment if it is judged that the dose of either treatment can be decreased or omitted to avoid toxicity following the rules of dose modifications and LOAd703 DLT assessment (see *5.6.8 LOAd703 Dose Modifications* and *5.7.7 Atezolizumab Dose Modifications*).

No further treatments can be given if related AE remains grade 3 or higher at the time of next treatment with LOAd703 and/or atezolizumab.

DLTs will be handled and recorded in the same way as SAEs, even if the formal SAE criteria are not fulfilled, see section *9.3.1 Reporting to Sponsor*.

5.6 LOAd703 Drug Product

5.6.1 LOAd703 Brief Description

**Active substance**:	Modified adenovirus serotype 5/35 containing a CMV promoter-driven transgene cassette with human transgenes encoding membrane-bound CD40 ligand (TMZ-CD40L) and full-length 4-1BBL.
**Generic name:**	Delolimogene mupadenorepvec
**Company code name:**	LOAd703
**Manufacturer**:	Baylor College of Medicine, Houston, TX.
**Dose**:	$1x10^{11}$–$5x10^{11}$ VP in suspension.
**Dosage form**:	Delolimogene mupadenorepvec virus particles in suspension.
**Formula**:	Delolimogene mupadenorepvec in a TRIS buffer with pH 8 containing sodium chloride and glycerol. The virus can be further diluted using physiological saline (0.9% NaCl) (see section 5.6.5 *Preparation of LOAd703 Prior to Treatment*).
**Storage:**	<-70°C (±10°C)
**Instructions for use**:	LOAd703 is a genetically modified organism (GMO) and national regulations for handling GMO must be followed. Personnel must be trained to handle GMO. Personnel handling LOAd703 must wear gloves, eye and mouth protection as well as protective clothing. Tubes and other disposables that have been in direct contact with the vector need to be neutralized in 1% Virkon® solution, or equivalent disinfectant, for at least 10 minutes and thereafter disposed in biohazard containers. Alternatively, contaminated objects can be sterilized in autoclave boxes and then disposed in waste boxes. Needles and other sharp items will be discarded

in a biohazard container for sharp items without prior sterilization in Virkon®.

Administration: The prescribed virus dose in suspension is administered by i.t. injection, that can be image-guided, into ≤3 lesions/administration.

Mechanism-of-action: LOAd703 induces expression of immunostimulatory proteins in cancer cells, as well as cells comprising the tumor microenvironment, which in turn activates DCs, T cells, and NK cells, and drives M1 macrophage differentiation. LOAd703 induces an anti-tumor immune response that is both local and systemic. Further, infected tumor cells drive virus replication, resulting in tumor-specific oncolysis with antigen release.

5.6.2 LOAd703 Packaging and Labeling

LOAd703 stock virus is stored in 650 µl suspension in cryogenic vials. All study medication will be labeled with information according to national and local regulations.

5.6.3 LOAd703 Storage and Handling

Adenoviral vectors are commonly stable in <-70°C (±10°C) for >10 years. The stability of the LOAd703 virus batch is evaluated annually to determine its specific stability over time. The virus is thawed in a refrigerator at +4°C (± 2°C), or on wet ice, and further stored in the refrigerator or on wet ice until use.

The unopened virus vial shall be used for patient treatment within 24 hours from thawing.

An opened virus vial and/or diluted virus must be used within 5 hours from thawing. The thawed virus suspension is transported on wet ice to the suite for injection.

Adenoviral vectors are GMOs. Remaining virus or disposable objects such as vials that have been in direct contact with virus needs to be neutralized in containers containing 1% Virkon® solution, or equivalent disinfectant, prior to their disposal in a biohazard container/box. Alternatively, the contaminated objects can be sterilized in autoclave boxes and thereafter disposed in waste boxes. Needles and other sharp items will be discarded in a biohazard container for sharp items without prior sterilization in Virkon®.

Nondisposable instruments that have been in direct contact with the virus will be cleaned with 1% Virkon® solution, or equivalent disinfectant (soak object with Virkon®/disinfectant and incubate for 10 min before cleaning with paper using gloves), or sterilized according to routine hospital procedures for reused objects.

5.6.4 LOAd703 Accidents

Always inform the site Principal Investigator about accidents with LOAd703 and document the type of accident and any actions taken. Accidents should be reported to the CRO using the LOAd703 GMO Accidents Report Form.

LOAd703 suspension leakage: Inactivate the LOAd703 suspension with Virkon® or equivalent disinfectant by gently pouring Virkon®/disinfectant on the virus. Let it inactivate for at least 10 minutes before cleaning with paper using gloves. Dispose of the paper and gloves in a biohazard box.

LOAd703 suspension on clothes: Soak the contaminated item in Virkon®/disinfectant and inactivate the LOAd703 virus for 10 minutes. Dispose the clothes in a biohazard box.

LOAd703 suspension on skin: Rinse with water. Wash with soap and rinse with water. Repeat washing. Soak the skin in disinfectant and let air-dry. Seek medical care if inflammation or irritation develops. Report the incident according to routine hospital procedures.

LOAd703 suspension in the eyes: Rinse with eye wash. Seek medical care if inflammation or irritation develops. Report the incident according to routine hospital procedures.

5.6.5 Preparation of LOAd703 Prior to Treatment

For detailed instructions from the Sponsor, see instruction: "*LOKON003 Handling, preparation and treatment with LOAd703*" in the Investigator Site File.

LOAd703 will be delivered frozen in vials containing 650 µl of virus in suspension. The frozen vial will be thawed at the clinic on wet ice or in a refrigerator +4°C (±2°C) according to the Sponsor's instructions. Thawed, unopened virus will be stored in the refrigerator at +4°C (±2°C) for maximum 24 hours.

The thawed LOAd703 virus can be used directly or may be diluted prior use depending on the patient dose and number of lesions to be injected (see 5.6.6 *Administration of LOAd703*). An opened vial and/or diluted virus must be injected within 5 hours after preparation.

Dilution (if applicable)

The LOAd703 virus can be diluted using physiological saline (0.9% NaCl) following the Sponsor's instructions. In brief, at the time of injection LOAd703 is thawed and drawn into a 1 ml BD Luer-Lok™ syringe, and then transferred to a tube containing dilution solution. Thereafter, LOAd703 is ready to use and can be kept on wet ice or at +4°C (±2°C) for 5 hours post -preparation.

Dose level 1: $1x10^{11}$ VP (starting dose level in cohort 1)

For patients given dose level 1, LOAd703 must be diluted prior to use. The patient will be treated with a total of $1x10^{11}$VP suspended in 500 µl per lesion to be injected. Up to 3 lesions can be selected for injection (see 5.6.6 *Administration of LOAd703*). Hence, the prescribed dose will be suspended in 500 µl (1 lesion), 1000 µl (2 lesions) or 1500 µl (3 lesions). Preparation instructions are provided by the Sponsor.

Dose level 2: $5x10^{11}$ VP (starting dose level in cohort 2)

For patients given dose level 2, the patient will be treated with 500 µl of the LOAd703 virus suspension per lesion to be injected. Up to 3 lesions can be selected for injection (see 5.6.6 *Administration of LOAd703*). Hence, the prescribed dose will be administered un-suspended in 500 µl (1 lesion), or suspended in 1000 µl (2 lesions) or 1500 µl (3 lesions). Preparation instructions are provided by the Sponsor.

Upon use

LOAd703 is drawn into a 1-2 ml BD Luer-Lok™ syringe bedside. For percutaneous i.t. injection, a 0.9x120 mm, 20G, cannula fitting luer lock (screw-on device to prevent pressure-mediated separation of the syringe from the cannula during injection) is attached to the syringe. If more than one lesion is injected, it is advised to prepare one syringe per injection site.

5.6.6 Administration of LOAd703

Detailed instructions are provided by the Sponsor, see "*Instructions for tumor lesion selection for LOAd703 injections and effect evaluation*" in the Investigator Site File.

Selection of Tumor Lesion

The Investigator and the radiologist assess together which lesion(s) are suitable for direct or image guided injection. The prescribed virus dose in suspension is administered by i.t. injections into ≤3

lesions per treatment occasion. The lesion(s) are numbered so they can later be identified for repeated injections and measurements since the injected lesion(s) may change over the course of treatment. For example, if a lesion disappears or becomes necrotic, another lesion will be selected. Further, if an injected lesion does not decrease in size over time, it may be advisable to change lesion since a local immune response against the virus may be present. It is advisable to number more lesions than will initially be targeted for injection and measurement. In this way, it will be easier to later select new lesions and also to monitor responses on all injected and non-injected lesions.

Any tumor lesion can be chosen independently of site. However, a lesion which is easily accessible without risk of bowel penetration, or penetration of large vessels, should be chosen if possible. Further, consider that the injected lesion may be swollen upon treatment and estimate the risk of affecting nearby tissues when selecting the lesion. A necrotic lesion should be avoided since the LOAd703 virus requires viable cells to function. A tumor-engaged lymph node may also be injectable as long as it is measurable (≥15 mm).

Administration

Before the day of treatment, the Investigator will determine if 1, 2 or up to 3 lesions will be injected/treated. The total dose will then be divided between the selected number of injection sites so that 500 µl will be injected per site (see section *5.6.5 Preparation of LOAd703 Prior to Treatment*).

If a selected lesion has been reduced, or for other reasons is considered non-injectable before all 12 treatments are given, a new lesion can be selected for further injections. If this occurs, an explanation describing why the lesion was changed should be documented. For tumors easily accessible to percutaneous injection, LOAd703 can preferably be injected by ultrasound-guided percutaneous injection of the selected tumor lesion(s). Other imaging techniques such as computer tomography (CT)-guided injections may also be used at the discretion of the Investigator. Subcutaneous lesions, clearly visible to the eye, may be injected without image guidance.

To confirm identification and visually follow their appearance, these lesions may be photographed, if applicable.

Prior to each administration (within -2 days), the Investigator must perform a complete AE evaluation and toxicity screening to ensure that the continuation criteria (see section *7.2.5 Continuation Criteria*) are fulfilled and to confirm that the patient is able to receive further LOAd703 injections.

On the day of LOAd703 injection, pre-medication or post dose prophylaxis with paracetamol (1g, oral), NSAID (e.g., ibuprofen 400 mg oral) ± low dose corticosteroids (e.g., 4mg betamethasone IV) may be administered according to the Investigator's judgement to reduce risk of immune reactivity to virus capsids. Patients receiving LOAd703 as a percutaneous injection may receive an anti-anxiety drug such as a benzodiazepine prior to the procedure. Prophylactic antibiotics such as IV administered fluroquinolone (Levaquin) is recommended if there is a risk of penetrating bowels or other circumstances that increase risk, in the opinion of the Investigator. Local anesthesia is commonly not needed prior to LOAd703 injection but is recommended when the biopsy is taken.

5.6.7 LOAd703 Unused Clinical Trial Supplies

All unused trial supplies of frozen LOAd703 will be returned to the Sponsor unless the Sponsor decides otherwise. Thawed, unused/used LOAd703 should be disposed in biohazard waste boxes after inactivation in 1% Virkon® or equivalent disinfectant for at least 10 minutes. Alternatively, the virus-containing vials can be disposed in autoclave boxes and sterilized prior to disposal according to routine hospital waste procedures.

5.6.8 LOAd703 Dose Modifications

Intra-individual *escalation (increase)* of LOAd703 dose during the study is not allowed.

Individual dose *de-escalation (decrease)* of LOAd703 is allowed if the patient experiences transient, manageable grade 3 symptoms which, in the opinion of the Investigator together with the Sponsor's Medical Advisor, will not put the patient at increased risk of severe toxicity.

For dose level 1 ($1x10^{11}$ VP) patients in need of dose de-escalation, the dose is reduced to $5x10^{10}$ VP.

For dose level 2 ($5x10^{11}$ VP) patients in need of dose de-escalation, the dose is reduced to $1x10^{11}$ VP.

No further treatments can be given if a related AE remains grade 3 or higher at the time of the next treatment with LOAd703.

If a grade 3 symptom related to the LOAd703 virus also occurs after a dose reduction, the patient will be withdrawn from treatment, but will continue follow-up according to the study schedule.

5.6.9 LOAd703 Maximum Tolerated Dose (MTD)

The maximum tested and tolerated dose (MTD) for LOAd703 is defined as the highest safe dose tested in the study based on the BOIN assessment (see
10.0 STATISTICS).

5.6.10 LOAd703 Continuation of Treatment Post Study

The study drugs are not available for patients post study without approval by the Sponsor, the ethics committee (IEC/IRB), and by the relevant regulatory authorities. The patients will discuss with the physician the next steps for their cancer treatment.

5.7 Checkpoint Blockade Therapy with TECENTRIQ®/Atezolizumab

5.7.1 Atezolizumab Brief Description

**Active substance**:	Humanized monoclonal antibody based on a human IgG1 framework containing heavy chain V_HIII and light chain VκI subgroup sequences.
**Generic name:**	Atezolizumab
**Manufacturer**:	F. Hoffmann-La Roche Ltd., Basel, Switzerland
**Dose**:	1200 mg, q3w
**Dosage form**:	The product is supplied as a sterile liquid in a single-use, 20-mL glass vial containing approximately 20 ml (1200 mg; 60 mg/mL) of atezolizumab.
**Formula**:	Atezolizumab in histidine acetate buffered at pH 5.58 containing sucrose and polysorbate 20.

Storage: Refrigerated at 2°C-8°C (38°F-46°F).

Administration: Administration of atezolizumab will be performed in a monitored setting where there is immediate access to trained personnel and adequate equipment and medicine to manage potentially serious reactions. No premedication is permitted prior to the first infusion. However, if the patient experienced an infusion-related reaction with any previous infusion, premedication with antihistamines, antipyretics, and/or analgesics may be administered for subsequent doses at the discretion of the Investigator.
Atezolizumab should be infused over 60 (±15) minutes for the first infusion. For subsequent infusions, atezolizumab should be infused over 30 (±10) minutes if the previous infusion was tolerated without an infusion-related reaction, or 60 (±15) minutes if the patient experienced an infusion-related reaction with the previous infusion.

Mechanism-of-action: Atezolizumab binds to PD-L1 with high affinity and completely blocks binding to receptors B7.1 and PD-1.

5.7.2 Atezolizumab Packaging and Labeling

The product is supplied as a sterile liquid in a single-use, 20-mL glass vial containing approximately 20 ml (1200 mg; 60 mg/mL) of atezolizumab. It will be prepared for infusion in 250 mL IV infusion bags (see *5.7.4 Preparation of Atezolizumab Prior to Treatment*). All study medication will be labeled with information according to national and local regulations.

5.7.3 Atezolizumab Storage and Handling

Atezolizumab must be refrigerated at 2°C-8°C (38°F-46°F) upon receipt until use. Atezolizumab should not be used beyond the expiration date provided by the manufacturer. No preservative is used in the atezolizumab drug product or the diluent; therefore, the vial is intended for single use only. Discard any unused portion of drug remaining in the vial. Vial contents should not be frozen or shaken and should be protected from light by keeping the vial in the outer carton.

5.7.4 Preparation of Atezolizumab Prior to Treatment

For IV administration, atezolizumab (1200mg per vial) will be administered in 250 mL IV infusion bags containing 0.9% NaCl and infusion lines equipped with 0.2 or 0.22 μm in-line filters. The IV bag may be constructed from polyvinyl chloride, polyethylene, or polyolefin (composed of polyethylene and polypropylene PP); the infusion line may be constructed from polyvinyl chloride, polyethylene, polybutadiene, or polyurethane; and the 0.2 or 0.22 μm in-line filter may be constructed from polyethersulfone or polysulfone. Other materials should be avoided if possible.

Atezolizumab can be diluted to concentrations between 2.4 mg/mL and 9.6 mg/mL in IV bags containing 0.9% NaCl. Atezolizumab must be prepared/diluted under appropriate aseptic conditions as it does not contain antimicrobial preservatives. The prepared solution for infusion should be used immediately to limit microbial growth in case of potential accidental contamination. If it is not used immediately, in-use storage time and conditions prior to use are the responsibility of the user. For fixed dosing of 800, 840 or 1200 mg in 250 mL IV infusion bags, the dose solution may be stored at 2°C-8°C (38°F-46°F) for 24 hours or at ambient temperature ≤25°C (77°F) for 8 hours. This time includes storage and time for administration for infusion. If the dose solution is stored at 2°C-8°C (38°F-46°F), it should be removed from refrigeration and allowed to reach room

temperature prior to administration. Do not shake or freeze infusion bags containing the dose solution.

5.7.5 Administration of Atezolizumab

Administration of atezolizumab will be performed in a monitored setting where there is immediate access to trained personnel and adequate equipment and medicine to manage potentially serious reactions. No premedication is permitted prior to the first infusion. However, if the patient experienced an infusion-related reaction with any previous infusion, premedication with antihistamines, antipyretics, and/or analgesics may be administered for subsequent doses at the discretion of the Investigator.

Atezolizumab will be administrated IV using a fixed dose (1200 mg/infusion). Infusion start and stop time will be recorded in the eCRF and any interruptions during infusion should be explained.

<u>First infusion instructions</u>

- No premedication is permitted prior to the first atezolizumab infusion, which means that the infusion should be administered the day before or the day(s) after LOAd703 administration.
- Vital signs (pulse rate, respiratory rate, blood pressure and temperature) should be measuredat the timepoints described under section 7.2.6 Vital Signs; 1st atezolizumab infusion. .
- Atezolizumab should be infused over 60 (±15) minutes.
- If clinically indicated, vital signs should be measured every 15 (±5) minutes during the infusion and at 30 (±10) minutes after the infusion.
- Patients should be informed about the possibility of delayed post-infusion symptoms and instructed to contact their study physician if they develop such symptoms.

<u>Subsequent infusion instructions</u>

- Atezolizumab should be administered preferably within an hour after LOAd703 injection. If, for any reason, the Investigator judges that atezolizumab should not be administered the day of LOAd703 injection, atezolizumab can be given on another day during the treatment week. LOAd703 dose, dose modifications and omissions will be instituted as per instructions in section *5.7.7 Atezolizumab Dose Modifications*.
- If the patient experienced an infusion-related reaction with any previous infusion, premedication with antihistamines, antipyretics, and/or analgesics may be administered for subsequent doses at the discretion of the Investigator.
- Vital signs (pulse rate, respiratory rate, blood pressure and temperature) should be measuredat the timepoints described under section 7.2.6 Vital Signs; 2nd atezolizumab infusion and onwards..
- Atezolizumab should be infused over 30 (±10) minutes if the previous infusion was tolerated without an infusion-related reaction, or 60 (±15) minutes if the patient experienced an infusion-related reaction with the previous infusion.
- If the patient experienced an infusion-related reaction with the previous infusion or if clinically indicated, vital signs should be measured during the infusion and at 30 (±10) minutes after the infusion.

5.7.6 Atezolizumab Unused Clinical Trial Supplies

All unused trial supplies of atezolizumab will be returned to the Sponsor, unless the Sponsor decides otherwise. Diluted product for infusion that is not used will be considered medical waste and destroyed/disposed of according to routine hospital procedures.

5.7.7 Atezolizumab Dose Modifications

Atezolizumab dose, dose modifications, and omissions will be instituted per standard practice (US: TECENTRIQ® Full Prescribing Information, Reference ID:4279345, Sweden: SmPC/FASS for atezolizumab/TECENTRIQ®) and according to the continuation criteria (see 7.2.5 *Continuation Criteria*). Missed doses will not be made up in order to maintain adherence to the protocol schedule.

5.7.8 Continuation of Atezolizumab Treatment Post Study

The study drugs are not available for patients post study from Sponsor without approval from the Sponsor, the ethics committee (IEC/IRB), and by the relevant regulatory authorities. The patients will discuss with the physician the next steps for their cancer treatment.

5.8 Approved and Non-Approved Concomitant Treatment

Patients can receive full supportive care while on this study. The use of high dose immunosuppressive substances such as continuous corticosteroids (>10 mg/day) may interfere with the effect of LOAd703 and/or atezolizumab and should be discussed with the Sponsor's Medical Advisor. However, corticosteroids are recommended to treat AEs related to LOAd703 and/or atezolizumab.

Anti-anxiety drug such as a benzodiazepine can also be used prior to the procedures. Prophylactic antibiotics, such as IV administrated fluroquinolone (Levaquin), are typically standard.

Systemic corticosteroids and TNF-α inhibitors may attenuate potentially beneficial immunologic effects of treatment with atezolizumab. Therefore, in situations in which systemic corticosteroids or TNF-α inhibitors would be routinely administered, alternatives should be considered (e.g., antihistamines). If the alternatives are not feasible, systemic corticosteroids and TNF-α inhibitors may be administered at the discretion of the Investigator.

Concomitant use of herbal therapies is not recommended because their pharmacokinetics, safety profiles, and potential drug–drug interactions are generally unknown. However, herbal therapies not intended for the treatment of cancer may be used during the study at the discretion of the Investigator.

Live, attenuated vaccines (e.g., FluMist®) are prohibited within 4 weeks prior to initiation of study treatment, during treatment, and for 5 months after the final dose of atezolizumab and/or LOAd703.

Adenovirus-based vaccines (e.g., Vaxzevria, known as COVID-19 vaccine Astra Zeneca, J&J Covid-19 vaccine) are prohibited 3 months prior to initiation of study treatment, during treatment and 6 months after the final dose of LOAd703.

Need for palliative surgery as well as palliative local radiotherapy, e.g., for brain or bone metastasis, during the trial is accepted unless this prohibits tumor evaluation according to the RECIST-criteria.

5.9 Monitoring Subject Compliance

Since LOAd703 and/or atezolizumab will be administered by the study personnel, compliance with study drug administration will be ensured and documented in the eCRF. Patient compliance with concomitant medications and protocol-specified assessments will be recorded in the eCRF.

6.0 STUDY EVALUATIONS

Blood samples and tumor biopsies will be taken at different time points to evaluate toxicity, virus pharmacokinetics, virus shedding (blood, biopsies, oral and rectal swabs, and urine) and immune reactions (Figure 1). Imaging will be used at several time points to determine tumor size. Patients will undergo their final follow-up evaluation visit at least six months post final virus injection

6.1 Schedule of Events

All study evaluations are summarized in *14.1 Appendix I: Schedule of Events*.

6.2 Screening

Before beginning the screening visit, the patient will be informed about the trial and sign informed consent whereupon the patient is enrolled in the trial. The patient will be evaluated for health status, pregnancy (if applicable) and tumor load (by appropriate radiological imaging). If the patient has been subjected to radiology examination within three weeks prior to registration, this imaging can be reused for evaluation at the discretion of the Investigator.

Also, if samples have been taken for routine analysis <7 days prior to screening, the results can be used for eligibility evaluation at the discretion of the Investigator.

If all of the inclusion criteria and none of the exclusion criteria are met, the patient is registered to participate in the trial.

6.3 Treatment Study Visits

Figure 1 and Appendix 1 (Schedule of Events) shows the treatment schedule and timing of the study visits. Patients will undergo 12 LOAd703 treatments (q3w) administered by i.t. injection until study week 33. Atezolizumab infusions (q3w) will continue during the study until progression or toxicity requires treatment discontinuation. On the day of the treatment, LOAd703 will be administered first and then atezolizumab. Atezolizumab can be given on another day during the treatment week if needed. Note that the first atezolizumab infusion should be given the day after virus injection since no pre-medication is allowed.

The treatments are scheduled on any of the designated study week working days. If the treatments are delayed to the next week, then they must be omitted in order to adhere to the protocol schedule.

Laboratory samples (toxicity, immunological assessment, pharmacokinetics and shedding) are taken at study visits as described in section *7.1 Collection of Blood and Tissue Samples and Storage*.

If both treatments are discontinued, a modified follow-up schedule will apply (see section *6.4 Evaluation,* Modified Follow-Up and *Final Follow-Up Visit*).

6.4 Evaluation, Modified Follow-Up and Final Follow-Up Visit

During evaluation visits, the patient will be evaluated for health status, toxicity, and treatment effect on tumor size (radiology exam). Laboratory samples are taken for immunology, pharmacokinetics and shedding. The final follow-up visit is scheduled at study week 57 (see Figure 1).

If both LOAd703 and atezolizumab are discontinued (i.e., off-treatment patient), the patient should still be followed up and participate in the evaluation visits according to the Modified Follow-Up schedule, unless consent is withdrawn as well. Modified follow-up: patients should complete visits until visits week 18(19) and after visit week 18(19), the patients will return for visits weeks 27, 36, 45, and 57, however sampling at weeks 21, 24, 30, 33, 34, 39, 42, 48, 51, and 54 are omitted (see Appendix *III: Modified Follow-Up Schedule*).

For off-treatment patients, the final follow-up visit can be scheduled earlier but preferably at 24 weeks (6 months) after the last LOAd703 dose to allow completion of the shedding sampling, if possible.

If another treatment is initiated due to progressive disease or other reasons, the patient will only be followed for survival (see section *7.5.3 Survival Follow-Up*). The final clinical follow-up visit (week 57) should then be scheduled to collect endpoint data (same assessments as week 57) as close to the start of new treatment as possible.

If patients are prematurely withdrawn from the study (i.e., survival follow-up patients), they will be followed for a minimum of 30 days after the last LOAd703 and/or atezolizumab treatment for the occurrence of new or ongoing adverse events (AE), if possible. The final clinical follow-up visit should be scheduled as soon as possible to collect endpoint data prior to the patient’s withdrawal, if possible. After the final evaluation visit, patients enter survival follow-up and date of death will still be collected (see *7.5.3 Survival Follow-Up*).

7.0 STUDY ASSESSMENTS

7.1 Collection of Blood and Tissue Samples and Storage

7.1.1 Blood Samples, Biopsy and Shedding

Toxicity (T)

For toxicity evaluations (T), blood samples are taken according to local routines and sent to the local hospital laboratory for clinical chemistry analyses (see sections *7.2.10 Blood Chemistry* and *7.2.11 Hematology*).

Immunology, shedding and pharmacokinetics (PK)/anti-drug antibodies (ADA)

Samples are collected according to the “*LOKON003 Laboratory Manual*” provided by the Sponsor (see Investigator Site File) using the provided tubes/swab kit. The samples are collected prior to initiation of pre-medications or treatments at the study visit at the time points indicated in Figure 1 (Study timeline), and Appendix 1(Schedule of events), unless indicated otherwise in the text below.

In brief:

- For immunological assessments (I): 4 X 7 ml heparin tubes (green cap) are collected and analyzed as described in section *7.3 Immunological Assessments.*
- For LOAd703 shedding and PK : 1 serum tube (red cap), 1 urine sample, 1 oral swab, and 1 rectal swab are collected and analyzed as described in sections *7.4.1 Pharmacokinetics in Blood* and *7.4.3 Analysis of LOAd703 Shedding in Oral and Rectal Swabs, and Urine*.
- For atezolizumab PK and ADA (PK/ADA): 2 gold-capped serum tubes are collected, one for each analysis (section *7.3.2 Anti-Drug Antibodies (ADA)*) and ADA (section *7.4.1 Pharmacokinetics in Blood*).
- For biopsy (Bi): needle biopsies from the injected (weeks 0, 9, 27) and a non-injected (optional; weeks 9 and 27) lesion are taken according to hospital routines. If subjects

undergo a subsequent resection while on the study, a portion of the biopsy tissue will be requested. Biopsies are evaluated for immune assessment (section *7.3.4 Cytokines and Other Protein Responses*) and presence of LOAd703 (section *7.4.2 Presence of LOAd703 in Tumor Lesions*) or tumor markers (section *7.5.2 Tumor Markers*) . Analysis of the biopsy within the study is done via the Research Laboratory, if applicable or send for analysis within Sweden and EU or third country (US, UK). After the analysis, sample will be destroyed or returned to the biobank.

Each study site will prepare the samples for freezing (<-70°C) according to the instructions provided by the Sponsor "*LOKON003 Laboratory Manual*" (see Investigator Site File).

7.1.2 Storing Samples in Biobank

Serum, plasma, urine, oral and rectal swabs, and tumor biopsy samples will be archived for analysis to define safety, mechanisms-of-action and efficacy of the product. For new analyses outside the scope of this study, ethical approval will be requested.

After shipment from the study sites, the samples will be stored in the Uppsala Biobank (827) according to national legislation and handled/owned by the Department of Immunology, Genetics and Pathology, Uppsala University (Research Analysis Laboratory). The samples are labeled with the patient's study number and not with personally identifiable information. The identification list for decoding the study IDs shall remain at the study site.

7.2 Safety Parameters

7.2.1 Demographics

At screening only:

-Age
-Gender
-Race

7.2.2 Body Measurements

At screening only:

-Height

At screening and final follow-up visit:

-Weight

7.2.3 Medical History/Patient History

At screening only:

-Prior and ongoing medical illness and conditions
-Baseline symptoms
-Date of melanoma diagnosis
-Melanoma TNM staging
-Listing of known genetic tumor markers
-Previous anti-tumor therapy (e.g., surgery, chemotherapy, radiotherapy, targeted therapy)
-Previous or ongoing smoker

7.2.4 Physical Exam

-Physical assessment (screening, week 9, 18, 27, 36, 45, and at final follow-up visit week 57). The physical assessment includes, but is not limited to, assessment of head/neck, chest/lungs, skin, abdomen, lymph nodes and the cardiovascular system. Other assessments should be added as applicable. The outcome will be recorded as "normal" or "abnormal". Abnormal findings will be assessed as "clinically significant" or "not clinically significant".

7.2.5 Continuation Criteria

Within -2 days prior to each LOAd703 and/or atezolizumab treatment (starting week 0), the Investigator will meet with the patient to confirm that he/she is eligible to continue treatment by reviewing:

- laboratory values
- No AEs/SAEs/DLTs have occurred that require dose reduction or discontinuation of treatment (see sections *5.5 Dose Limiting Toxicity (DLT), 5.6.8 LOAd703 Dose Modifications,* and *5.7.7 Atezolizumab Dose Modifications*). Note that additional treatments are not allowed if an ongoing related AE is still grade 3 or higher.
- No withdrawal criteria are fulfilled (see section *4.4 Withdrawal of Patients*).
- ECOG
- The patient is fit to receive another injection, in the opinion of the Investigator.

If the patient is not fit to receive LOAd703 or atezolizumab due to ongoing AEs, the treatment will be omitted. Missed LOAd703/atezolizumab doses will not be made up in order to maintain adherence to the protocol schedule.

7.2.6 Vital Signs

At screening and final follow-up visit: Blood pressure and pulse will be noted.

Following LOAd703 treatments: Vital signs (blood pressure, pulse and temperature) will be noted before LOAd703 injection and then at the following time points post injection: 30 min, 60 min and 90 min (±5 minutes), 2h, 3h, 4h 5h, 6h and 8h (±10 minutes) and 10h. Thereafter every 4 hours (14h, 18h, 22h, 26h, 30h (±30 minutes)) at the discretion of Investigator and until a final measurement of vital signs at discharge.

Following atezolizumab treatments: Vital signs (blood pressure, pulse, temperature and respiratory rate) will be noted at the following time points:

1st atezolizumab infusion	before infusion start and every 15 minutes (±5 minutes) during infusion and 30 minutes (±10 minutes) after infusion stop.
2nd atezolizumab infusion an onwards	before infusion start and preferably within 60 minutes after infusion stop.<br>**Note:** if clinically indicated OR if the patient experienced an infusion-related reaction with the<br>previous infusion, the vital sign schedule for 1st infusion should be followed.

7.2.7 ECOG Performance Status

Assessed according to the ECOG score (14.2 *Appendix II: ECOG Performance Status*) at screening, before all treatment visits (within -2 days) and at the final follow-up visit. If treatments are discontinued, ECOG score will still be evaluated at all scheduled visits up to week 18(19), and thereafter at the evaluation visits week 27, 36, 45, and at the final follow-up visit week 57 (*14.3 Appendix III: Modified Follow-Up Schedule*).

7.2.8 Pregnancy Test

Urine or serum pregnancy test for females of childbearing potential is to be completed at screening and at the final follow-up visit week 57.

7.2.9 12-Lead ECG

A 12-lead ECG will be performed after 5 minutes rest at screening and at final follow-up visit, and the results will be judged clinically normal or abnormal by the Investigator. Any abnormality will be explained in the eCRF.

7.2.10 Blood Chemistry

Sodium, calcium, phosphate, glucose, ALT, AST, alkaline phosphatase, bilirubin, albumin, creatinine, cystatin-C, lactate dehydrogenase and CRP are taken at screening, within 2 days before all treatment visits, and at final follow-up visit week 57. Prothrombin (INR) or prothrombin time (PT) and partial thromboplastin time (PTT) or activated partial thromboplastin time (aPTT) is analyzed at screening only.

However, if samples have been taken for routine analysis <7 days prior to screening, the results can be used for eligibility evaluation at the discretion of the Investigator, without need of subject the patients for new sampling for this reason.

If treatments are discontinued, samples should still be collected at all scheduled visits up to week 18(19), and thereafter at the evaluation visits week 27, 36, 45, and at the final follow-up visit week 57 (see *14.3 Appendix III: Modified Follow-Up Schedule*).

Blood samples are collected prior to the initiation of pre-medications or treatments according to local hospital routines and sent to the local laboratory for analyses.

IL6 and CRP should be analyzed in case of suspected CRS, see section 9.4.3 *Cytokine Release Syndrome (CRS)*.

7.2.11 Hematology

Hemoglobin, WBC with differential (% and absolute count), platelet count are taken at screening, within -2 days before all treatment visits, and at final follow-up visit week 57.

However, if samples have been taken for routine analysis <7 days prior to screening, the results can be used for eligibility evaluation at the discretion of the Investigator, without need of subject the patients for new sampling for this reason.

If treatments are discontinued, sampling should still be collected at all scheduled visits up to week 18(19), and thereafter at the evaluation visits week 27, 36, 45, and at the final follow-up visit week 57 (see *14.3 Appendix III: Modified Follow-Up Schedule*).

Blood samples are collected prior to the initiation of pre-medications or treatments according to local hospital routines and sent to the local laboratory for analyses.

7.2.12 Concomitant Medications

Concomitant medication includes ongoing medications/therapies, including contraception, at screening as well as between screening and the final follow-up visit.
Concomitant medication should be documented in the concomitant medication section in the eCRF with the generic name, dose, unit, route, frequency of administration, indication (referencing medical history or AE number), as well as start date (if started <2 weeks before screening) and stop date. At any subsequent visits, changes in dose and/or schedule for ongoing medications and new concomitant medications should be recorded, excluding LOAd703 and atezolizumab, which will be registered in the study treatment section of the eCRF. The eCRF is updated at every study visit.

For information about approved and non-approved concomitant medications, see section *5.8 Approved and Non-Approved Concomitant Treatment*.

Women of childbearing potential (i.e. not surgically sterile or had the last menstruation less than 1 year ago) that are study patients or partners to male study patients, should use one of these highly effective contraceptive methods during study treatment:

- combined (estrogen- and progesterone-containing)
- hormonal contraception associated with inhibition of ovulation (oral, intravaginal, transdermal), progesterone-only hormonal contraception associated with inhibition of ovulation (oral, injectable, implantable)
- intrauterine device
- intrauterine hormone-releasing system
- bilateral tubal occlusion
- vasectomized partner
- abstinence of heterosexual intercourse during the entire study period (depending on the preferred and usual lifestyle of the subject).

For female study patients the use of contraceptives should continue until 5 months after last atezolizumab infusion.

Male study patients should use condoms during intercourse during study participation and make sure that his partner, if woman of childbearing potential, use the adequate contraceptives.

7.2.13 Adverse Events (AE) Monitoring

AEs will be monitored continuously during the trial and AE data will be collected regardless of seriousness or causality. See section *9.0 Adverse Events (AE)* regarding AE reporting.

7.3 Immunological Assessments

The immunological analyses are performed by Sponsor at the specified research laboratory (see section *1.7 Laboratories*). The data assessment will be documented in a separate report enclosed to the final eCRF files stored by Sponsor and to the final study report.

Blood sampling is briefly described in section *7.1 Collection of Blood and Tissue Samples and Storage*. Full information is found in the "*LOKON003 Laboratory Manual*" (see InvestigatorSite File).

7.3.1 Anti-Adenoviral Antibodies

Most people have been subjected to adenoviral infections (e.g., upper respiratory tract infections/common cold) and have antibodies against adenovirus. During LOAd703 i.t. treatments,

adenovirus antibody levels often increase but so far there have been no reports associating high levels of antibodies to reduced treatment efficacy. In this trial, the levels of anti-adenovirus antibodies will be monitored during the trial and correlated to clinical and immunological findings. The antibody levels will be determined by enzyme-linked immunosorbent assay (ELISA). Their neutralizing capacity will be determined by a neutralization assay. Samples (immunology sampling; I) are collected pre-dose, weeks 0, 9, 18, 27, 36, 45 and the final follow-up visit week 57.

Valid for Swedish patients: patients enrolled at Uppsala site, Sweden will be asked to provide additional blood samples for research purposes to identify and isolate B-cells producing anti-adenoviral antibodies. Blood samples will be collected at 2-3 occasions (up to 42 ml in total) during the LOAd703 treatment period (starting from week 6 until week 33).

7.3.2 Anti-Drug Antibodies (ADA)

Antibodies can form against atezolizumab and this may increase when atezolizumab is combined with another immunotherapy such as LOAd703. Samples are taken pre-dose, weeks 0, 3, 6, 9, 21 and at final follow-up visit week 57, which corresponds to the atezolizumab infusions 1-4, 8 and the final follow-up visit. ADA will be analyzed even if atezolizumab treatment is discontinued.

7.3.3 Immune Cell Phenotyping

To evaluate the effect of LOAd703 on the immune system (immunology sampling; I), the level of different immune cell populations will be evaluated in samples taken pre-dose, weeks 0, 9, 18, 27, 36, 45 and at the final follow-up visit week 57. The levels of the following immune cell populations will be determined:

- Cytotoxic T cells
- T helper cells
- T regulatory cells
- NK cells
- Myeloid suppressor cells

7.3.4 Cytokines and Other Protein Responses

To evaluate the effect of LOAd703 on cytokine and other protein responses, the levels of various cytokines and other proteins will be determined using single and multiplex assays in serum/plasma taken pre-dose, weeks 0, 9, 18, 27, 36, 45 and at the final follow-up visit week 57 (immunology sampling; I) as well as in tumor biopsies (weeks 0, 9 and 27). Biopsies will also be subjected to nucleic acid array analyses.

7.3.5 Antigen-Specific T Cells

Blood sampling: pre-dose, weeks 0, 9, 18, 27, 36, 45 and at the final follow-up visit week 57 (immunology sampling; I).

When a sufficient number of cells are available from blood samples, we will also analyze the antigen-specific T cell responses by ELISPOT or similar.

7.4 Pharmacokinetics and Virus Shedding

The pharmacokinetics and virus shedding analyses are performed by the Sponsor at the specified research laboratory (see section *1.7 Laboratories*). The data assessment will be documented in separate reports enclosed with the final eCRF files stored by the Sponsor and enclosed with the final study report.

7.4.1 Pharmacokinetics in Blood

LOAd703

Detection of LOAd703 will be performed by quantitative PCR. Blood samples are collected pre-dose (before pre-medication), at weeks 0, 1, 9, 18, 19, 27, 33, 34, 36, 45 and at the final follow-up visit week 57. Samples are taken prior to treatment. Note that post-treatment shedding samples are also analyzed 24 hours (±4h) post-LOAd703 treatment at weeks 0, 9, 18 27 and 33 for patients staying overnight, as well as 7 days (±1 day) post-treatment at weeks 1, 19 and 34.

Atezolizumab

Detection of atezolizumab will be performed at study weeks 0, 3, 6, 9, 21 and at the final follow-up visit week 57 which corresponds to the atezolizumab infusions 1-4, 8 and the final follow-up visit. Samples are taken prior to treatment. Note: at week 0 analysis is performed both before and 30 min (±5 min) after infusion stop.

7.4.2 Presence of LOAd703 in Tumor Lesions

The LOAd703 copy number will be determined by quantitative PCR in biopsies taken at weeks 0, 9 and 27.

7.4.3 Analysis of LOAd703 Shedding in Oral and Rectal Swabs, and Urine

Detection of LOAd703 will be performed by quantitative PCR. Samples are collected pre-dose (before pre-medication), at weeks 0, 1, 9, 18, 27, 33, 36, 45 and at the final follow-up visit week 57. Note that post-treatment shedding samples are also analyzed 24 hours (±4h) post-LOAd703 treatment for patients staying overnight; weeks 0, 9, 18, 27 and 33, as well as 7 days (±1 day) post-treatment in week 1, 19 and 34.

7.5 Efficacy Assessments

Response and tumor progression will be determined for all patients receiving at least one dose of the LOAd703 and atezolizumab investigational drugs. However, patients receiving at least 3 doses of LOAd703 with tumor assessment data available will be considered evaluable for efficacy.

7.5.1 Tumor Size

Tumor size will be determined using a suitable imaging technique, depending on the localization of the tumor. Computer tomography (CT) will preferably be selected. Radiology exams will be performed every 9th week (±1 week) post-treatment initiation (i.e., week 9, 18, 27, 36, 45 and at final follow-up visit week 57). However, at screening, if the patient has been subjected to radiology examination within 3 weeks prior to registration, this imaging can be used as baseline at the discretion of the Investigator. Definitions of measurable disease and response to treatment will follow RECIST 1.1 criteria. For research purposes, other methods such as immune-related (ir) RECIST may be compared to the RECIST v1.1 outcome. Further, a study-specific tumor evaluation will also be performed (see "*Instructions for lesion selection and effect evaluation"* provided by the Sponsor in the Investigator Site File).

Definition of Measurable Disease

Tumor lesions must be accurately measured in at least one dimension (longest diameter in the plane of measurement is to be recorded) with a minimum size of 10 mm by CT scan (irrespective of scanner type), 10 mm caliper measurement by clinical exam (when superficial), or 20 mm by chest X-ray (if clearly defined and surrounded by aerated lung).

PET

In addition to CT/MRI imaging, position emission tomography (PET) may be performed as a part of tumor assessment, at the discretion of the Investigator. If PET is used, the measures are documented according to the EORTC assessment. The PET evaluation will be exploratory.

7.5.2 Tumor Markers

Tumor-specific genetic markers such as BRAF mutation or mutations in DNA repair (i.e., mismatch repair-deficient cancers) will be listed if available during screening. Otherwise, the tumor biopsy (week 0) can be used for analysis. Genetic analysis of the biopsy within the study is done via the Research Laboratory, if applicable or send for analysis within Sweden and EU or third country (US, UK). After the analysis, sample will be destroyed or returned to the biobank.

7.5.3 Survival Follow-Up

After the final clinical follow-up visit at the clinic, overall survival (OS) will be recorded every 4th month (±1 month) if possible. OS will be collected until the trial is closed, i.e until LPLV. OS may be collected via medical records (Sweden, US) or by telephone/email (US) from the Investigator or research nurse/research coordinator to the patient and will consist only of questions regarding the patient's health. If the patient is initiating another anti-cancer treatment, the treatment will be documented if the information is available, since it may affect the OS. The day of death will be documented.

8.0 Response Criteria

8.1 RECIST

The tumor response will be evaluated using an appropriate imaging technique, depending on the localization of the tumor. Tumor regression or progression will be evaluated according to RECIST v1.1 criteria.

Complete Response (CR)

Complete macroscopic disappearance of all tumors.

Partial Response (PR)

A reduction of at least 30% in the sum of all tumor diameters from baseline.

Stable Disease (SD)

Neither PR nor progressive disease.

Progressive Disease (PD)

At least a 20% increase in the sum of all tumor diameters from the smallest tumor size and/or the appearance of new tumor lesion(s).

Confirmation of Progression: Due to the potential immunostimulatory capacity of the study treatments, it is possible that the induced immune stimulation may induce an inflammatory swelling of the tumor that initially may be mistaken for progression. Therefore, PD during study participation leading to discontinuation of repeated treatment needs to be confirmed by radiological imaging at a later time point (within 4-12 weeks) and/or by a biopsy and/or tumor serum marker to confirm tumor progression.

Mixed Response (MR)

One or more lesions fulfilling the criteria for PR and other(s) for PD.

Clinical Benefit Rate (CBR)

CBR is defined as MR or better.

Overall Response Rate (ORR)

ORR is defined as PR plus CR.

Exploratory Response Rates:

a) ORR injected versus non-injected lesions
b) ORR patients with a single injected lesion versus multiple injected lesions
c) ORR patients with low tumor burden versus high tumor burden (>70cm^2)

8.2 OS, TTP and PFS

Time-to-Tumor Progression (TTP)

TTP is the time from start of treatment to disease progression.

Progression-Free Survival (PFS)

PFS is the time from start of the treatment to progression or death.

Overall Survival (OS)

OS is defined as the time from the start of treatment to death due to any cause.

9.0 Adverse Events (AE)

Reference safety information (RSI) for LOAd703 and atezolizumab is listed in the Investigator's Brochure for each study drug. **Note** that both LOAd703 and atezolizumab are regarded as experimental drugs within this protocol and safety evaluation including attribution needs to carefully be considered for both agents.

9.1 Definitions

9.1.1 Adverse Event (AE)

An AE is any untoward medical occurrence that does not necessarily have to have a causal relationship with the treatment. An AE can therefore be any unfavorable, unintended clinical sign, symptom, disease or clinically relevant change in laboratory variables or clinical tests temporally associated with the use of an investigational product, whether or not considered related to the investigation product, that requires clinical intervention or further investigation (beyond ordering a repeat/confirmatory test). Injury or accidents, medical conditions for operations that are not pre-planned, or deterioration of the concurrent illness are also considered as AEs.

9.1.2 Serious Adverse Event (SAE)

A serious adverse event is defined as any untoward medical occurrence that at any dose:

- Results in death.
- Is life-threatening (i.e., the patient was at risk of dying at the time of the event. It does not refer to an event that hypothetically may have caused death if it was more severe).
- Requires in-patient hospitalization or prolongation of existing hospitalization excluding that for pain management, disease staging/re-staging procedures, prolonged monitoring for possible AE, or catheter placement unless associated with other serious events.
- Results in persistent or significant disability or incapacity.
- Is a congenital anomaly or birth defect.

Important medical events that may not result in death, be life-threatening, or require hospitalization may be considered serious adverse drug events when, based on appropriate

medical judgment, they may jeopardize the patient or subject and may require medical or surgical intervention to prevent one of the outcomes listed above.

The term “severe” is often used to describe the intensity (severity) of an event, even if the event itself may be of relatively minor medical significance (e.g., a severe headache). This is not the same as “serious” which is based on patient/event outcome or action criteria usually associated with events that pose a threat to the patient’s life.

Planned or elective hospitalizations (e.g., for administration of protocol therapy) and the hospitalization *per se* should not be considered SAEs. However, should an adverse event occur during this planned or elective hospitalization due to the administration of protocol therapy, it will be regarded as an AE, unless the severity of the event would justify hospitalization, if the patient was not already hospitalized.

9.1.3 Suspected Unexpected Serious Adverse Reactions (SUSAR)

An unexpected adverse event is defined as any adverse drug experience where there is evidence to suggest a causal relationship, the specificity or severity of which is not consistent with the Reference Safety Information (RSI) in the current LOAd703 and/or atezolizumab IBs.

Unexpected, as used in this definition, refers to an adverse drug experience that has not been previously observed (e.g., included in the IB) as opposed to the adverse drug experience not being anticipated from the pharmacological properties of the pharmaceutical product.

9.1.4 Serious Adverse Reactions (SAR)

A serious adverse reaction (SAR) is any AE for which there is evidence to suggest a causal relationship (reasonable possibility) between the study drug and the AE. By definition, all SARs are AEs, nevertheless, not all AEs are SARs. Both LOAd703 and atezolizumab are regarded as study drugs in this study.

9.1.5 Non-Serious Adverse Event

All AEs not fulfilling the previous definitions are classified as non-serious.

9.2 Evaluating and Documenting Adverse Events (AE)

AEs are graded in the study according to the NCI Common Terminology Criteria for Adverse Events (CTCAE) version 5.0 (refer to http://ctep.cancer.gov). All AEs (except for grade 1 and 2 laboratory abnormalities that do not require an intervention) are to be recorded on the AE page in the eCRF and source documentation.

During screening, the Investigator will note the occurrence and nature of each patient’s existing medical condition(s). Occurrence and nature of AEs (including lab events) directly observed by the study personnel or spontaneously reported by the patient during the study will be reported. Each patient will be asked about AEs at each visit after the first dose of the investigational products LOAd703 and/or atezolizumab.

The general AE reporting period for this trial begins upon receiving the first LOAd703 and/or atezolizumab treatment and continues until final clinical follow-up visit at week 57.

If both study treatments are prematurely discontinued, AEs should be recorded until 6 months after the final treatment with LOAd703 or 3 weeks after last atezolizumab infusion, whatever comes last (see *4.4.1 Off-treatment patient*).

If patients are prematurely withdrawn from the study and enter survival follow-up (i.e., survival follow-up patients),they will be followed for a minimum of 30 days after the last LOAd703 and/or

atezolizumab treatment for the occurrence of new or ongoing adverse events (AE), if possible (see *4.4.2 Survival follow up patient*).

If a patient experiences an AE after signing the informed consent, but before treatment is started, the event will be recorded as an existing medical condition unless the Investigator believes that the event may have causal relationship to a study-specific procedure described in the protocol. If LOAd703 and/or atezolizumab have been administrated when an AE occurs, its relationship to the study drugs will be judged by the Investigator.

9.2.1 Severity Grading

The Investigator must determine the intensity of any AEs according to the NCI CTCAE Version 5.0 (see http://ctep.cancer.gov) and the causal relationship. Those AEs not covered by these criteria will be graded as follows:

1. Mild: Discomfort noticed, but no disruption of normal daily activity. Prescription drug not ordinarily needed for relief of the symptom but may be given because of the patient's personality/character.

2. Moderate: Discomfort sufficient enough to reduce or affect normal daily activity. Patient is able to continue in the study; treatment for the symptom may be needed.

3. Severe: Incapacitating, severe discomfort with inability to work or to perform normal daily activity. Severity may cause cessation of treatment with test drug; treatment for symptom may be given and/or patient hospitalized.

4. Life-threatening: Symptom(s) place the patient at immediate risk of death from the reaction as it occurred; does not include a reaction that, had it occurred in a more serious form, might have caused death.

5. Fatal: Event caused the death of the patient.

9.2.2 Attribution Definitions

The Investigator will attempt to assess the relationship of the event to the study drugs separately. An AE is considered to be associated with the use of any or both of the investigational products if the attribution is determined as possible, probable or definite. Attribution of AEs will be recorded in the CRF as:

- Unrelated: The AE is clearly NOT related to LOAd703 (or atezolizumab).
- Unlikely: The AE is doubtfully related to LOAd703 (or atezolizumab).
- Possible: The AE may be related to LOAd703 (or atezolizumab).
- Probable: The AE is likely related to LOAd703 (or atezolizumab).
- Definite: The AE is clearly related to LOAd703 (or atezolizumab).

Attribution will be specified for LOAd703, atezolizumab, treatment procedure (i.e., intratumoral injection and/or intravenous infusion), disease or other.

9.2.3 Duration of Event

The date of onset (and time if relevant), change of severity and the duration of the AE (i.e., date of resolution) will be recorded. Events that are ongoing at the time the patient completes follow-up will be documented as ongoing.

9.2.4 Action(s) Taken Regarding the Study Drugs

The method used to treat the AE, specifically any action taken with the study drugs, should be recorded. This includes, but is not limited to e.g., "dose reduced" or "discontinued".

9.3 Reporting Serious Adverse Events (SAE), Deaths, Unexpected AEs and DLTs

9.3.1 Reporting to Sponsor

AEs classified as serious require expeditious handling and reporting to the CRO as the Sponsor's representative, to comply with regulatory requirements. The Sponsor and its designees must be notified immediately (within 24 hours of becoming aware of the event) by email or telephone. Notification by email is preferred. The email and telephone numbers listed below may be used.

Initial notification via telephone or email does not obviate the need for the Investigator to provide a completed SAE form/eCRF entry within the designated reporting time frames.

DLTs will be handled and recorded in the same way and within the same timelines as SAEs, even if the formal SAE criteria are not fulfilled, A SAE report will be filled in, but if the event does not fulfil the criteria as Serious, only the box for DLT should be ticked.

The completed Serious Adverse Event Report Forms are submitted by email to Precision for Medicine within 24 hours of becoming aware of the event.

The SAE form and detailed instructions describing the procedure for reporting SAEs are found in the Investigator's Site File.

Precision for Medicine:

Email: LOKON003-Safety@precisionformedicine.com
Phone: please see the Investigator Site File

9.3.2 Safety Report – Reporting by Sponsor

The Sponsor is responsible for submitting safety reports to the relevant regulatory authorities and ethics committees of any Suspected, Unexpected Serious Adverse Reactions (SUSARs). A SUSAR that is fatal or life-threatening should be reported as soon as possible, not later than 7 calendar days after the Sponsor becomes aware of the SUSAR, and with a follow-up report within another 8 days. Any other SUSARs should be reported within 15 days.

9.3.2.1 MPA/EMA Safety Report (EU)

The Sponsor is responsible for submitting safety reports to the MPA/EMA and IEC and this will be delegated to the CRO. A SUSAR that is fatal or life-threatening should be reported as soon as possible, not later than 7 calendar days after the Sponsor becomes aware of the SUSAR, and with a follow-up report within another 8 days. Any other SUSARs should be reported within 15 days.

SAEs that do not require expedited reporting should be listed together with SUSARs in the annual drug safety update report (DSUR), written by the Sponsor and submitted to the relevant regulatory authorities and ethics committees according to current legislation.

9.3.2.2 IND Safety Report (US)

Under Title 21 Code of Federal Regulation (CFR) Part 312.32, the Sponsor (or designee) is required to notify the FDA (delegated to B&H) and all participating Investigators (i.e., all Investigators to whom the Sponsor is providing drug under its IND or under any Investigator's IND) of any of the following:

Any SAR, that is both serious and unexpected, and if there is evidence to suggest a causal relationship between the study drug and the event, such as:

- A single occurrence of an event that is uncommon and known to be drug related.
- One or more occurrences of an event that is not commonly associated with the drug but is otherwise uncommon in the population exposed to the drug.
- An aggregate analysis of specific events observed in a clinical trial that indicates those events occur more frequently in the drug-treatment group than in a concurrent or historical control group.

In addition, the Sponsor must report the following:

- Any findings from epidemiological studies, pooled analysis of multiple studies, or clinical studies, whether or not conducted under an IND and whether or not conducted by the Sponsor, that suggest a significant risk in humans exposed to the drug.
- Any findings from laboratory animals or *in vitro* testing, whether or not conducted by the Sponsor, that suggest a significant risk in humans exposed to the drug, such as reports of mutagenicity, teratogenicity, or carcinogenicity, or reports of significant organ toxicity at or near the expected human exposure dose.
- Any clinically important increase in the rate of a serious suspected adverse reaction over that listed in the protocol or IB.

SAEs that do not require expedited reporting should be listed together with SUSARs in the annual DSUR, written by the Sponsor and submitted to the relevant regulatory authorities and ethics committees according to current legislation.

9.3.3 Reporting to IEC/IRB

Investigators must report SAEs and AEs to his/her IRB or IEC per institutional guidelines and/or other applicable guidelines.

9.3.4 Procedures in Case of Medical Emergency

The Investigator should ensure that the necessary procedures and expertise are available to cope with any emergencies during the study.

If an emergency occurs, please notify Precision for Medicine (CRO):

Email: LOKON003-Safety@precisionformedicine.com
Phone: please see the Investigator Site File

9.4 Immunological AEs and Handling Plan

LOAd703 is an immunostimulating agent designed to evoke immune destruction of tumor cells. However, both the virus particle and the immunostimulatory transgenes can initiate immune responses with cytokine production that may affect the patient. Atezolizumab is a so-called checkpoint blockade antibody that restricts normal immunological inhibitory pathways via PD-L1 signaling. Atezolizumab can induce overt immunity.

9.4.1 Immediate Reaction to the LOAd703 Virus Particle

Upon i.t. injection of LOAd703, patients may experience transient fever, chills, nausea, fatigue, vomiting, headache, and increased liver enzymes. They may also have manifestations of CRS including fever, headache, tachycardia and hypotension.

Handling plan: Mild early reactions do not require intervention. Paracetamol or similar anti-inflammatory drugs can be administered to reduce fever and pain if needed. For pain relief, codeine or morphine can be administered as well, at the discretion of the Investigator. For possible severe adverse events, the patient should be transferred to the emergency care unit for intensive monitoring and intervention, which includes blood pressure support, corticosteroids and management of anaphylaxis. In case of CRS grade 2 or higher, tocilizumab can be administered to reduce symptoms, see section 9.4.3 *Cytokine Release Syndrome (CRS)*. For upcoming virus treatments, pre-medication with paracetamol, NSAID and/or low dose corticosteroids can reduce reactions to the virus capsid.

9.4.2 Late Reactions Due to LOAd703 Transgene Expression

During the treatment period, the immunostimulatory transgenes will be expressed locally at the tumor site. This may cause local inflammation and swelling of the injected tumor which may be part of the mechanism-of-action.

Handling plan: Mild reactions do not require intervention. However, if possible swelling leads to severe discomfort and/or pain, paracetamol (or similar anti-inflammatory drug) and/or corticosteroids may be administered. If the inflammation leads to a systemic immunological reaction such as CRS, please refer to the instructions below.

9.4.3 Cytokine Release Syndrome (CRS)

Cytokine release syndrome (CRS) has been observed after administration of immunotherapy and can be life-threatening. Clinical signs and symptoms associated with CRS are various (i.e., fever, nausea, fatigue, myalgias, malaise, tachypnea, headache, tachycardia, hypotension, rash, and/or hypoxia) but the most severe includes **hypotension** requiring vasopressors and **hypoxia** requiring high-flow oxygen by nasal cannula, face mask, non-rebreather mas or venturi mask. CRS occurs when immune cells are activated and release cytokines. One of the main CRS-driving cytokines is IL6, which causes release of C reactive protein (CRP) from the liver.

Handling plan: Mild CRS (grade 1 symptoms including fever, nausea, fatigue, headache, myalgias, and malaise) do not require intervention although symptomatic treatment may be given. For possible severe CRS (CTCAE grade 2-4), the patient should be transferred to the emergency care unit if there is a need for intensive monitoring and intervention, which may include blood pressure support, corticosteroids and management of anaphylaxis. Tocilizumab (antibody targeting IL6 receptor) can be administered at 4 or 8 mg/kg IV over 1 hour with an option to repeat the dose if clinical improvement does not occur within 24 to 48 hours.

CRS grade 2 or higher should be confirmed by measuring IL6 and CRP at the time of the event and before releasing the patients from the hospital. If the CRS is in response to virus administration, for upcoming virus treatments, pre-medication with paracetamol, NSAID and/or low dose corticosteroids can reduce reactions to the virus capsid.

More information about CRS in relation to atezolizumab can be found in the atezolizumab IB that is an integral part of this protocol.

9.4.4 Immediate - Related Reactions to Atezolizumab

Patients who experience infusion-associated symptoms may be treated symptomatically with acetaminophen, ibuprofen, diphenhydramine, and/or H2-receptor antagonists (e.g., famotidine, cimetidine), or equivalent medications per local standard practice. Serious infusion-associated events manifested by dyspnea, hypotension, wheezing, bronchospasm, tachycardia, reduced oxygen saturation, or respiratory distress should be managed with supportive therapies as clinically indicated (e.g., supplemental oxygen and β_2-adrenergic agonists). Pre-medication with

antihistamines, antipyretics, and/or analgesics may be administered for the second and subsequent atezolizumab infusions only, at the discretion of the Investigator.

More information about infusion-related reactions in relation to atezolizumab can be found in the atezolizumab IB that is an integral part of this protocol.

9.4.5 Late Reactions to Atezolizumab

Atezolizumab has been associated with risks such as the following: immune-related reactions (IRR) and immune-related hepatitis, pneumonitis, colitis, pancreatitis, diabetes mellitus, hypothyroidism, hyperthyroidism, adrenal insufficiency, hypophysitis, Guillain-Barré syndrome, myastenic syndrome or myasthenia gravis, meningoencephalitis, myocarditis, nephritis, severe cutaneous adverse reactions (SCARs) immune-mediated pericardial disorders, immune-mediated myelitis and facial paresis. In addition, systemic immune activation is considered a potential risk for atezolizumab.

Dose modifications and omissions due to toxicity will be instituted per standard guidelines. (US: TECENTRIQ® Full Prescribing Information, Reference ID:4279345, Sweden: SmPC/FASS for atezolizumab/TECENTRIQ®). The symptoms of late reactions will be treated as per clinical practice. More information about handling of immune-related reactions in relation to atezolizumab can be found in the atezolizumab IB that is an integral part of this protocol.

9.5 Handling of Pregnancy

Any pregnancy diagnosed during the study participation must be reported immediately to the Sponsor. Women of childbearing potential and male with a partner of child bearing potential, will be thoroughly informed about contraceptive medication (see section 7.2.12 *Concomitant Medications*) before trial participation and to immediately inform the Investigator if pregnancy should occur during study participation. Pregnancy, in and of itself, is not regarded as an AE, unless there is suspicion that study medication may have interfered with the effectiveness of a contraceptive medication. If the patient becomes pregnant while participating in the study, the study drugs should be immediately discontinued. Pregnancy information about a female patient or a female partner of a male patient should be reported immediately from the time the Investigator first becomes aware of a pregnancy or its outcome. This will be performed by the Investigator by completing a Pregnancy Form.

Any pregnancy complication, spontaneous abortion, elective termination of a pregnancy for medical reasons, outcome of stillbirth, congenital anomaly/birth defect, or SAE in the mother will be recorded as an SAE and reported.

10.0 STATISTICS

10.1 Statistical Analysis Plan

A Statistical Analysis Plan (SAP) will be prepared as a separate document and will include a more technical and detailed description (including templates for Tables, Listings, and Figures) of the planned statistical summaries regarding safety and effect evaluation. The SAP will be finalized before initiating any statistical analysis. Unless otherwise stated, tabulation of summary statistics and data analysis will be performed using SAS® Version 9 or later.

Statistical Methods

Statistics will be displayed for response evaluation for the patients treated at MTD.

Continuous variables will be summarized by descriptive statistics (e.g., n, arithmetic mean, standard deviation, min, median and max).

Demographic and efficacy analyses will be carried out using all toxicity and response evaluable patients.

Patient disposition, including reason for withdrawal, will be summarized by dose group.

Demographics and disease baseline characteristics, pregnancy test results, medical history and prior medications will be tabulated.

10.2 Sample Size

10.2.1 Safety

A patient receiving at least one dose of LOAd703 and/or atezolizumab will be included in the safety assessment. However, the DLT period encompasses at least two doses of LOAd703 and/or atezolizumab plus at least three weeks of AE evaluation post the second dose.

The study will be conducted using a Bayesian Optimal Interval (BOIN) design with a target DLT rate of 0.3.[39] This design has algorithmic escalation/de-escalation rules like a traditional 3+3 design but allows for specification of the target DLT rate and expanded accrual beyond N=6 with continued consistent toxicity monitoring (i.e., the assessment of DLT rate continues throughout the study which gives a well-defined safety evaluation of Phase I/II studies).

Two LOAd703 dose levels will be tested together with a fixed dose of atezolizumab. The BOIN design allows for enrollment of 3 patients in a cohort and the number of patients with DLT determines whether additional patients are treated with the current dose or if the dose is decreased or increased. In general, we will escalate the dose if the observed toxicity rate at the current dose is <= 0.2365, and we will de-escalate the dose if the observed toxicity rate at the current dose is >= 0.3585. If the observed toxicity rate is between 0.2365 and 0.3585, 3 additional patients will be treated at the current dose. For cohorts of size 3, the decision boundaries are shown in Table 3.

Additional cohorts will be added in the unlikely event that more than N=25 participants at a single dose level are needed.

Table 2. DLT Regulation of Dose Escalation

Number of Patients treated in a Defined Dose Cohort	3	6	9	12	15	18	21	24	25
Escalate dose if number of DLT≤	0	1	2	2	3	4	4	5	5
Treat additional 3 patients at current dose if number of DLT=	1	2	3	3,4	4,5	5,6	5-7	6-8	6-8
De-escalate dose if number of DLT≥*	2	3	4	5	6	7	8	9	9
Eliminate** dose level and all higher doses from further use if number of DLT≥	3	4	5	7	8	9	10	11	12

* At the lowest dose level, if the recommendation is to de-escalate AND the dose level has not been eliminated from consideration, 3 additional patients can be accrued at the same dose level; otherwise the trial will be stopped.
** Note that if the DLT level reach the elimination limit, this dose level is abandoned from the study independently of DLT events at lower dose levels[39].

10.2.2 Effect

A patient receiving at least three doses of LOAd703 and/or atezolizumab with available tumor assessment data will be included in the effect evaluation.

Efficacy will be evaluated using a one-sided binominal test. Assuming a true response rate of at least 30%, then at least 25 subjects are needed to be evaluated at MTD to have a probability of 0.9 of rejecting the null hypothesis (i.e., that the response rate is lower or equal to 5%) using a one-sided test with a significance level of 0.025 (see Table 3).

Table 3. Specifications from the Sample Size Calculation

Predicted response rate	N, total	Critical value*	Actual alpha	Power
30%	25	5	0.0072	0.910

*The critical value is the lowest number of responders needed to reject the null hypothesis.

10.3 Safety Reporting

Summary statistics will be presented descriptively for the following safety endpoints by dose group:

- Adverse Events - the number of adverse events, the proportion of patients having at least one adverse event and adverse events by MedDRA® coded terms will be presented.
- Serious Adverse Events - the number of serious adverse events, the proportion of patients having at least one serious adverse event and serious adverse events by coded terms will be presented.
- Related Adverse Events - the number of related adverse events to LOAd703 or atezolizumab, the proportion of patients having at least one related adverse event and related adverse events by coded terms will be presented separately and pooled.

Note: Only treatment emergent AEs (commencing after exposure to study medication) will be included in the AE summaries. Non-treatment emergent events (starting prior to exposure to study medication) will be included in the patient listings and not included in the above summaries.

- Clinical Laboratory Evaluation values and changes from baseline for all parameters will be presented, where baseline is the last recorded value prior to first dose.

Note: Values outside normal ranges will be flagged in the individual patient listings.

- Results of quantitative and qualitative evaluations for urinalysis will be presented in a similar way as the laboratory parameters.
- Vital sign parameters including changes from baseline
- ECOG including changes from baseline

10.4 Efficacy Reporting

Efficacy will be tabulated by response category based on the RECIST v1.1 criteria for the efficacy evaluable population. The clinical benefit rate (CBR) and overall response rate (ORR) will be determined. For analysis of the overall response rates, summary tables will be generated, presenting the number and proportion of responders in each treatment group, together with point estimates and two-sided 95% Pearson-Clopper confidence intervals (CIs).

For the variables of progression-free survival (PFS), time-to-progression (TTP) and overall survival (OS), Kaplan-Meier curves and estimates including medians and 95% CIs will be provided.

10.5 Immunological Parameters

The immunological parameters such as levels of certain immune cell types, the presence of cytokines and the development of anti-adenovirus antibodies/atezolizumab ADAs are evaluated at baseline and at different time points during and after treatments. The results will be compared to baseline levels and statistical differences will be estimated, after appropriate transformation using general linear mixed models. Immunological values and changes will be correlated to clinical responses using Spearman's rank correlation.

10.6 Pharmacokinetics and Shedding

10.6.1 Pharmacokinetics

The presence of LOAd703 viral particles in blood and biopsies will be evaluated at baseline and at different time points during and after LOAd703 treatment. The results will be compared to baseline levels and statistical differences will be estimated, after appropriate transformation using general linear mixed models. The levels of viral particles may be correlated to clinical response parameters using the Spearman correlation test for nonparametric testing.

The presence of atezolizumab in blood will be evaluated at baseline at different time points during and after atezolizumab treatment. The results will be compared to baseline levels and statistical differences will be estimated, after appropriate transformation using general linear mixed models.

10.6.2 LOAd703 Shedding

The presence of LOAd703 viral particles in oral cavity, feces and urine are evaluated at baseline and at different time points during and after LOAd703 treatments to understand possible shedding and route. The results will be compared to baseline levels and statistical differences will be estimated, after appropriate transformation using general linear mixed models. The levels of viral particles may be correlated to clinical response parameters using the Spearman correlation test for nonparametric testing.

11.0 QUALITY CONTROL AND QUALITY ASSURANCE

11.1 Direct Access to Source Data/Documents

The Investigator(s)/institution(s) will permit study-related monitoring, audits, review and regulatory inspection(s) and will provide access to source data/hospital records. The Sponsor verifies that each patient has consented in writing to grant access to the original source data/hospital records by means of the written patient information and signed informed consent.

During monitoring, the data recorded in the CRFs by the Investigator or designee will be checked for consistency with the source data/hospital records by the Monitor (i.e., source data verification). Any data discrepancies will be documented and explained in the monitoring reports.

11.2 Source Data

The requirements for the information contained in the patient's medical records corresponds to the requirements of the "Patientdatalagen" (SFS 2008:355), "The Medical Product Agency's regulations on clinical trials of medicinal products for human use" (LVFS 2011:19), and Department of Health and Human Services Standards for Privacy of Individually Identifiable Health Information, 45 CFR 164.508 which means that in addition to information that is pertinent to the care and well-being of the patient, the following minimum study-specific information must be recorded:

- Date when patient information was given and when signed informed consent was obtained
- Patient study number
- The name of the study, EudraCT /IND/NCT number and short study description
- Fulfilment of inclusion criteria
- Diagnosis
- Dates of all visits during the study period
- Any information relating to AEs
- All treatments and medications prescribed/administered (including dosage)
- Date of study termination

For information that is study specific and of no interest to the medical care of the patient, other documents may be considered as source data. Prior to study start, the site Principal Investigator/study nurse/coordinator and the Monitor must identify and document the expected location of the source data (e.g., medical record notes, laboratory reports, etc.). This will be done by completing a site-specific source data verification log (Origin of Source Data). The site must clearly communicate any deviation from the expected source data location to the Monitor.

11.3 Monitoring

In accordance with the principles of Good Clinical Practice (GCP), the Sponsor is responsible that the trial is adequately monitored. In this study, the CRO will be responsible for monitoring activities on behalf of the Sponsor. During the study, the Monitor will have regular contacts with the study site, including monitoring visits to ensure that the study is conducted and documented properly in compliance with the protocol, GCP and applicable regulatory requirements.

The Monitor will ensure that accountability of investigational products is performed and will review source documents to verify consistency with the data recorded in the CRFs. All patients that have performed any study-specific assessments will be monitored for signed informed consent and all patients that have received study drug will be monitored for date of study visits, inclusion/exclusion criteria and AE/SAE.

The Monitor will also check the Investigator Site File and provide information and support to the Investigator(s). The extent of monitoring will be described in a monitoring plan, which will be approved by the Sponsor.

The study site may be subject to quality assurance audit(s) by the Sponsor as well as inspection by regulatory authorities. The Investigator and other responsible personnel must be available during the monitoring visits, audits and inspections and should devote sufficient time to these processes.

<u>For investigational sites in the EU</u>: All site Principal Investigators should provide a curriculum vitae (CV) to the CRO to be responsible for the study. All Sub-Investigators and other responsible personnel should be listed on the delegation list, together with their study responsibilities, and provide their CVs to the CRO. GCP certificates for the Principal Investigator and all study team members on the delegation log must be provided.

<u>For investigational sites in the US</u>: All site Principal Investigators will be required to provide a current signed and dated CV, medical license, proof of human subject research protections training, a completed FDA Form 1572 (required in the US) and a financial disclosure statement (required in the US) to the CRO. All Sub-Investigators will be required to provide a current NIH biosketch or CV, medical license, proof of human subject research protections training and a financial disclosure statement (required in the US) to the CRO. GCP certificates for the Principal Investigator and all study team members on the delegation log must be provided.

12.0 ETHICS

12.1 Protocol Modifications

No modification of the protocol should be implemented without the prior written approval of the Sponsor. Any such changes, which may affect a patient's treatment or informed consent, especially those increasing potential risks or scientific quality of the study, must receive prior approval by the relevant regulatory authorities/ethics committees before implementation. The exception to this is where modifications are necessary to eliminate an immediate hazard to trial subjects, or when the change involves only logistical or administrative aspects of the trial (e.g., change in Monitor or telephone number). Other administrative revisions, which may impact the clinical portion of the study, will be duly reported to the regulatory authority by the Sponsor and/or ethics committee by

the site Principal Investigator, or designee, under the guidance of the CRO and with the approval of the Sponsor.

12.2 Independent Ethics Committee (IEC)/Institutional Review Board (IRB)

It is the responsibility of the site Principal Investigator to obtain approval of the study protocol, protocol amendments, patient information and informed consent from the relevant IEC/IRB before enrollment of any subject into the study.

The Sponsor or designees shall report all SUSARs to the IEC/MPA. If a study stops prematurely at a study center for any reason, the IEC must be informed. At the end of the study, the Sponsor/site Principal Investigator should notify the IEC.

The Sponsor/site Principal Investigator should file all correspondence with the relevant IEC/IRB and provide the other party with copies.

12.3 Ethical Conduct of the Study

The study will be conducted in accordance with the protocol, applicable regulatory requirements, GCP, and the ethical principles of the latest version of the Declaration of Helsinki.

12.4 Patient Information and Informed Consent

It is the responsibility of the Investigator to provide each subject with full and adequate verbal and written information about the objectives, procedures and possible risks and benefits of the study. All subjects should be given the opportunity to ask questions about the study and should be given sufficient time to decide whether or not to participate in the study. The written patient information must not be changed without prior discussion with the Sponsor.

The subjects will be notified of their voluntary participation and of their freedom to withdraw from the study at any time and without giving any particular reason. Subjects must also be informed that withdrawing from the study will not affect their future medical care, treatment or benefits to which the subject is otherwise entitled.

The Investigator is responsible for obtaining written informed consent from all subjects (or their legally acceptable representatives and/or witnesses, where applicable) prior to enrollment in the study.

Upon signing informed consent, the subject consents to:

- Participating in the study.
- Allowing personnel connected with the Sponsor or regulatory authorities to gain full access to hospital records to check study data.
- Recording, collection, processing and storing of data in a database.
- Possible transfer of study information to countries outside of the European Union (EU)/United States (US).
- Storing of study samples in a biobank when required (i.e., European sites require a biobank).

It should be clearly stated that the data will not identify any subject taking part in the study, in accordance with the national guidelines for handling personal data.

The Investigator who gave the verbal and written information to the subject shall sign and date the informed consent form. A copy of the patient information and the informed consent form should be given to the subject. The Investigator should file the signed informed consent forms in the Investigator Site File for possible future audits and inspections. The Investigator Site File shall be archived according to the applicable regulations.

13.0 DATA MANAGEMENT

13.1 Data Management

The CRO is responsible for Data Management and will write a study-specific Data Management Plan (DMP) where further details will be specified. All data will be recorded in electronic CRFs (eCRFs). The Investigator is responsible for ensuring the accuracy, completeness and legibility of the data reported in the eCRFs, and this will be checked by the Monitor.

13.1.1 Data Entry and Data Validation

Data will be entered into the study eCRF and will be subject to both logical computerized checks and manual validation checks against listings in accordance with the study-specific DMP. All inconsistencies detected during these procedures will be resolved through electronic Data Clarification Forms (DCFs) in the eCRF, issued to the Monitor or investigational site personnel.

13.1.2 Database Closure

When all patients have been completed, all data have been entered into the eCRF database, all coding is done and approved, and all queries are solved, the Database Closure procedures will begin. Decisions will be made on how to classify patients into analysis populations, and how to handle protocol violations and deviating or missing data. All decisions will be dated and documented in a Database Closure document. After finalization of the Database Closure document, the database will be locked. Any changes in the database thereafter will be documented.

13.2 Electronic Case Report Forms (eCRFs)

The Investigator is responsible for ensuring the accuracy, completeness, legibility and timeliness of the data recorded in the eCRFs. An eCRF is required and should be completed for each included subject on a visit-by-visit basis. The eCRF should be completed, monitored and corrected (if needed) within 6 months from the last visit of the study patient. The subject's identity must always remain confidential. All information in the eCRFs should be in English. If necessary, the Monitor should translate any information or comments recorded in other languages.

The completed eCRFs are the sole property of the Sponsor and should not be made available in any form to third parties (except for authorized representatives of relevant regulatory authorities) without written permission from the Sponsor. All eCRF electronic files should be kept in the Sponsor's Trial Master File and a copy of all eCRFs electronic files should be kept in the Investigator Site File.

In this study, clinical data including AEs and concomitant medications will be entered into a 21 CFR Part 11-compliant eCRF (RAVE EDC) provided by Medidata. The eCRF includes password protection, audit trails and internal quality checks, such as automatic range checks to identify data that appear inconsistent, incomplete, or inaccurate. Clinical data will be entered directly from the source documents. Backups of the data are automatically performed on a regular basis. Backups are stored and encrypted on both magnetic and optical media in a separate physical location. This process guarantees the minimum data loss in the events of a disastrous failure.

13.3 Record Keeping

To enable audits and evaluations by the Sponsor and inspections by regulatory authorities, the Investigator shall keep records (essential documents) of the study for at least 10 years after final report. This includes any original source data related to the study, the subject identification list (with subject identification numbers, full names and addresses), original signed informed consent forms, copies of all eCRF-generated files and detailed records of the investigational product's disposition.

13.4 Study Report

The survival follow-up will continue until LPLV and the study is thereafter completed.

A full study report should be submitted to the regulatory authorities by the Sponsor within 12 months from the completion of the study. The site Principal Investigator and study site personnel must provide the necessary information by completing the eCRF for every patient no later than 3 weeks from the LPLV to allow for close-out activities such as monitoring and correction of the eCRFs if necessary.

13.5 Publication Policy

The investigation is considered as a collaboration between the clinical sites, Hoffmann LaRoche Ltd and the Sponsor. Publication ideas for manuscripts, abstracts or presentations will be jointly discussed and prepared. All manuscripts, abstracts or presentations (in outline form with copies of slides, if available) will be submitted to the Sponsor at least 30 days prior to the submission of the data for publication in order for the Sponsor to protect proprietary information. The Sponsor will review the submitted material within a reasonable period of time and will not unreasonably withhold publication permission. Investigators as well as employees or representatives of the Sponsor and Hoffmann LaRoche Ltd should be listed as co-authors in the event that they have provided scientific input into the study design, data interpretation, etc.

13.6 Insurance

Study patients enrolled in Sweden are covered by the Swedish Patient Insurance (LÖF) and the Swedish Pharmaceutical Insurance, in which Nxt2b (the parent company of Lokon Pharma) is a shareholder. Study patients enrolled in the US are insured by Chubb, held by Sponsor.

14.0 APPENDICES

1 Atezolizumab pharmacokinetic sample is taken pre-treatment and at the first infusion also 30 min (± 5min) post infusion stop.
2 Shedding sample taken 7 days (± 1 day) post LOAd703.
3 Shedding sample at 24h (±4 hours) is taken if patients are staying overnight.
4 Atezolizumab should preferably be infused within 1 hours post LOAd703 injection.
5 LOAd703: Heart rate, blood pressure and temperature to be noted pre-dose, as well as 30 min, 60 min, 90 min (±5 minutes), 2h, 3h, 4h 5h, 6h, 8h (±10 minutes) and 10h. Thereafter every 4 hours (14h, 18h, 22h, 26h, 30h (±30 minutes)) etc. post injection at the discretion of Investigator and until a final measurement of vital signs at discharge.
6 Atezolizumab: Heart rate, blood pressure, temperature and respiratory rate to be noted pre-dose, every 15 minutes (±5 minutes) during infusion, and 30 min (±10 minutes) after infusion stop (1st treatment and if infusion-related toxicity is observed), or pre-dose and within 60 min of atezolizumab infusion stop.
7 Heart rate, blood pressure, pulse on modified FU visits.
8 Baseline: RECIST evaluation of CT ≤3week prior to registration can be used.
9 Results from samples taken for routine analysis <7 days prior to screening, can be used for eligibility evaluation at the discretion of the Investigator
10 Modified follow-up (±1 week)
11 Survival follow-up (±1 month)

14.2 Appendix II: ECOG Performance Status

Grade	Description
**0**	Fully active, able to carry on all pre-disease performance without restriction.
**1**	Restricted in physically strenuous activity but ambulatory and able to carry out work of a light or sedentary nature, e.g., light housework, office work.
**2**	Ambulatory and capable of all self-care but unable to carry out any work activities. Up and about more than 50% of waking hours.
**3**	Capable of only limited self-care, confined to bed or chair more than 50% of waking hours.
**4**	Completely disabled. Cannot carry out any self-care. Totally confined to bed or chair.
**5**	Dead

14.3 Appendix III: Modified Follow-Up Schedule

	DLT period																						
	Study week																						
	0	1	3	6	9	12	15	18	19	21	24	27	30	33	34	36	39	42	45	48	51	54	57 final FU	Survival FU every 4m
	LOAd703 treatment period																							
	Atezolizumab treatment period																							
On-treatment (receiving LOAd703 and/or Atezolizumab)	X											*												
			X									*												
				X												*								
					X											*								
						X										*								
							X												*					
								X											*					
										X									*					
											X												*	
												X											*	
													X										*	
														X									*	
																X							*	
																	X						*	
																		X					*	
																			X				*	
																				X			*	
																					X		*	
																						X	*	

X - indicates the last visit where treatments (LOAd703 and/or Atezolizumab) were received

■ - indicates visits that patients are still expected to attend when off-treatment (=both LOAd703 and Atezolizumab are discontinued), as long as they have not moved on to another treatment (=survival follow-up).

* - represents the point up to which **AEs should be followed** following the end of treatment (for at least 24 weeks after last LOAd703 dose) and also equates to when **the final FU visit should be performed** based on when the last treatment was received.

e.g., For a patient only completing 2 cycles of treatment, the patient will come to all visits up to week 18 and then week 27, which is 24 weeks after last dose. There would be no expectation for them to follow the modified schedule at weeks 36, 45 and 57.

15.0 REFERENCES

1. Medina-Echeverz J, Aranda F, Berraondo P. Myeloid-derived cells are key targets of tumor immunotherapy. In: *Oncoimmunology.* Vol 3. United States2014:e28398.
2. Whiteside TL. Regulatory T cell subsets in human cancer: are they regulating for or against tumor progression? *Cancer Immunol Immunother.* 2014;63(1):67-72.
3. Lee S, Margolin K. Cytokines in cancer immunotherapy. *Cancers (Basel).* 2011;3(4):3856-3893.
4. Baksh K, Weber J. Immune checkpoint protein inhibition for cancer: preclinical justification for CTLA-4 and PD-1 blockade and new combinations. *Semin Oncol.* 2015;42(3):363-377.
5. Maus MV, Grupp SA, Porter DL, June CH. Antibody-modified T cells: CARs take the front seat for hematologic malignancies. *Blood.* 2014;123(17):2625-2635.
6. Vacchelli E, Eggermont A, Sautes-Fridman C, et al. Trial watch: Oncolytic viruses for cancer therapy. *Oncoimmunology.* 2013;2(6):e24612.
7. Alemany R. Chapter four--Design of improved oncolytic adenoviruses. *Adv Cancer Res.* 2012;115:93-114.
8. Rojas JJ, Guedan S, Searle PF, et al. Minimal RB-responsive E1A promoter modification to attain potency, selectivity, and transgene-arming capacity in oncolytic adenoviruses. *Mol Ther.* 2010;18(11):1960-1971.
9. Andtbacka RH, Kaufman HL, Collichio F, et al. Talimogene Laherparepvec Improves Durable Response Rate in Patients With Advanced Melanoma. *J Clin Oncol.* 2015;33(25):2780-2788.
10. Fueyo J, Gomez-Manzano C, Alemany R, et al. A mutant oncolytic adenovirus targeting the Rb pathway produces anti-glioma effect in vivo. *Oncogene.* 2000;19(1):2-12.
11. Ullenhag G, Loskog AS. AdCD40L--crossing the valley of death? *Int Rev Immunol.* 2012;31(4):289-298.
12. Eriksson E, Moreno R, Milenova I, et al. Activation of myeloid and endothelial cells by CD40L gene therapy supports T-cell expansion and migration into the tumor microenvironment. *Gene Ther.* 2017;24(2):92-103.
13. Eriksson E, Milenova I, Wenthe J, et al. Shaping the Tumor Stroma and Sparking Immune Activation by CD40 and 4-1BB Signaling Induced by an Armed Oncolytic Virus. *Clin Cancer Res.* 2017;23(19):5846-5857.
14. Li X, He C, Liu C, et al. Expansion of NK cells from PBMCs using immobilized 4-1BBL and interleukin-21. *Int J Oncol.* 2015;47(1):335-342.
15. Lynch DH. The promise of 4-1BB (CD137)-mediated immunomodulation and the immunotherapy of cancer. *Immunol Rev.* 2008;222:277-286.
16. Ribas A, Dummer R, Puzanov I, et al. Oncolytic Virotherapy Promotes Intratumoral T Cell Infiltration and Improves Anti-PD-1 Immunotherapy. *Cell.* 2017;170(6):1109-1119.e1110.
17. Blank C, Mackensen A. Contribution of the PD-L1/PD-1 pathway to T-cell exhaustion: an update on implications for chronic infections and tumor evasion. *Cancer Immunol Immunother.* 2007;56(5):739-745.
18. Butte MJ, Keir ME, Phamduy TB, Sharpe AH, Freeman GJ. Programmed death-1 ligand 1 interacts specifically with the B7-1 costimulatory molecule to inhibit T cell responses. *Immunity.* 2007;27(1):111-122.
19. Yang J, Riella LV, Chock S, et al. The novel costimulatory programmed death ligand 1/B7.1 pathway is functional in inhibiting alloimmune responses in vivo. *J Immunol.* 2011;187(3):1113-1119.
20. Fehrenbacher L, Spira A, Ballinger M, et al. Atezolizumab versus docetaxel for patients with previously treated non-small-cell lung cancer (POPLAR): a multicentre, open-label, phase 2 randomised controlled trial. *Lancet.* 2016;387(10030):1837-1846.

21. Rosenberg JE, Hoffman-Censits J, Powles T, et al. Atezolizumab in patients with locally advanced and metastatic urothelial carcinoma who have progressed following treatment with platinum-based chemotherapy: a single-arm, multicentre, phase 2 trial. *Lancet.* 2016;387(10031):1909-1920.
22. Loskog A, Maleka A, Mangsbo S, et al. Immunostimulatory AdCD40L gene therapy combined with low-dose cyclophosphamide in metastatic melanoma patients. *Br J Cancer.* 2016;114(8):872-880.
23. Small EJ, Carducci MA, Burke JM, et al. A phase I trial of intravenous CG7870, a replication-selective, prostate-specific antigen-targeted oncolytic adenovirus, for the treatment of hormone-refractory, metastatic prostate cancer. *Mol Ther.* 2006;14(1):107-117.
24. Griscelli F, Opolon P, Saulnier P, et al. Recombinant adenovirus shedding after intratumoral gene transfer in lung cancer patients. *Gene Ther.* 2003;10(5):386-395.
25. Dummer R, Bergh J, Karlsson Y, et al. Biological activity and safety of adenoviral vector-expressed wild-type p53 after intratumoral injection in melanoma and breast cancer patients with p53-overexpressing tumors. *Cancer Gene Ther.* 2000;7(7):1069-1076.
26. Kim KH, Dmitriev I, O'Malley JP, et al. A phase I clinical trial of Ad5.SSTR/TK.RGD, a novel infectivity-enhanced bicistronic adenovirus, in patients with recurrent gynecologic cancer. *Clin Cancer Res.* 2012;18(12):3440-3451.
27. Kim KH, Dmitriev IP, Saddekni S, et al. A phase I clinical trial of Ad5/3-Delta24, a novel serotype-chimeric, infectivity-enhanced, conditionally-replicative adenovirus (CRAd), in patients with recurrent ovarian cancer. *Gynecol Oncol.* 2013;130(3):518-524.
28. Makower D, Rozenblit A, Kaufman H, et al. Phase II clinical trial of intralesional administration of the oncolytic adenovirus ONYX-015 in patients with hepatobiliary tumors with correlative p53 studies. *Clin Cancer Res.* 2003;9(2):693-702.
29. Guleria I, Khosroshahi A, Ansari MJ, et al. A critical role for the programmed death ligand 1 in fetomaternal tolerance. *J Exp Med.* 2005;202(2):231-237.
30. Habicht A, Dada S, Jurewicz M, et al. A link between PDL1 and T regulatory cells in fetomaternal tolerance. *J Immunol.* 2007;179(8):5211-5219.
31. D'Addio F, Riella LV, Mfarrej BG, et al. The link between the PDL1 costimulatory pathway and Th17 in fetomaternal tolerance. *J Immunol.* 2011;187(9):4530-4541.
32. American Society of Clinical Oncology, Melanoma Statistics. https://www.cancer.net/cancer-types/melanoma/statistics. Accessed June, 2019.
33. Melanoma Research Alliance, Melanoma Statistics. https://www.curemelanoma.org/about-melanoma/melanoma-statistics-2/. Accessed June, 2019.
34. American Cancer Society, Melanoma Risk Factors. https://www.cancer.org/cancer/melanoma-skin-cancer/causes-risks-prevention/risk-factors.html. Accessed June, 2019.
35. Center for Disease Control, Melanoma Cancer. https://www.cdc.gov/media/releases/2015/p0602-melanoma-cancer.html. Accessed June, 2019.
36. Center for Disease Control, Melanoma Cancer Incidence. https://www.cdc.gov/media/releases/2015/p0602-melanoma-cancer.html. Accessed June, 2019.
37. Yu C, Liu X, Yang J, et al. Combination of Immunotherapy With Targeted Therapy: Theory and Practice in Metastatic Melanoma. *Front Immunol.* 2019;10:990.
38. Rogiers A, Boekhout A, Schwarze JK, Awada G, Blank CU, Neyns B. Long-Term Survival, Quality of Life, and Psychosocial Outcomes in Advanced Melanoma Patients Treated with Immune Checkpoint Inhibitors. *J Oncol.* 2019;2019:5269062.
39. Liu S, Johnson VE. A robust Bayesian dose-finding design for phase I/II clinical trials. *Biostatistics.* 2016;17(2):249-263.
