# Supplementary material for: LOAd703-induced tumor microenvironment gene engineering in combination with atezolizumab in metastatic malignant melanoma: a phase I/II trial
Source: Nat Commun. 2026 Feb 16;17:1760. doi: 10.1038/s41467-026-69629-0 (PMC12913765; doi:10.1038/s41467-026-69629-0)
Supplement: Supplementary file 2 — Description of Additional Supplementary Files [file 41467_2026_69629_MOESM2_ESM.pdf]

Title: Supplementary Data 1

Description: Adverse events, post-hoc efficacy data, biomarker significances, and study protocol

Title: Supplementary Data 2

Description: biomarker significances for NanoString
